# Supplementary material for: Beyond the jab: Unravelling the complexities of vaccine adoption for East Coast Fever in rural Kenya
Source: PLoS One. 2025 Jan 28;20(1):e0315906. doi: 10.1371/journal.pone.0315906 (PMC11774369; doi:10.1371/journal.pone.0315906)
Supplement: S1 Dataset — (ZIP) [file pone.0315906.s001.zip › Supporting information (R)/KIIs/230719-1744.docx]

KII Animal Health Officer

Researcher: Thank you very much for allowing us to interview you. You can begin by giving us an overview of what you practice. I understand that you work in the animal health industry. Could you tell us about your occupation, how many years you have practised, and your experiences?

Interviewee: I am a resident of Narok. I work there. I was born here, and I work here. Professionally, I am an animal health officer dealing in biotech and agriculture. I know general agriculture and biotechnology and have been working with people from the area for the last three years in agronomy and livestock management. I mainly deal with livestock management, which I have been doing for nine months as I identified this gap between the farmers in this area. So, I have been seriously engaging the pastoral communities. Livestock involves diseases and many other things, from diseases to range and pen management.

Researcher: In the three years you have been working in this area, what are the three diseases the farmers in this area usually complain about that their livestock are infected with?

Interviewee: The livestock diseases differ because we have the diseases that the specific animals are infected with, which are the sheep, goats, and cows. I have named these three animals because they are the primary animals that are reared in this area, and they are the animals that the Maasai have. So, with the goats, you will find they are infected with Pleural Pneumonia, and sometimes we have outbreaks of Chicken goat pox. An outbreak of foot and Mouth in Narok County affects sheep, goats, and pigs, but it mainly affects cows. The most common diseases affecting the cattle are East Coast fever, anthrax, and Brucella, which we encounter, and the main challenge they pose is that they result in the abortion of the cows. Also, malignant catarrhal fever affects the cows in this area, and they do not have treatment or a vaccine. Those are some of the diseases that affect the cows in this area. The sheep are infected with the Blue Tongue virus, and its Maasai name is Shamshami. From 2019 to 2020, it was hectic, resulting in many sheep deaths. There were thousands of sheep, but this was because there was no vaccine to protect them from being infected. Currently, there is a vaccine that protects sheep against it. Also, the farmers are affected by Rift Valley fever, PPR, which is Peste des petis ruminants. So, these are the diseases affecting the farmers in this area.

Researcher: So, with East Coast fever, we are researching the disease, identifying its characteristics, and managing and preventing it by the farmers in this area. So, according to your experience, what is the cause of this disease in the cattle?

Interviewee: Scientifically, it is caused by protozoa called Theileria parva. Sometimes, people refer to it as *Inaudible*, but all this depends on the country. Also, it is referred to as Corridor disease and has several names. But it is commonly known as East Coast Fever because it is found in Sub-Saharan Africa and spreads through the protozoa called Theileria parva. The vectors of this disease are the ticks and the animals that usually cause the disease. They are the carriers of the disease. So, it spreads through the ticks, which are in almost every place. So, the management of the ticks will depend on what you do to manage the ticks affecting your livestock and the type of acaricide you are using. Some farmers practice management of the ticks, but they only use one kind of acaricide to control the ticks for the whole year. Using only one type of acaricide poses the risk of resistance to the acaricides by the ticks. So, you will go to the farmers, and they will inform you that they spray their cows every five days and tell you that there are ticks that are affecting their livestock even after practising proper management and control. So, when you ask them the type of acaricide they use to manage the ticks, they usually name either of the two most common acaricides in this area, the Alamtics and the Amitracks. You will ask them how long they are using it, and they inform you that they use it daily, and this, in turn, leads to resistance of the ticks to the acaricides, which in turn exposes the cows to the disease.

Researcher: The other concern we have gotten from the farmers as we have been doing the fieldwork in Olulunga and Narosura wards is that we have realized that there is an issue with what the farmers view as the causes of the diseases. Have you ever encountered the misconceptions that the farmers believe are the cases of the disease, yet they are not?

Interviewee: Yes, this is very common with the farmers. Some even believe that the disease has unexplainable causes. You may ask the farmer what could have caused the cows to be infected with the disease, and they will tell you that they took their cows to another farmer’s farm and were infected with it. They usually attribute the causes of the diseases to the cows from other farmers, having their cows from their stocks and taking the animals to another area. So, as an animal health professional, I can understand this because if you had mixed the animals that had not been taken care of with the animals that have been taken care of properly, then they would have the ticks, and if they were exposed to East coast fever than they can transmit this disease to the healthy cows. The other misconception is that the livestock will be infected through curses. They typically say that their cows are cursed. Also, ECF causes inflammation in some parts of the body, and in severe cases, it results in blindness in animals. Some farmers cannot understand that a disease can have these effects on the animals, so they believe that they have gotten blind because they were cursed. Also, some farmers believe that it is caused after the cows have eaten poison. So, these are the major misconceptions that we get when we are working in the field.

Researcher: Which methods do the farmers use most in treating and managing the disease?

Interviewee: They use antibiotics for treatment, but most do not have the best medicine to treat the disease. In the field, many farmers use a specific medicine called Oxytetracycline. Terramycin 10% is prevalent, and people usually believe it treats all diseases. The issue is that the cows will develop drug resistance because the farmers use it to treat all the diseases. Still, when the cows are infected with East Coast fever, they must be treated with specific medicines, such as Bupafex and Buta Lex. But when using Oxytetracycline, you will have to use the long-acting types, the Oxytetracycline 20% and 30%. However, mainly in the treatment of ECF, we use Oxytetracycline 30% coupled with East Coast fever medicines, including Bupafex, Buta Lex, and Bupagon. So, this is the treatment. The spraying of cows with acaricides is a challenge because we not only need to use them, but we need to have them.

You mentioned that you saw a gap within the community. Currently, I organize community meetings where I have training and educate them on how to use the acaricides. I tell them they should not use the acaricides frequently and should alter their use over time. So, I usually have small meetings in the villages and sometimes go for free demonstrations and training. Training that time, I was working with some organizations, where we used to have sensitizations during the market days on these acaricides. So, with the acaricides, each has a different concentration that is used to kill and control the ticks. If they do not consistently use the same type, there will be no resistance. So, we advise the farmers to use the different types of acaricides to prevent the ticks from resisting the acaricides, a significant challenge affecting the farmers in the local areas. Many farmers embrace vaccination because it is cheaper and easier, and they are confident their livestock will not be infected. When the farmers have vaccinated their animals correctly, they may not be infected with any disease for five to six months. Even if the farmers' animals mix with those infected with ECF or the ticks have bitten, they will not contract the disease. So, many farmers are embracing vaccination for their livestock. There was a time when the county government did free vaccinations to some farmers across the county and even in the wards you mentioned.

Researcher 2: Were they vaccinating the cows against ECF?

Interviewee: Yes, they were. Some farmers were beneficiaries of the specific vaccines. However, very few farmers benefitted from this exercise. The Maasai are a pastoralist community and usually have a lot of livestock, cows to be precise, so it is hard to vaccinate all the cows. Also, the farmers are embracing the veterinary officers in the private sector, who call them to vaccinate their livestock. The challenge with vaccinations is the price, and there are no smaller doses of the medicine. The smallest dosage of the vaccine is to be used for fifty animals. So, the farmers with fewer animals will have a challenge in vaccinating their cows. After preparation, the vaccine is supposed to be used for six hours; after that time, the vaccine will not be as effective as it should be. All this is because it is a live attenuated vaccine. So, we experience challenges in the field. There are farmers with few cows who want them to be vaccinated, and they are only willing to pay thirty shillings per cow, and if they have ten of them, this is a total of three hundred shillings. So, you will rarely get the veterinary doctors going to Olengulo to vaccinate the cows at that price because the cost of transportation to that area is one thousand shillings. So, what I am doing is encouraging community-based teamwork. If the farmers have few cows in that area, I give them a notice that I will be vaccinating cows in a particular farmer, and I tell the village elders, and they will pass this information on to the villagers. So, they will pull their animals together and pool their resources together; thus, we will administer a general vaccination. This is cheaper, making it easy for farmers to access the vaccine at affordable prices.

Researcher: We have been doing our fieldwork in Olkiriaine, Oldonyorasha and Erupata. With the engagement of the farmers, they told us that they do not know if the vaccine exists. So, which areas do the farmers know about this vaccine?

Interviewee: Mostly the areas around towns and those that are not far from the towns. But in the areas you mentioned, some farmers have vaccinated their cows. We have specific families. So, the gap comes when we do not have the community sensitization. The problem comes with the training and the sensitization because if we have enough training and education on the importance of vaccination, there will be a breakthrough. Also, there is a challenge with the vaccination of the people, let alone the livestock, because we have mothers who give birth at home, and this also happens to the animals. However, the animals are most at risk because everybody in the community assumes the role of the veterinary doctor. The farmers also administer the medicine to their livestock, which becomes an issue. With vaccinations, there is a gap in the sensitization of people who do not have access to the vaccine. It is not expensive as it only costs thirty to thirty-five shillings per shot, but the main issue is the farmers' sensitization of the vaccine.

Researcher: So, is the price for ECF twenty-five to thirty-five shillings per cow?

Interviewee: Yes.

Researcher: How often do you conduct the training that you have mentioned?

Interviewee: Daily. I do the early morning vaccinations, and in the afternoon, I will discuss the other diseases with the farmers, not only ECF. As I have told you, there is a challenge in pasture management. Also, before I do the vaccination exercise, I make sure they understand what I am doing, and I have a meeting before I conduct the exercise. I also tell them to make sure that they have a meeting with the local authorities and that they have the barazas(community meetings) that they call me to train them.

Researcher: Have you experienced any challenges in the farmers embracing the use of the vaccine, the training and acquiring the vaccine?

Interviewee: There are a lot of challenges, especially with the vaccines. The verification exercise of the vaccines is usually hectic. I may be a very good person at work, but not all of us can do the job. There are some cases in which I cannot set up the vaccines, and there are times that there are failed vaccines that are administered to the livestock, and they are not effective. This then results in farmers mistrusting the veterinary officers. This is a challenge that we usually experience as veterinary doctors. This may result from using vaccines that may have expired, contaminated or compromised. The farmers are also complaining that the vaccine's price is high, which is why it is hard for them to embrace the use of the vaccine. I have always explained to them that if they have fifty cows and the vaccine is three thousand shillings the cows can be sold at the lowest price of twenty-five shillings currently. So, I usually ask them if they would instead let their cows die of ECF while they can just vaccinate their cows for three thousand shillings. I train them on the preference scale, which is part of the business. I usually tell them if they prefer losing the animal or paying three thousand shillings to vaccinate the cows. You may go to a particular homestead, and you will notice that they cannot pay for the vaccine; at this point, you can subsidize the vaccine. We can also do the vaccinations for free so that they can only purchase the vaccine for one thousand or five hundred shillings. If the vaccine is effective, they may be willing to vaccinate their cows the next time. Some of the farmers' misconceptions pose the challenge. Medicines Some farmers believe that they will make the meat of the cows taste bad and make their animals weak. Some believe it compromises the quality of the milk and the meat, and some say that it makes the cows susceptible to diseases. So, these are the challenges that we usually experience from the farmers.

Researcher: Thank you very much for the work that you are doing. I would like to ask if other partners in the animal health industry are educating the farmers in this county because it is very big and most of the population are pastoralists. So, are there some partners that you are working with to educate the farmers on the vaccine?

Interviewee: We have the county government that at times does the vaccination exercises and the public participation training. We also have the private companies that I had mentioned such as most of the chemical and acaricide companies doing the sensitizations in the market days. In this county, we have the designated market days in every area. So, these companies and private organizations usually go to the open-air markets on these particular days to train the farmers and carry out the sensitization program. We also have some societies that also do a lot of training exercises for the farmers.

Researcher: What could you recommend to be done so that the farmers can get the information on the vaccine and administer it we have talked to the farmers and they have told us that it costs one thousand shillings to one thousand two hundred shillings to administer to the cows and they have termed that as the major factor that prevents them from using the vaccine on their cows. What could you recommend to be done so as to address such an issue?

Interviewee: The farmers should be informed, and the only way this will be achieved is by having more training and sensitization sessions with the farmers. If I were asked to leave the treatment sector and was told to pursue something different, I would dwell on more sensitization and training the farmers. Once they have the information, they will have an option either to act on it or to be ignorant of the information. Currently, I can call the farmers ignorant because they do not have the information. This is a misfortune. So, to begin with, they should have more sensitization exercises from more teams such as yours, not necessarily the animals’ officers, as people can be trained to do so. I have several health volunteers in my community who have basic training on human health and this could also be applied to animal health. If we had them, they would inform the farmers that they should vaccinate their cows every six months, the available vaccines and their function. And once in a while, the experts would come to the meetings and provide the key information. But currently, if they take the majority of the youth that are in the community and train them to volunteer in animal health then this will be better. Maybe if we have the private sector investing more in the vaccines and also subsidizing them. The county government does free vaccinations but they cannot cover more than one million people in the county. So, if the population of the county is at one million people, then you will expect that there will be almost ten million livestock in this area because each family will have hundreds of livestock. So, the animal population is twice or thrice the human population in this area. So, I would encourage the private sector to take part in the vaccinations and to subsidise the vaccine. The vaccines should also be readily available in this area. Currently, the vaccine is not accessible in all areas and also it is not being sold in some agro vets. Like currently, it is very hard to access the foot and mouth vaccine because we are using the June batch that is ‘expired’ although we normally have a grace period of twenty-seven days. That will make the vaccine not available for those who want to purchase the vaccine. So, the availability and accessibility of the vaccine should be considered.

Researcher: Other than ECF are there any other vaccinations that the farmers have embraced?

Interviewee: Yes. Foot and Mouth We have the one done for the goats that almost every farmer does and it is called Caprivucks and this is the pleuropneumonia that the goats are usually infected with. So, it is almost administered by all the farmers. We also have the broad-spectrum vaccine which controls seven to eight animal diseases. So, there are some vaccines that the farmers are embracing their use on the livestock.

Researcher: With ECF, there are some areas where the disease is prevalent while there are some areas where the disease is not prevalent. So, in your opinion is the use of the vaccines in these areas similar in these areas?

Interviewee: With the prevalence and the vaccine usage we cannot have the proportional data comparison because in some areas such as Narosura mostly in Lelotengulo there are few cases of ECF but in Maasai Mara, and the Northern side of Narok there are higher cases of East Coast fever. However the usage of the vaccine in the northern region is 1% and they rarely do the vaccinations though the diseases are not prevalent, but in Mara, a good number of farmers vaccinate their cows. So, this all comes back to the information that the farmers have on the vaccine. The cows from this area may be dying and, in another region, they are surviving, the other challenge that the farmers have is that the animals that recover from the disease are usually carriers of the disease and these farmers do not know this.

Researcher: From the research I have done during the time that I have been here, I have learnt that there are different types of ticks. So, is there a specific type of tick that causes the East Coast fever to the cows?

Interviewee: I can’t say that it is very specific and we have the red ticks that are generally the carriers of the disease although this has not been scientifically proven. But generally, all the ticks expose the cows to East Coast fever. Also, the tall grass and poor management of grass can put the animals under exposure to East Coats fever because they are usually the harbours to the ticks.

Researcher: What could you recommend be done on the wildlife and livestock interactions we have heard that when the wildebeests mix with the cows that usually results in them being infected with East Coast fever.

Interviewee: For the wildebeests they cause Malignant Catarrhal Fever that is transmitted to the cows, not East Coast fever. Malignant Catterall fever does not have any cure or vaccine. When the animals have been infected with the disease, we have to kill the animal. This usually occurs when the cows and the wildebeests have interactions and this majorly occurs during calving season for the wildebeest. So, the wildebeest are the carriers and they are not affected by the disease but when the cows are infected with the disease then it results in a lot of damage. It is not much of a challenge but there are few cases of the infection but I cannot constitutively say that there are many cases of the disease. Because there is the privatization of the land that the farmers are fencing their farms. This has resulted in lowering the number of wild animals in this area, but there are still cases of East Coast fever. I should not disregard the wild animals do not cause ECF but I would not state it as a main factor.

Researcher: Do you think that there should be peer-to-peer communication for the promotion of the vaccines so that the use of the vaccines can be effective?

Interviewee: Yes, I do.

Researcher: Most of the Maasai men, when they are talking, usually talk about their livestock and the situations that they have, can this be a strategy that can be used to pass the information on the vaccine?

Interviewee: It will be very effective. Peer-to-peer communication is very important as when the farmers are having their normal conversations, they can talk about the vaccines. This will all lead to the information on the vaccines. So, when one farmer has the information and they have met somewhere as friends to hang out he can mention to them that they can use the vaccine before their cows are infected. This is very important in this area especially in this area because they love competition and always being the best. So, if a farmer hears that the other farmer has vaccinated their cows than they will also vaccinate their cows even before they are infected. If I go to one farmer to vaccinate their cows, I will get six or more clients at that time to also vaccinate their cows and this will also be contributed by peer pressure from the other farmers that will tell them that they have vaccinated their cows. This will make the farmers feel left out so they will have to vaccinate their cows.

Researcher: How often do you get the cases of ECF?

Interviewee: Quite often. They are among the many diseases that the farmers report in this area.

Researcher: Could you compare this in terms of the region ward and village?

Interviewee: Like I have said the Northern and the Western there are higher cases of the disease But in the Southern region there are fewer cases and I attribute this to the hot conditions in the region and the practices that they do and the lack of grass in that area. But in the cold regions, there are higher cases of the disease. There was a farmer who lost nine cows because of the disease and when I was called to examine the cows, he had already lost eight of them and the ninth cows I could not save it as it was already dying. It was in a place called Ndenek Ndavashi it is past Ngareta and it is toward Mau Forest near Njoreshumara.

Researcher: Have you had the cases that which the farmers usually manage the ticks and the disease without using the vaccine?

Interviewee: I have gotten such cases and I usually encourage this. I don’t have anything to do with the ticks because East Coast fever is one effect that the ticks have on the cows. They cause many other effects that the ticks usually cause to the cows so you do not control the ticks to prevent East Coast fever but to prevent other effects. I usually recommend the management of the ticks and also administering the vaccine because it is certain and it gives the assurance of the livestock’s survival. But with livestock management, the management of the ticks is done by the farmers not only to prevent ECF but also for other effects.

Researcher: Are there any differences in the demographic and the characteristics of the farmers? Some farmers rear the large scale of the cows and they have the hybrid breed of the cows while there are some that keep the cows on a small scale. Is there a difference in these types of farmers embracing the vaccine?

Interviewee: The farmers that are practising commercial farming have embraced the vaccine. The Maasai do not rear the animals as a source of wealth but it is because it is a privilege to have them. You will find that some farmers have had a cow that is ten to fifteen years old and the lifespan of a cow is between twenty to twenty-five years. They keep the cows for this long time and it is not a sign of wealth. But the society has changed because in the past they usually reared the cows to show royalty. After all, you would find if a man did not have a certain number of cows they were not respected. The culture has changed and they are now embracing commercial farming thus they embrace the use of vaccines. They vaccinate their cows because they may die of the disease and in turn, this will result in losses. So, to prevent the losses they will have to vaccinate the cows. But with the farmers who are not financially stable, it is a challenge for them as I had explained earlier. It will be very hard for the veterinary officers to vaccinate the few come because of several factors including the packaging of the vaccine because you will not be able to buy the vaccine that will be used to vaccinate fewer cows. It is either one hundred or fifty cows. So, if the farmers are willing to vaccinate their cows and they only want to vaccinate ten of their cows then it is a challenge to the farmers. The farmers who know the value of the vaccine can decide to pay the price for thro nr hundred cows and the extra can be disposed. So, these are some of the dynamics as the farmers will not see the need to vaccinate their cows because it will be very expensive and they will have to buy the vaccine that is enough for one hundred cows yet they only have ten. So, in this case, I do the community sensitization and I inform them to pool their funds and ensure the farmers from the same locality bring all their cows together so that they can reach the one hundred cows so that I can vaccinate all their cows.

Researcher: What impact do the farmers get after their cows have been infected with ECF?

Interviewee: With economic empowerment, if my cow is vaccinated, they will not be infected with ECF and this means that I have a source of income. When I sell the cow, I can take my children to school. The cows can also produce milk, which is also another source of income to do other activities. I have mentioned a case of a farmer who lost nine cows because of the disease, this has affected him and his family as these changes the plans that they had for the next three months. Maybe out of the nine cows, seven were dairy cows and a litre of milk costs thirty shillings to forty shillings. If they were getting ten litres of milk daily and say they sold it at thirty-five shillings per litre then they earn three hundred and fifty shillings daily. This means that they can buy bread, two kilograms of sugar or two kilograms of maize flour. So, with the vaccinations, they improve the standards of living for the farmers by increasing the sources of income for the people, bringing sustainability to the family and easing the cases of conflict. We have some times that the family conflicts and feuds after the cows have died of a disease that could have been prevented resulting in divorce. Does it have social and economic impacts? But if they have vaccinated their cows, this brings harmony in the family, increased standards of living and sources of income and the level of education. Currently, the families can pay for their children’s education which in turn brings a change in the community.

Researcher: What can be done to address the issues the Maasai face in rearing their cows as they have complained of the drought, diseases, theft in some areas and the wildlife and animal conflict?

Interviewee: The farmers need to have the information. Because to change the community it requires few numbers of people. We have to discuss the behaviour changes. There have been some climate changes in this area. We usually had a lot of rain that made the grass grow but currently, the amount of rain has reduced. We all know that weather changes cut across everybody in a particular area and the farmers are living as if we are in the past. We need to pull all our resources together and put our efforts together. The government, the people in the private sector and those in the Non-Governmental Organizations should also work to teach the villagers. They should begin by teaching them and the best method of addressing this issue in Maasai land is by the farmers getting this information. It will begin by training all the members of the community so they may adjust to the behaviour changes. You cannot tell an old farmer to sell their cows and begin livestock farming and they accept. You can only manage to change their behaviours on how they have reared their cows and this all starts with what the veterinary doctor will do when they have gone to their home. So, this is a must cut all through the challenges as we all know that the Maasai usually lived with wild animals from the past and what may have changed is because they have fenced their farms and the human activities that take place in this area. So, as custodians of nature, they should identify new ways of staying with the wild animals because they cannot be eradicated from this area as they are part of our heritage. With disease management, they should know the types of diseases that affect the livestock in Narok, how they can be managed and who they should go to when their cows are infected with the diseases. We have emergency contacts for other issues but we do not have an emergency contact for emergency issues. If we have a case of East Coast fever, there could be a contact that they can call so that they are attended to thus saving time. We have to integrate the technology, information and the community and come up with a single package that is harmonized to help the people to achieve what they want.

Researcher 2: In your opinion, are the farmers able to tell the difference between ECF, MCF and other diseases?

Interviewee: Not all the farmers can do so. We have some farmers who can do so because we have the distinct physical symptoms of ECF. But I cannot give a general answer that they can or they cannot but some farmers can. It is also part of the training that we are giving and I train them on how they can make the distinctions because there are some medicines that you can administer to the cows and if they are not used to treat that disease then it can cause effects to the cows.

Researcher 2: You have mentioned that they mostly use Oxytetracycline to treat their cows. The most used one is the Terramycin 10% while you have mentioned that the most effective one is Terramycin 30%. Why do they mostly use the former?

Interviewee: It is because it is cheaper. Terramysin 30% is sold at nine hundred shillings to one thousand shillings while Terramycin 10% goes for one hundred shillings or two hundred shillings. So, the farmers can easily buy this.

Researcher 2: When we were in the field many of them were telling us that they use the Terramysin 10% and when it is not effective that is the time, they buy the Terramycin 30%.

Interviewee: They usually go for cheap medicines but in the end, it becomes expensive to them. So, they mostly buy the Terramysin 10% because it is cheap.

Researcher 2: In your own opinion, do you feel the adoption of the ECF vaccine is because of the cost of the vaccine or there could be other factors?

Interviewee: There could be other factors as I have mentioned. It could be a lack of information because some farmers do not know that the vaccines exist. Others have misconceptions about the vaccine and they believe that when they vaccinate their cows, they become weaker and susceptible to diseases. So, these are some of the factors that make the farmers not use the vaccines.

Researcher 2: What about the fact that some of the farmers do not believe that ECF exists?

Interviewee: This is also an issue because some farmers do not believe that ECF exists. Unlike Foot and Mouth disease, they believe that ECF is not an issue because they may treat their cows with Terramycin 10% and they get cured of the disease. They may use a lot of medicines to treat the cows and the cows get cured of the disease so they feel that it is not that dangerous It is just like humans, where very few people go to the hospital when they have the flu even if it persists. It may be a serious disease but since they have signs similar to the flu then many of people usually ignore it.

Researcher 2: So, this means that some of the farmers know that they can manage the disease that is why they cannot vaccinate their cows?

Interviewee: Yes.

Researcher 2: From what you have observed what is the most common Maasai practice that the farmers do when their cows are infected with any disease?

Interviewee: Mainly before the introduction of treatment using drugs and chemicals, the Maasai would practice the conventional treatments. They would administer herbal medicine from the medicinal plants would rub them on the cows when they have the inflammations. They would also make a concoction using the herbal plants and administer it to the cows and this was the first response. The second response was that they would go to the veterinary doctors and tell them the symptoms of the infected cow who would then give them medicine that they would administer to the cows. They may also do the simple management and treatment practice; they are told to buy over-the-counter medicines such as Oxytetracycline and they administer 15ML or 20ML to the cows. But with those that cared they usually call the veterinary doctors to examine and treat the cows. If the disease is persistent, those who do not call the veterinary doctor, usually curl the cows and dispose of them.

Researcher: On the point that they can get the medicines in the agro vets and they are using the OxytetracyclineOxytetracycline frequently, can antimicrobial resistance be an issue?

Interviewee: It is already an issue and we are getting many cases. The most disappointing thing is that you will get the farmers using the acute medicines that I would not encourage and they are using Dexamethasone which is one of the medicines I usually am against. Because if the medicine is not prescribed by a veterinary doctor or a qualified animal officer, I cannot encourage it. That medicine is a steroid and we all know the effects the steroids have on the animals. So, this is already an issue and you can know this through Terramysin 10% that you were talking about. You have had conversations with the farmers and currently, the medicine is not effective unless it is used on cows that have never been administered the medicine.

Researcher 2: How do the farmers know the medicines that they are supposed to administer to the cows? Is it because of the rogue veterinary officers?

Interviewee; I cannot call them rogue veterinary officers but sometimes the farmers are arrogant. If they have ever called the veterinary doctors and when they have come, they see they have used Dexamethasone when their cows are inflamed and they also have pain. The first thing they ask you is when you have reached that area, they will sample all your medicines. The issue we have with the Maasai is that they all treat their cows personally. So, they will ask you the names of the medicines and they will keenly listen to the names and take note of them so the next time that their cows are infected then they go to the agrovets and ask for the medicines. The veterinary doctors in the agro vets are not usually prohibited from selling the medicines that the veterinary doctors have used to treat their cows. I usually discourage this because I know the effects and because it is a business, they will have to sell the medicine. The farmers are usually very cheeky but they buy the medicines without knowing the effects that they may have.

Researcher 2: The farmer also claims that the sheep are infected with Oltikana. Could you describe this?

Interviewee: I cannot say that it has been fully classified but it is caused by the feeding management practices. They want to term it as ECF for the sheep but it does not exist. We have the Nairobi Sheep Disease which is termed as Oltikana in this area and it is rampant in this area. They have the same symptoms as ECF in cows and the carriers of the disease are also the ticks.

Researcher 2: Does it have the same treatment?

Interviewee: Yes. The major issue is we do not have its vaccine yet. But when the farmers use the broad-spectrum vaccine, we usually control a large number of sheep and goat diseases. So, with the farmers who practice proper tick management and some who have been vaccinated using the broad-spectrum vaccine then they will have fewer cases of the disease. However, with the signs and symptoms of the disease, are similar to ECF.

Researcher: With the changing climatic conditions changing in Kenya and the whole world do you think that pastoralism will be effective and sustainable to the farmers?

Interviewee: Leave alone the climate changes. Human activities, increased population and privatization of land will affect this. In the past, we used to have community land where you could graze the livestock. We used to have farmers from Ntulele grazing past Oldonyorasha. At that time, you could graze at any place and you could camp in any area semipermanent but currently, you cannot. This shows the effects privatization of land has had on pastoralisation. With climate change, there are unpredictable climate patterns. I was having a conversation with members of the community today and they were telling me about the climate change in this area. They told me that in the past, they usually had up to six months of rain. Currently, they only get two months of rain. So, we do not have enough grass and water for the animals so this makes the farmers think of other alternatives. This is something that we should also teach the people of this community. We should tell them that there are other alternatives to livestock rearing because there are some factors that we cannot control. Because we do not matter how many trees we plant, the carbon IV oxide that we are not supposed to omit, controlling the ozone emitted and all the scientific factors, we cannot fully combat climate change. We can do all that we can, but since we cannot control what the climate has in store, we can change what we do with the conditions. So, this is a discussion that we should be prepared for. One of my friends says that climate change has come prepared for a nuclear war and we are using arrows eventually we will lose the battle.

Researcher: Thank you very much I am done with the interview.

[END ]
