# Supplementary material for: Beyond the jab: Unravelling the complexities of vaccine adoption for East Coast Fever in rural Kenya
Source: PLoS One. 2025 Jan 28;20(1):e0315906. doi: 10.1371/journal.pone.0315906 (PMC11774369; doi:10.1371/journal.pone.0315906)
Supplement: S1 Dataset — (ZIP) [file pone.0315906.s001.zip › Supporting information (R)/KIIs/230801_0944 ELDER .docx]

**KEY INFORMANT INTERVIEW - VILLAGE ELDER**

R: You will start by telling me, here in your village, which diseases primarily affect cattle?

I: Here in our village, we have Malaria Malaria which affects the eyes of cattle, and also, there is a type of Malaria in which when you slaughter a cow, the meat will be greenish like this grass. Malaria is here, and Entorobo, which usually infects cattle when it rains. Wild animals transmit these diseases. For instance, when elephants come to this area, we will face that problem to some extent, but we will not treat cattle often when elephants do not come to this area. The diseases are Malaria and Entorobo (Sleeping sickness) in cattle. Also, cattle can lose weight because of drought; cattle will become weak, and the hooves will be swollen, as will sheep. Those are the diseases we have in this area.

R: On Malaria, you have said that when it infects cattle here in this village, how do farmers (you included) seek treatment?

I: When cattle are infected with Malaria, I will go to the chemist and buy Terramycin, and I will inject normally; the two syringes, or even three, in some cases, I will inject four syringes, and then I will observe if it heals. If it will not heal, the next option is to sell.

R: What signs and symptoms will help a farmer know that cattle are infected with Malaria?

I: None. It is just us that we will see if it has Malaria; there will be some swelling on the sides of the neck and also on the lower abdomen; that is what we will see, and we will know it has Malaria.

R: What will the appearance of the cow be? For example, as you have said, the meat can be greenish.

I: The fur on the hide will be vertical,

R: The fur of cattle?

I: Yes.

R: What of the eyes and mouth?

I: The eyes will shed tears.

R: In your opinion, what causes this Malaria in cattle? How would you describe the perceptions and beliefs of farmers in the village regarding the causes of Malaria?

I: Maybe it is water. I think it is water. There is also another disease in this area, Olkirobi, that affects the mouth of cattle. That is the most prevalent disease here.

R: Now that you are an elder in the village and have kept cattle for many years, was this disease in the past, or did it start recently?

I: Recently, we have seen the outbreaks have reduced significantly. There was a time in the past when we would often slaughter so many cattle in this area.

R: In the past, what did you perceive as the Maasai was the cause of East Coast Fever?

I: It is just that the remedy there was to treat with Terramycin, and there was nothing else we could do.

R: Was there any aspect in your traditions that you associated as the cause of Oltikana?

I: No, we thought it was a disease from water and grass. In some instances, when cattle would migrate to a far place, it would be infected by that disease.

R: When comparing this village with other areas, where are Malaria outbreaks high?

I: It is these areas towards the plains, that malaria.

R: Which places are those?

I: The places of Oloonkerin, and towards Lemek, they have Malaria, even now I have some of my cattle in Oloonkerin, which have been infected with Malaria.

R: When were they infected with Malaria?

I: They are infected and will have something like swollen lymph on the neck region.

R: Which drug have you been treating it with?

I: Terramycin

R: Have they healed?

I: There are some which have already healed.

R: Which type of cattle were they?

I: Just like this type of cattle here in the homestead.

R: Were they bulls or cows?

I: Not bulls, they are female cows.

R: What is the size of the cows? Big ones or calves?

I: They are big, they are not that young, they have not yet given birth.

R: How do farmers prevent ECF here?

I: There is none.

R: For example, now for cattle not to be infected with Olkirobi, there is a vaccine that can be injected,

I: There is none, the only drug we will inject is Terramycin, there is no other drug. If one can afford those expensive ones for 2,000 you can inject them into cattle. And its concentration is only ten per cent.

R: How effective is that when you inject cattle?

**I:** It is very effective; it will heal the cattle completely.

R: Are you familiar with other village elders around?

I: I am the village elder here.

R: How do farmers disseminate information to you- the village elders when they get cases of East Coast Fever, and they can't access treatment?

I: I will get information, and they will ask me how will seek treatment for this cattle because I have seen, it has been infected, and I have injected it in vain. And there is no other option, if you are treated with all these drugs and it will not respond, you will have to sell it.

R: Is there a way that you as the village elder refer them for help elsewhere?

I: I will tell them you can go to some place and get the help from the government. We will see and treat it or bring a doctor to come and treat it.

R: Is there any help that you have ever got from the government in treatment of East Coast Fever?

I: I think we will just go and bring a person, who will come and treat. But other things, there is none. There is only one time in the recent past that they came to vaccinate cattle against Olkirobi.

R: Have you used any vaccine before in prevention of Malaria?

I: It is only that vaccine for Olkirobi that we have used some other time but not recently.

R: Where is that vaccine administered from?

I: In one homestead; all cattle will be assembled there, and they will be vaccinated.

R: On the cattle, on which part will it be injected?

I: It will be injected on the tail.

R: You have never used for Malaria?

I: We have never used any for Malaria.

R: How many types of Malaria do you know?

I: There are two types of Malaria. One that the eyes will shed tears, and the other that there will be no tears, you will see in the appearance that cattle has Malaria. The one for eyes is the one that is very severe.

R: How and why is it severe?

I: It will kill cattle very fast.

R: In a span of how many days?

I: In three or two days, it will die.

R: What of the other one?

I: The other one is less severe because you will inject cattle, and it will respond quickly.

R: Have you observed any specific challenges that farmers face in accessing veterinary services for cattle? For example, when your cattle are sick, what challenges do you face?

I: I can go to Lulung'a, if I don't get one, I will just come and treat the cattle with Terramycin. There is nothing else.

R: Going to Lulung'a, how is the transport system there? Are the roads, okay?

I: No, the motorcycle is expensive, going there one way from here is 300, and the same coming back, so the total is 600 and you have not eaten anything.

R: What is the price of Terramycin and other drugs?

I: You know Terramycin has become so expensive these days, like the small bottle for ten per cent concentration, it is about 500.

R: On the vaccine, have you heard of any vaccine for Malaria?

I: No, I have not heard of it. There is no day we have got a remedy to the Malaria in this area.

R: So, it is not available at all?

I: It is not available at all.

R: And when you ask the veterinary doctor or when you go to Ololulung'a at the market?

I: I will go to the market, pay for the cattle to be treated and I will go.

R: The doctor has never told you if you can inject any vaccine into cattle to prevent Malaria?

I: No, there is no time completely.

R: Is there a time when the area chief has communicated to the village elders here on how to educate farmers on the treatment and prevention of cattle diseases?

I: Yes, there is a time when we have a meeting together, we can have the meeting in Lulung'a and check on the affairs of cattle. We discussed but they have never come to see if they will vaccinate cattle.

R: When was that time when you had the meeting?

I: It was almost ten months ago.

R: Was it this year or the other year?

I: It was last year.

R: What were you trained about?

I: We were trained on the signs and symptoms that you will observe on cattle, and when to call the veterinary to come and check cattle. But they have never come yet to check cattle in our homesteads.

R: Who were the other people in that meeting?

I: They were some officers from Olulunga.

R: Who were taken from this village?

I: We are many here, almost all the elders of villages.

R: After the meeting, did they instruct you to come to disseminate the information to the residents in the village?

I: We came and talked and told farmers to do this and this on cattle, you know Maasais love cattle immensely.

R: After the meeting, did you come to share the information with your village constituents here?

I: Yes I came and we talked, there are these drugs that you can use when you notice certain symptoms on cattle and there are others which you have to call the doctor, not just treat on your own.

R: So, you talked to the other men?

I: Yes.

R: What communication channels do you use to pass information about livestock health?

I: Yes, we have, but the drought has been a problem for us. We only have a little grass left which has made us not have the forums to pass information to farmers.

R: Have you heard from veterinary agro vets or veterinary officers if they have vaccines for Malaria?

I: I have not gone to a veterinary officer, I can go to a certain doctor, we can go there, there are these young men that we use to check cattle, we will bring the drugs from...

R: Would you want the vaccine for Malaria to be made available to this village?

I: If we can get, we will be very happy, because cattle get infections of Malaria.

R: What price would you be willing to buy the vaccine?

I: That Terramycin?

R: No, the vaccine to prevent Malaria.

I: I have not yet bought that one.

R: For example, if it is retailing at 1000 now, will farmers be willing to buy it?

I: Yes, if you are protecting your cattle, you will just buy. Even if it will be sold at 2000, we will just buy. Because your cattle will not die. I like it so much. Even recently, there is a bull of mine that became sick, I went to look for a doctor and I could not find one, I went to buy at the agro vet the one for 2000 and it got healed. The bull that you saw here a few minutes ago.

R: Based on your experience and knowledge, what recommendations or strategies would you suggest to further promote farmers' health-seeking behaviour and increase vaccine adoption for East Coast Fever in the community?

I: If the government has the capacity, they should bring this remedy for the vaccine of Malaria together with that of Olkirobi because the government is well able to do that work. That is the request of help I would make.

R: In this other diseases like Entorobo (Sleeping sickness) that you have said, what recommendations can you give so that you can seek veterinary services and drugs easily for them?

I: If someone knows the vaccine for Entorobo, they can come to inject for us because it is not common to find cattle that have Entorobo (Sleeping sickness)in this area. When other cattle migrate to this area, those are the ones that will come with Entorobo (Sleeping sickness) and it will be treated. Malaria is the challenge and Olkirobi.

R: You were saying Malaria is not as prevalent today as it was in the past?

I: Yes, it is not as prevalent as before, but you will find some infections. They get infected.

R: What do you do now so that the prevalence is not high?

I: Maybe cattle will not migrate often at that time.

R: There are vaccines, for example, the one that prevents Orkipei in goats. Have you had such done here in your village?

I: Yes, there is—Orkipei for cattle and goats.

R: Have you had any officers who vaccinated your cattle to prevent it?

I: There is none,

R: Even goats?

I: There is none. When you notice a goat has Orkipei, you will just go to the agro vet, buy drugs, and we will come to inject the animal. Or we can bring a doctor like you, and they will come to treat us, and we will pay them.

R: So, people do not visit the agrovet as a group to seek vaccination?

I: You will just go with your goats; you will go with your goats, count and give the number there, even cattle.

R: So, no people have ever come to vaccinate for you here?

I: There is none. It only happened once when they came to vaccinate against Olkirobi, and they did it at another homestead over there, at the Chief's home. They were only vaccinated then, and they have never returned.

R: Your cattle were also vaccinated?

I: Yes, but it was in the past. It is almost four years.

R: Were your cattle alone vaccinated, or were there others?

I: They were vaccinated for all homesteads in this area.

R: Who planned for those people to vaccinate all the cattle? Was it the village elder, or was it the Chief?

I: It is the Chief of the location, who is called Chief, who went to request Lulung'a for our cattle to be vaccinated, together with the village elders.

R: You said there was that training that you were given?

I: Yes,

R: When was it?

I: It has been one year since.

R: It's almost a year ago?

I: Yes. We have not yet gotten any training recently.

R: You said the training was about?

I: Regarding the issue of Malaria in cattle, Olkirobi, we were just trained on cattle-keeping practices.

R: Who informed you there was that meeting, or how did you know about the training?

I: The Chief made a phone call to us, and we went to Ololulunga.

R: Okay, I am done with the questions.

R: There is a vaccine that prevents seven diseases in goats. Have you ever used it?

I: The one that prevents seven diseases. We vaccinate goats and sheep sometimes, but we don't vaccinate that in cattle. It is only goats.

R: How often do you vaccinate that in goats and sheep?

I: Like twice.

R: Like now, what was the last time you vaccinated them?

I: I was vaccinated just recently, around February there. I am almost to vaccinate them again because when this maize is harvested, cattle, sheep and goats from far away will be migrated to come here.

R: Since you vaccinated them, have they been sick?

I: They were prevented from diseases. But you will go and buy it with your own money.

R: You said that water is one of the causes of East Coast Fever. Where do you take your cattle to drink water?

I: There is a time when the river downhill will be flowing with water, and there is a time when it will dry like it is now. There will be stagnant dirty waters in some parts along the river, and when cattle drink that, they will be infected.

R: How do you deal with that, or where do you get water for your cattle, so they don't drink it?

I: If this river here dries completely, we will go to the other permanent river, Ewaso Ngiro River. Now they drink here, it has some little water.

R: What if grass finishes here? Where do you migrate your cattle?

I: We will migrate them to the sides of Oloonkerin as long as you find a farm with pasture that you will lease.

R: Alright then.
