# Supplementary material for: Beyond the jab: Unravelling the complexities of vaccine adoption for East Coast Fever in rural Kenya
Source: PLoS One. 2025 Jan 28;20(1):e0315906. doi: 10.1371/journal.pone.0315906 (PMC11774369; doi:10.1371/journal.pone.0315906)
Supplement: S1 Dataset — (ZIP) [file pone.0315906.s001.zip › Supporting information (R)/KIIs/04072023-1114.docx]

KII-Vet-Agro Vet Owner

Researcher: For how long have you been in the field of treating animals?

R: 8 years.

Researcher: In the past eight years, have you observed any information the farmers missed about the causes of East Coast Fever?

R 1: East Coast Fever has a higher mortality rate and clinical signs than other diseases. This can lead the farmer to treat another disease instead of ECF.

Researcher: Have you observed any instances where a farer comes here with explanations claiming to be ECF, but in a real sense, it is another disease?

R 1: I am a veterinary officer working together with nine ECF vaccination teams.

Researcher: What are the main barriers or concerns you have regarding the adoption of the Oltikana vaccine?

R 1: Its cost.

R 2: It's fear.

Researcher: When farmers come here, are they sure the cattle suffer from ECF?

R 1: No, they are not because, currently, you will not satisfy a farmer until you go to their farm. Otherwise, just observation from the farmer cannot confirm if it is ECF.

Researcher: Do all your farmers request your services to go and examine their cattle?

R 1: It depends on the kind of farmer. You know you can get some very ignorant farmers. Some understand, and others decide to treat on their own. So, it is a reasonable thing for a vet to follow up.

Researcher: Can you tell us what you do in the team of veterinary officers?

R 1: Currently, we have the vaccine of Muguga KALRO, so by the end of the day, we have the vaccines that are availed at the farmer's request.

R 2: Regarding treatment, I use antibiotics, such as Butalex.

Researcher: Is there an incidence where the farmers requested the vaccine, and you were able to provide it to them?

R 1: For the last two weeks, I have been doing vaccinations at places a bit far from here. We arealready doing vaccinationsyonn the ground.

Researcher: What prices do you charge for the vaccines?

R 1: It depends on the transport criteria and the movement logistics. For instance, we have a different price if a farmer is in Eldoret. So, it also depends on the location.

Researcher: Let's say I am a farmer with 50 cattle, and I come to your shop and request vaccination services; how will you charge me?

R 1: One does go for 40 cattle, but we have introduced a dose of 10 cattle. So, now it is available. We charge 1000 shillings per animal because we have deworming, ear tagging, and vaccine injections.

Researcher: Are the farmers advocating for it or spreading the message to other farmers to adopt it?

R 1: They accept vaccinations, but the challenge is that they are expensive. So, not every farmer can adopt it; otherwise, those still willing can access it.

Researcher: I understand that there are areas where ECF is prevalent. How do you compare the administration of the vaccines in this area?

R 1: Vaccinations are going on in these areas. Education is power, and the preference for vaccination has become high. In regions like Maasai Mara & Talek, there is an outbreak of ECF; otherwise, many vaccinations are going on in that area. You know, areas like Olulunga and Ewaso Nyiro and in Ecozone 1. Ecozone 3&4 there are range of lands which have a lot of cases of ECF. In places like Olulunga, there are few cases because of the farming system.

Researcher: Having worked with Sidai FM, have you partnered with them to pass information about vaccination to the farmers?

R: Yes. Every Friday, we have a program with them. We are penetrating the ground, not like any other vaccines. Like FMD, CBPP is already rampant, and people vaccinate against it in thousands, but with ECF, it is very low because of education awareness. The extensions that the SVS provide are very few.

Researcher: Is the county government doing anything to address that challenge?

R: No. We still haven't reached that point.

Researcher: What time is the Friday show?

R: It does not happen every Friday but only on the days that the information is to be passed.

Researcher: Aside from that, are there programs that advertise and sensitize about the vaccines?

R: Yes, during the normal vaccination for FMD, we also advertise for the ECF vaccine.

Researcher: How often do you usually vaccinate against Foot and Mouth?

R 1: It is between May and August because of the migrations and when there is a harvest of maize and wheat.

Researcher: When you compare the dry and rainy seasons, what time do the farmers use the vaccine the most?

R 1: During the dry season, there is a lot of movement.

Researcher: What can you recommend about creating more awareness of the ECF campaigns?

R 1: If we can get more extension services in terms of facilities and transport, it can help.

Researcher: In your practice in this industry, what challenges or impacts have you seen regarding the treatment given to the animals?

R 1: Vaccinating animals already infected with the disease, thus becoming contagious.

Researcher: Which disease is a threat to the animals in this region?

R 1: Currently, we have contagious bovine, BPP, and PPR.

Researcher: Do you currently have the medicine for this disease?

R 1: We have 30% Terramycin, butalex, and multivitamin.

Researcher: Have you had feedback from the farmers to whom you have administered the vaccine?

R 1: Once we vaccinate the animals, we leave them there.

Researcher: What is the age of the cows that you vaccinate?

R 1: Calves of 2 months to 5 months.

Researcher: Is the antimicrobial effect rampant here?

R 1: Yes, it is rampant. Here in Narok, we have a long way to go. When I was a young guy, we used to know about Terramycin, but I think we need a lot of education on this. You cannot convince an old man on what to inject, like in other counties where they have to call a vet to treat their livestock.

Researcher: Is it that in Narok, they have a higher experience in cattle keeping compared to other counties?

R 1: We were born with animals, which are our security.

Researcher: At what time do they reach your vets?

R 1: At the acute stage. So, even saving an animal becomes a challenge. Maybe the farmer has injected 123, and the animal has not responded; you will see a farmer coming in now, probably saying he needs Terramycin. We sell it to them, and they go.

Researcher: You talked about farmers in Ntulele who adopted the vaccine. What are the characteristics of these farmers? Are they having money or many animals, or are they young?

R 1: As the world evolves, people become advanced with the technology that advances with technology, improving production. It is about empowerment and education. If a cow is worth 70000 shillings and a farmer has 50 cows, that is worth 3.5 million shillings, the farmers view vaccination as the better option.

Researcher: All those that you have gone to vaccinate, are they educated?

R 1: They are educated, and many are political leaders, people with great exposure. They have gone to different counties and seen what vaccination is doing. Probably, the death rate is high, and they feel the pain of it, so they decide to adopt it.

Researcher: For the farmers in the middle class, do you advocate for them to use the ECF vaccination?

R 1: Yes, ECF is a manageable disease, and you can control it. We advise a schedule scheme to farmers. It is not always about vaccinations. You don't need drugs or antibiotics; you just need proper management of ticks. You can use Ivermectin, acaricides, and proper deworming to control ECF.

Researcher: What would you say about their frequency of spraying?

R 1: It depends on the financial capability of the farmer. They spray every Tuesday after every two weeks.

Researcher: When comparing cattle, goat, and sheep diseases, which affect the farmers the most?

R 1: Sheep and goats are affected more in this area.

Researcher: So, in this area, is there was an intervention, they would probably prefer sheep and goats.

R 1: To where we are headed, there is a probability that they would go for the cattle because of the carrying capacity.

Researcher: There is also a disease we have heard of blue tongue. Is that the current outbreak? We have been to Ntitini villages, and they talk about it a lot.

R 1: It's an insect that causes the disease, which occasionally occurs during the rainy season when there is plenty of grass. It is a viral disease.

Researcher: I have heard of endemic stability. Does ECF in Mara affect cattle or those moving from this area to that area?

R 1: ECF is a disease that affects all animals. Even if the animals come from Loita to Olulunga, this does not change anything. We have exotic and indigenous animals. If they are moved to this place, to Maasai Mara, the immune system of these animals will be very low, so they will not be resistant to the disease. They can even be killed in 2-3 days. On the other hand, they are resistant, and diseases cannot kill them.

**END**
