# Supplementary material for: Beyond the jab: Unravelling the complexities of vaccine adoption for East Coast Fever in rural Kenya
Source: PLoS One. 2025 Jan 28;20(1):e0315906. doi: 10.1371/journal.pone.0315906 (PMC11774369; doi:10.1371/journal.pone.0315906)
Supplement: S1 Dataset — (ZIP) [file pone.0315906.s001.zip › Supporting information (R)/IDI/230627-1023.docx]

**IDI MAN**

**Researcher:** I will ask you questions about Oltikana (ECF) and the other diseases your cows are infected with. So, which diseases are your cows usually infected with?

**Mzee 1:** The cases of Oltikana reduced because many people spray their cows and manage the ticks; we were informed that the ticks cause Oltikana, so this made the people increase the frequency of spraying the cows, thus the cases of Oltikana have reduced. Some small insects called Salt Lake are usually in the areas that sting the cows, and if you do not wash the cows, they will be infected with Oltikana.

**Researcher:** Other than Oltikana, are there other diseases that the cows are usually infected within this area?

**Mzee 1:** The diseases that wildebeests cause usually affect the cows in this area, and since we have not fenced the farms and they are not prevented from coming to this area, the cows are usually infected with the diseases from them. This results in the death of the cows. If you go to the slaughterhouse that is nearby, when you ask about the disease that has resulted in the cows being taken to it, the majority is because of Engati (Malignant Catarrhal fever (MCF). It causes a lot of deaths yearly. So, this disease affects us.

**Researcher:** What is the name of this disease caused by the wildebeests?

**Mzee 1:** Engati.

**Researcher:** So, Engati is usually common in this area?

**Mzee 1:** Yes.

**Researcher:** Is there any other disease the cows are usually infected with?

**Mzee 2:** Olkipei (CCPP).

**Researcher:** Which animals are infected with this disease?

**Mzee 2:** The goats.

**Researcher:** What about the disease the sheep are usually infected with?

**Mzee 3:** A disease affects the mouths of the sheep, and it affected us in 2020 during the pandemic. It resulted in the deaths of very many cows.

**Researcher:** Those are their diseases. Are there any other diseases you have not mentioned affecting the farmers in this area?

**Mzee 3:** There is also a disease called Emburo that affects the cows mostly.

**Researcher:** Do all three diseases have the medicines and are they accessible?

**Mzee 3:** With this disease that the sheep are usually infected with, it is tough to find the medication, and as of now, we have never had the medicine.

**Researcher:** Does the disease usually affect the farmers once a year, in different seasons, or are sheep infected with it multiple times in a year?

**Mzee 3:**  The sheep are infected with it once a year. But since then, the number of disease cases has reduced, and we have not experienced it as such in this area.

**Mzee 1:** The goats are infected with PPR, which causes the sheep to produce mucus. It also gives the sheep a dry skin coat and diarrhea. If you do not vaccinate the goats after every three months, they will be infected. If they are infected when you have not vaccinated the goats, they cannot be cured. You are supposed to vaccinate the sheep to prevent them from contracting the disease, but after they are infected with the disease and you have not vaccinated them then, they cannot be cured even when you call the veterinary doctors to treat them,

**Mzee 2:** They are also infected with Kububo.

**Mzee 1:** There is also pneumonia that affects the cows, and it is usually from the *Saltlake,* which results in the cows being infected with it. This makes the cows weak and immobile.

**Researcher:** Comparing PPR to Oltikana, which spreads quickly among the livestock?

**Mzee 1:** PPR is a deadly disease and spreads quickly.

**Researcher:** Which disease among the two is the hardest to treat?

**Mzee 1:** When the cows are infected with Oltikana, they die one cow at a time, but with PPR, they die all at once.

**Mzee 3:** It usually has a window period.

**Researcher:** How does it have a window period?

**Mzee 3:** *Inaudible.*

**Mzee 1:** The cows are infected with Foot and Mouth, which affects us. It spreads quickly among the cows, similar to PPR.

**Mzee 3:** When the cows graze and follow the trail of the infected cows that will be infected with the disease. They will also be infected with it if they drink from the same water point.

**Mzee 1:** If the cows also sniff the urine of the infected cows, they will also be infected with Foot and Mouth.

**Researcher:** So, when the cows are infected with foot and mouth, can they easily be spread to other cows?

**Mzee 1:** Yes.

**Researcher:** Is this also the same for Oltikana?

**Mzee 1:** The cows are usually infected with Oltikana at certain seasons.

**Mzee 3:** Especially in the rainy season when there is grass.

**Researcher:** So, this is during the rainy season?

**Mzee 1:** Yes. Climate influences the cases of Oltikana, and the cases increase only in the rainy season rather than the other diseases in which the livestock are infected when it rains.

**Researcher:** Currently, can the cows be infected with Oltikana?

**Mzee 1:** He supported what we said.

**Mzee 3:** During the dry season, when they are infected, most of the infected cows die of the disease.

**Researcher:** Are there cases of Oltikana currently in this area?

**Mzee 3:** Yes.

**Researcher:** ... Why is it the calves?

**Mzee 1:** The calves are the ones that are mostly infected with Oltikana.

**Researcher:** Why are the calves usually infected with Oltikana?

**Mzee 1:** When the calves have stopped suckling milk, when they feed and start getting fatter, than they will be infected with Oltikana. This man is saying that when the cows drink water from the common areas, they may become infected with Oltikana.

**Researcher:** What factors contribute to the cows becoming infected with Oltikana other than the climate and the water you have mentioned?

**Mzee 2:** the fattened calves are the ones that are usually infected with Oltikana because when they drink the water, they will react with the fat from their tissues; thus, they will be infected with Oltikana.

**Mzee 3:** When the water is stagnant and the leaves are firm, this tree contaminates the water, which causes Oltikana.

**Researcher:** Are there certain leaves that cause infections in the cows?

**Mzee 3:** Yes. But they are not in this area.

**Mzee 1:** These areas are very different because here it is a semidesert called *inaudible.* When we take the cows to regions with a lot of grass and forests, when the leaves contaminate the water they drink, they cause a disease called ‘ugonjwa ya matawi’. Cows from this area cannot be infected with that disease because ewer do not have the trees in this area. Majorly Oltikana is caused by the small insects called Tsetse fly in the rainy season. This is because during the rainy season that is the time that there are higher cases. the flies sting the eyes and the wears of the cows thus causing them to be infected with Oltikana.

**Researcher:** Are there different types of Oltikana?

**Mzee 1:** When the cows are infected with Oltikana, some will be blind. Sometimes the nodes of the cows will swell.

**Researcher:** Are there different types of Oltikana or are they just signs and symptoms of Oltikana?

**Mzee 1:** They are different types of Oltikana because when the cows are infected with the one that causes the swelling, we say the it is Oltikana Olodua and this is the deadliest. With the one that affects the eyes, the cows are cured of the disease than the previous disease. When the cows are infected with the previous one, we even inject the red medicine that costs five hundred shillings but it is not a guarantee that the cows will get well.

**Researcher:**  Do you have any other type of infection?

**Mzee 2:** Thers is also another disease that affects the goats and it is called Oromilo. Both the sheep and the goats are infected.

**Researcher:** Is the disease curable?

**Mzee 2:** No, it is not curable.

**Mzee 1:** It causes convulsions.

**Researcher:** Can it be called rabies?

**Mzee 1:** Yes.

**Researcher:** You had mentioned that you take your cows to Mara and Mau to graze during the dry. Are there cases of Oltikana in that area?

**Mzee 1:** Yes, there are many cases of Oltikana because in that area there is wildlife conservation. So, the Tsetse flies transmit diseases from the wild animals to the cows.

**Researcher:** So, in Mara Oltikana is majorly caused by wild animals?

**mzee 1:** Yes.

**Researcher:** So, Oltikana from Mara is deadlier than Oltikana from this area?

**Mzee 3:** Yes.

**Mzee 1:** We cannot say that it is deadlier than Oltikana from this area but the issue is when the cows are infected with Oltikana from that area it is very hard for the cows to get cured of the disease than when they are infected with Oltikana in this area.

**Researcher:** That is in Mara, what about the one from Mau?

**Mzee 1:** In Mau, the cows are not infected with Oltikana but they are infected with the disease caused by contamination of water by the leaves called Mbinik. When the cows are infected, they will have their hairs rising, and they will produce blood from the mucus.

**Researcher:** What signs and symptoms will you observe t the cows when they are infected with Oltikana?

**Mzee 3:** The cows will lose their appetite, and they will have their hair rising.

**Researcher:** Are there any other signs and symptoms that you will observe on the cows on the mouth and nose when they are infected with Oltikana?

**Mzee 2:** Those are the only signs.

**Mzee 1:** When the cows are infected with Foot and mouth when you observe that the cows have watery eyes and they are crying and they are not producing froth as if they are infected with Olkirobi, and if they have their hairs are standing, you will then conclude they are infected with Oltikana.

**Researcher:**  What is the first thing that you do when you have discovered that your cows have been infected with Oltikana?

**Mzee 1:** I will administer the medicine.

**Researcher:** Which medicine will you administer?

**Mzee 1:** There is Terramysin, Adamycin and there is the white one called...

**Mzee 2:** penicillin.

**Mzee 1:** Yes, that medicine. When the cows have been infected with the disease for a certain period of time you will go to the veterinary doctor and they will give you the medicine to administer to manage the pain and to treat the cow.

**Researcher:** So, you first go and look for the specific medicine?

**Mzee 1:** Yes. You will go to the veterinary doctor and then you will describe the signs and symptoms of the cows.

**Mzee 2:** I have a question. What disease are you researching?

**Researcher:** I am researching the diseases bit Oltikana mostly. This is becoming we have the vaccine but before we administer it to the cows we have to consult with the villagers and talk with them.

**Mzee 1:** There is a disease called anthrax and we call it Emburuo and when people eat the meat from a cow that is infected with this disease then they will contract the disease.

**Mzee 3:** Also, when they drink the blood, they will be affected.

**Mzee 1:** we have the slaughterhouse but when my cows are infected with Anthrax, I usually burn my cows so that they are not consumed.

**Researcher:** What do you do when your cows have been infected with Oltikana and when you treat it the medicine is ineffective?

**Mzee 1:** There is a disease called Olomoroj(lumpy skin) that usually the sheep and the goats are infected with and it makes them have sore skin and when you slaughter them, they will also have sores on the intestines and internal organs.

**Researcher:** So, when the sheep and goats are infected with it you can slaughter and eat them?

**Mzee 1:** You can eat the meat from this animal because it is better than the cows that are infected with Oltikana.

**Researcher:** I have heard that some of you take the cows to the slaughterhouse when you have tried to treat them but they do not get well. Does the meat from these infected cows affect the people that eat it?

**Mzee 1:** It does not affect the people when they eat this meat.

**Mzee 3:** The cows that are infected with anthrax, will have low fat on their skin they will be thin, and the meat will not taste thus we burn the cows.

**Researcher:** Where do you get the information on the diseases and the medicines?

**Mzee 1:** Since we are farmers, we usually migrate with the cows to Naivasha. Mau and Mara during the dry season and other areas in Kenya. So, when we want you take the cows to any area, we usually know the medicine that we are supposed to administer to the cows and the infections that are in those areas.

**Researcher:** So, you have acquired all this information through the migration of the cows in different parts of the country?

**Mzee 1:** Yes.

**Researcher:** Is there a time that you get the information on the cows from other areas?

**Mzee 3:** yes.

**Researcher:** Which area?

**Mzee 3:** In Mara, some ticks usually transmit diseases from the wild animals to the cows.

**Researcher:** Is there a time that the people from this area usually come and educate you?

**Mzee 1:** Not quite.

**Researcher:** Is it something that you would recommend?

**Mzee 1:** Currently we usually ask the veterinary doctors because they are easily accessible. Because there are times that we call them and tell them to treat the cows. There are times that we ask for veterinary doctor experts who are recommended by the villages. These experts are usually very busy and they mostly, move with the fridge with the medicine. So, they come and count the number of cows to vaccinate or treat and then you pay them. The cost is usually per cow.

**Researcher:** This is the mode that they use to sell the medicines.

**Mzee 1:** Yes.

**Researcher:** Do you usually trust the veterinary doctor and the people from the agro vets?

**Mzee 1:** We only trust the experts while there are some that we do not trust because you may treat your goats will medicine from a certain area and then the goat becomes stiff, so you know that it is them and then you tell the other villagers.

**Researcher:** So, you usually talk about this among the villagers?

**Mzee 1:** Yes. Because recently, my goats were treated by a certain veterinary doctor and they became so fat that I sold most of them for not less than ten thousand shillings. So, when also the other farmers are praising him, we usually trust such veterinary doctors. When I asked him the medicine he used, he told me that it was the vaccine he usually administers to the goats every three months to prevent them from contracting the diseases. There was a time when one hundred and sixteen cows died because of the disease, and when I treated them, the medicine was not effective. So, when you vaccinate the goats, they will not be infected with the disease.

**Researcher:** Do you experience any other challenges as the farmers in this area, like the unavailability of medicines and the diseases affecting your cows? You mentioned that you do not trust some veterinary doctors. Are there any other challenges that you usually experience?

**Mzee 1:** The main challenge that we experience is when you find that some people sell the expired to the farmers and they do not inform ten farmers.

**Researcher:** Does this usually happen?

**Mzee 1:** Yes, it happens. You may find that the businessmen are selling medicines that have been overstayed in the agro-vets shop. This is why I told you that the cows when they are administered the medicine, may become sicker than getting healthy.

**Researcher:** Are there any other challenges that you experience other than begin sold expired medicine?

**Mzee 1:** There are no other challenges,

**Researcher:** What ability the price of the medicine? Is it affordable?

**Mzee 1:** As the Maasai, we usually love taking care of the cows, so the price of the medicine is not a bother to us.

**Researcher:** As I finish, is there a vaccine used to prevent the cows from contracting Oltikana, and does it cost between one hundred shillings and one thousand two hundred shillings per cow? Will you purchase the vaccine?

**Mzee 1:** When we want to vaccinate the cows, we usually vaccinate many cows from different farmers, which reduces the cost of vaccinating the cows.

**Researcher:** What if the price is nine hundred shillings? Will you purchase the vaccine?

**Mzee 2:** Yes, I will.

**Researcher:** All the cows?

**Mzee 2:** I will vaccinate all my cows. Because there are times that we treat the cows at three thousand shillings, nine hundred shillings are affordable per cow.

**Researcher:** What about you?

**Mzee 3:** I will also vaccinate all my cows.

**Researcher:** Lastly, what about you?

**Mzee 4:** I will also vaccinate the cows.

**Researcher:** I am done with the interview. Thank you for your time.

**[END]**
