# Supplementary material for: Beyond the jab: Unravelling the complexities of vaccine adoption for East Coast Fever in rural Kenya
Source: PLoS One. 2025 Jan 28;20(1):e0315906. doi: 10.1371/journal.pone.0315906 (PMC11774369; doi:10.1371/journal.pone.0315906)
Supplement: S1 Dataset — (ZIP) [file pone.0315906.s001.zip › Supporting information (R)/IDI/20230403_101960 IDI.docx]

**In-depth interview**

Researcher: Can you tell me during which months you have seen ticks on your cattle?

Man: It's mainly seen in July and November

Researcher: What season is that when there are many ticks?

Man: Normally, that season is when it is not raining, and there is a drought, and you see the ticks have increased. So, in that season, it's very cold again.

Researcher: Can the tick kill an animal?

Man: Yes, it can kill if you don't take good care of the animal by spraying. It can die because the tick also brings lice to the animal.

Researcher: Have you noticed any changes in tick infection on your cattle over the years?

Man: Yes, I have seen the change because nowadays, we have more potent medicine for spraying and injecting.

Researcher: How do you manage ticks on your cattle during peak infection months, and is there a difference in off-peak season?

Man: In the drought season, when there are many ticks, I spray the cattle once a week, and if the ticks don't feel the spray, I buy Terramycin and inject them. But in the off-peak seasons, I can spray twice a month.

Researcher: Which diseases do you think tick can cause in cattle?

Man: Yes, the tick causes oltikana to the animal, but if you don't spray your cattle, that is when they will be infected by oltikana because the ticks will be many, but if you spray your cattle frequently, they will not be easily infected.

Researcher: Which ticks cause oltikana?

Man: It is the dotted tick; it causes oltikana, and this tick comes with the lice, which are tiny and brown; they also cause oltikana.

Researcher: which are the top three significant diseases affecting cattle in this area?

Man: Oltikana, Olkipiei, and this is affecting cattle.

Researcher: What could be possibly the causes of oltikana?

Man: As I said earlier, the dotted tick causes oltikana and also stagnant water that the cattle use to drink, especially in the dams that we have, and it has not been cleaned for a while; when it rains, the dirty water is still there and mixes with the clean water it will be contaminated, and this will cause oltikana. It is also caused by green leaves, especially in the forest area. Sometimes, oltikana can be so severe to an animal, and it looks like 'olodua', and when it reaches there, you cannot treat for yourself, but you need to call a veterinarian to check on the animal.

Researcher: What is your initial course of action when you suspect the cattle suffer from oltikana?

Man: I just use the terramycin, and I always use any available available, be it 10% or 30%, and sometimes I can mix it. Another medicine that is allowed is Dexam because we have used this on the animals and see an improvement in them.

Researcher: How much time do you take to call a vet?

Man: I take a maximum of three days if the cattle is not improving. I will call a vet to check on the animal.

Researcher: How do you typically recognize an animal suffering from oltikana?

Man: The animal will have swelling on the body, and sometimes it has watery dug, the eyes become watery too, and in the nose, it has mucus and trembling of the animal. When the eyes are runny and have mucus, we say it is Olodua, and we use penicillin because it will help very much, as well as tablets we give to the animal.

Researcher: Will the animal be feeding?

Man: No, the animal will not feed because it's fragile and will lie down. It will not also drink water, and the hair will also stand.

Researcher: Can you share the frequency of oltikana outbreaks you have experienced in your herd?

Man: The time I experienced oltikana on my cattle was in the year 2020 because I tried to treat the infected animals by myself, but they didn't get well, so after a while, I called a doctor, but it didn't still get well, and it died.

Researcher: Have you noticed any changes in the frequency of oltikana outbreaks in your cattle over the years?

Man: There have been considerable changes over the years because as time goes by, we have substantial dips (acaricides) that we use to spray the animals, reducing the tick infestation in the cattle minimizing the chances of oltikana in the cattle. Also, with the clean water that we now have, everyone has their dams, which are only used by their cattle, and this has reduced oltikana because in the past years, cattle used to drink water from the same place, which made the transmission of oltikana in the cattle.

Researcher: Are there specific areas in your village where oltikana outbreaks are more prevalent?

Man: Oltikana is more in Masaai mara (lemek)

Researcher: Why do you think this is the case?

Man: This is experienced because of the wild animals in the place, and they carry the ticks with them, so they shed them in areas where the cattle are going to graze. With that, the animals will have so many ticks on them. And this causes oltikana in the animals.

Man: I migrated them to Masaai Mara in the year 2010 and 10 cattle of mine died at that place, and this affected me because almost all the animals were sick.

Researcher: What did you do afterwards?

Man: I returned the cattle home and used Terramycin and another white dose, which I frequently gave to see an improvement in the cattle. I sprayed them sometimes to ensure the animals did not have ticks. With no ticks, they could not transfer the disease to the other animals.

Researcher: Are there any other diseases that farmers in this area are more concerned about or have experienced?

Man: There is Olodua, which affects the cattle in this area. It is not treatable because we have tried treating them, but they don't get well. And the cattle also become blind. It’s like the disease has no cure

Researcher: How do these diseases compare to oltikana regarding their impact on livestock and livelihood?

Man: Oltikana is better than Olodua in terms of both livestock and livelihood because oltikana is easy to treat and doesn't affect all the cattle at once. Hence, treating the infected animal is not very expensive. But Olodua is a very bad disease because it is not treatable, and it affects the cattle very much and sometimes all the cattle at once, and it ends up killing other cattle.

Researcher: What is the initial course of action when you suspect your cattle is suffering from oltikana?

Man: At first, I will inject the animal by myself, and if the animal is not getting better, I will call the vet for more checks of the cattle.

Researcher: Do you have a challenge calling the vet, and are the medicines you buy expensive?

Man: Sometimes the vet is available, and sometimes he's not, so when the vet is not there, we inject the animal ourselves. The medicine we use is not very expressive, so we just go to a nearby agro-vet shop and buy the medicine.

Researcher: Are you aware of the existing vaccine for oltikana?

Man: I have not heard of the oltikana vaccine. I just know the medicine used when the animal is sick already. What I say can prevent oltikana is frequent spraying of the cattle to ensure that they don't have the ticks.

Researcher: Are there other ways you have tried to prevent oltikana?

Man: Yes, in the past there were traditional herbs for oltikana. But now we also ensure that we migrate to areas that do not have a lot of oltikana cases.

Researcher: which other places?

Man: Like the Mau, the place has oltikana but it’s not as severe as oltikana from the Mara region. You see, in addition to having the most severe cases of oltikana, Mara also has very many diseases that could affect our livestock and some like Engati (Malignant Catarrhal Fever) do not even have a cure. So, we move our cattle to the Mau because it’s a bit safer.

Researcher: Do you think these methods of prevention are effective?

Man: of course, yes, as I mentioned, there has been a decrease in the cases of oltikana in this region.

Researcher: Would the vaccine also help?

Man: Yes, it could also help especially in areas like the Maasai Mara.

Researcher: How about in this area?

Man: Yes, it also can.

Researcher: if the vaccine was there and it went for between 1000-1200kshs (7-9$) would you purchase it?

Man: Per cattle?

Researcher: Yes, per cattle.

Man: But why would it be that price? The other vaccines are cheaper! I don’t think I would purchase it unless the spraying and the medicine stop working. What we are doing is working, so for now I do not think there is a need for the vaccine.

Researcher: What price would work for you?

Man: it should even cost 1$ and below like the other vaccines.

Researcher: Alright thank you for your time.
