# Supplementary material for: Beyond the jab: Unravelling the complexities of vaccine adoption for East Coast Fever in rural Kenya
Source: PLoS One. 2025 Jan 28;20(1):e0315906. doi: 10.1371/journal.pone.0315906 (PMC11774369; doi:10.1371/journal.pone.0315906)
Supplement: S1 Dataset — (ZIP) [file pone.0315906.s001.zip › Supporting information (R)/IDI/20230403_101953 IDI.docx]

**IN-DEPTH INTERVIEW**

R: What challenges do you get in this area while keeping cattle?

P: The first is the challenge of drought, which is the biggest problem. When we lack rain, grass will disappear, and the cattle will be weak. That is the major problem we are facing. Drought

R: What about cattle getting sick?

P: Cattle is getting sick. There is the disease of malaria, but nowadays, we use Terramycin, which reduces the issue of Oltikana (East Coast Fever (ECF). But there is Oltikana for in cattle.

R: Do cattle get sick often of other diseases?

P: Yes, there is another disease called Olkirobi,

R: What about the prices of drugs for cattle? Is it cheap or expensive?

P: There are those which are expensive, and we give to cattle to drink; that one for Oltikana is costly; it is not cheap

R: Apart from Olkirobi (Foot and Mouth (FMD) and Oltikana, which other diseases infect cattle here?

P: There is another one called Kububuo, but during the rainy season, when there is grass, and the cattle go to drink at the river where there is dirty water, it will be infected with that disease called Kububuo.

R: What about Orkipei (Peste des petits ruminants (PPR)? Is it there?

P: Orkipei is for sheep. It is not for cattle; it is mostly

R: What of Olomooroj (Lumpy skin diseases (LSD)?

P: Olomooroj is also primarily for sheep; we have never seen it in cattle

R: What are the signs and symptoms of Oltikana in cattle?

P: You will see the cow shedding tears from the eyes shedding watery saliva from the mouth; the cow will lack appetite and not graze on grass. The cow will excrete watery cow dung. You can also find a cow excreting cow dung with mucus and blood patches. You will find a cow with such signs

R: Will it be grazing usually?

P: The cow will not graze at all. You will find the cow not grazing completely

R: What will the skin texture look like?

P: You will see the fur standing erect,

R: With the period you have been keeping cattle, what causes Oltikana?

P: You will find. There is...there is... I don't know what to say,

R: You can even say in Maasai

P: There is that fly; when the cow goes to drink water, it will get many flies, and the cow will get...

R: What of something like ticks does it cause?

P: Even ticks cause, you will find ticks in a season, where cattle has so many ticks, it also causes Oltikana

R: Is there a season that Oltikana outbreaks are so high in cattle?

P: It has a season

R: Which season?

P: During the drought season, you will find the ticks infect cattle a lot, and the cattle start to be infected with Oltikana

R: What else?

P: Wild animals are the ones which attract those ticks to a large extent. Wild animals, when cattle mix with them, you will see them getting ticks, and then they will be infected with Oltikana

R: What is your initial course of action when you suspect your cattle suffer from Oltikana?

P: The first action is to spray cattle. You will look for dip and spray your cattle; the second is to look for the vaccine to inject

R: Which drug do you inject?

P: Terramycin is the one we use primarily to relieve the cow.

R: What if you inject it, and it does not work? What do you do?

P: There is no other option. You just leave the cow until it dies

R: Is there a time when you call a veterinary doctor to treat your cattle?

P: In the past, we never had veterinary services, but these days, we have young men who have studied veterinary medicine; that is what we call these days. When you see a disease has prolonged for long, that is when you call the doctor

R: How long will you wait before you call the veterinary doctor when the course of action does not work?

P: When you see a cow has stayed for two weeks, that is when you call a doctor

R: Is there another option for treating cattle apart from this Terramycin?

P: There is none. It is the doctors who tell us to buy other drugs which treat Oltikana, like Terramycin

R: For example?

P: I have never used it and know its name

R: What measures do you take to prevent Oltikana outbreaks in your cattle?

P: These days, we have the practice of fencing farms so that it does not mix with wild animals so that they don't get ticks and spread Oltikana; fencing is wanted we have embraced

R: What of spraying?

P: Spraying cattle after every one week, you spray your cattle. We try to prevent ticks, which are responsible for oltikana. But in case the cattle get oltikana, especially during migration, we tend to use stronger drugs. However, they tend to be expensive, but we only buy them when we have to migrate. Otherwise, cattle from this place rarely get oltikana. We, however, struggle with other diseases, mostly olkirobi.

R: How do you get information about any changes in the health condition of your cattle?

P: It is these doctors whom we call, and they tell you about the cattle problem. When we notice the cattle is losing weight and not feeding usually, that is when we call those doctors

R: I understand you have a wife in your household.

P: Yes

R: Who makes decisions regarding cattle breeding, feeding and management?

P: We just discussed that as the husband, I usually buy these things, like cattle breeds and pasture. I am the one who takes charge, and I will assign the wife to graze the cattle and milk them while they are home, but in buying them, it is me.

R: So you discuss with her when you want to do all these tasks?

P: Yes

R: Why don't you decide everything alone now that you are the head of the household?

P: It is true, but also the wife, she owns the cattle; she is the one I will leave them with to take of them; she is also an owner

R: Which ones of your sheep and goats can you say are yours, and which ones belong to the wife?

P: We don't have that; all are mine; cattle are ours, that is what I said

R: Is it yours, both of you or is it yours alone?

P: It is ours, the both of us. In this family, there is no cattle for the wife. There is no my cattle; it is for our family. It is that way

R: What of chicken, does she have?

P: Chicken belongs to the wife

R: Why can't chicken be yours, both of you as a family?

P: It cannot be

R: So, chicken belongs to the wife?

P: Yes

R: What of the rest; cattle, goats and sheep?

P: It is ours

R: With the livestock that you have in your household, how did you gain them, did you buy them, did you receive as an inheritance?

P: It came like that; it can be an inheritance from my father, and I took care of them. There are those I bought, I gained like that

R: Do you have cattle that belong to the wife alone?

P: There is none, it cannot be

R: Why can't it be? Why don't you allow the wife to own them?

P: In our culture, there is no cattle for the women

R: With the cattle, goats and sheep that you have in your household, how does it benefit the women?

P: First, we have these coolers, when the cattle are milked, there is milk the wife will get and she will take it to the cooler and the money is hers, she plans the income.

R: How does she spend the income?

P: She can take it and pay for her children school fees and buy for them clothes and even books, that is how she spends it.

R: Who deals with milk mostly in the household?

P: It is the woman

R: So she is the one who decides if she will sell or will cook tea with it?

P: Yes

R: When she gets the income, who will tell her how she will spend the income?

P: She is the one who will decide

R: You will not ask her to follow up on how she has spent it?

P: No, I will not

R: Do you ever ask your wife on how she divides the milk for milking and the one for the calf to breastfeed? Or you don't follow up on that?

P: I will follow up on that, because if she milks so much, even that calf will lack milk to breastfeed on and it will not grow, so I will follow up a lot.

R: How does she divide the milk for consumption and the portion for the calf to breastfeed?

P: A cow has four breasts, I can tell her to milk from two breasts and leave the other two for the calf to breastfeed

R: Who makes decisions on sale of cattle ?

P: It is me

R: Why is it you who will decide?

P: I am the owner of the household and I will be the one to decide

R: Which cattle do you sell; a cow that is milked, a calf or a bull?

P: A calf, there is a cow that has stayed for long and it has reproduced many times, that is the one to be sold

R: After selling the cattle, who makes the decision on the use of the income?

P: It is me

R: You will not discuss with your wife?

P: We will discuss but the decision is mine

R: Is there a point when the wife gives you the income, is there a way it helps in settling household bills?

P: Yes, there is a time she will buy sugar, she will have helped me there. There is a time she will pay school fees for children, it is a matter of helping one another.

R: From the time, the wife started selling milk, have there been any changes in the household?

P: Yes, there have been changes, the time when she pays school fees for children, there she will have helped me. We will no longer sell sheep and cattle, there is that milk. There is a time she will purchase with that income, that is help

R: Between cattle, goats and sheep, which one do you sell at first, when you have an activity that needs money?

P: It is sheep

R: Do you sell alone or you combine together with your brothers?

P: No, I just sell alone

R: Which market do you sell to?

P: Ewaso Ngiro

R: Whom do you sell to?

P: There are those people to sell to, like the Muslims, they buy and there are other people who come from far, like Kisumu and come to buy, there are others who come from Uasin Gishu and come to buy.

R: What makes you to sell your cattle to Ewaso Ngiro and not any other market like Ololulunga or Naroosura?

P: Ewaso Ngiro is a market that is near, also Ewaso Ngiro is a big market

R: So Ewaso Ngiro has good prices?

P: Yes

R: What challenges do you experience when looking for market for your cattle and milk?

P: We have a problem of roads.

R: What recommendations would you give in order to improve the market for your cattle and sheep and sell them better?

P: We want a way like we have a platform where we will have a weight machine for cattle, so that the cow can be measured and its weight known and sold with the actual price of the cow.

R: The milk that your wife sells to the cooler, does she take it there by herself or you take it on her behalf?

P: There are motorcycle vendors who come to fetch the milk everyday

R: Is there a day that you take it on her behalf?

P: There is no day, there is that motorcycle come to collect.

R: Okay.
