# Supplementary material for: Beyond the jab: Unravelling the complexities of vaccine adoption for East Coast Fever in rural Kenya
Source: PLoS One. 2025 Jan 28;20(1):e0315906. doi: 10.1371/journal.pone.0315906 (PMC11774369; doi:10.1371/journal.pone.0315906)
Supplement: S1 Dataset — (ZIP) [file pone.0315906.s001.zip › Supporting information (R)/IDI/20230330_125042 IDI.docx]

**IN-DEPTH INTERVIEW MAN**

I: What challenges do you experience in cattle keeping in this village?

R: The challenges here... This year's climate has two signs on cattle: when the grass is green, it will be safe for cattle, but in the last two years, the drought has been so high, and cattle have been hungry. When cattle are hungry, they become susceptible to diseases. They can be infected with diseases such as Olodua, such that inside the lungs, the air ventricles will dry completely. This will happen when cattle are affected by hunger. You get that. When cattle are infected with Olodua, Oltikana will follow up and infect them.

I: So, those are the diseases that infect cattle mostly?

R: Yes

I: How about the price of drugs for cattle? Are they affordable or expensive?

R: They are expensive. There are two drugs; one is Adamycin, which is white, and it is costly, and another one is red Terramycin, which is also very expensive. The small bottle of the white drug costs 700 shillings, the black one and the white one cost 900 shillings. The one that has 10 per cent concentration of syringes. The latter has fifteen syringes that go for 900 shillings. But, when you buy and treat it to your cow, the cow will heal after a few days.

I: Do diseases such as Orkipei (Contagious bovine pleuropneumonia) CBPP) and Olkirobi (Foot and mouth (FMD)also infect cattle here?

R: They cannot infect cattle. Orkipei infects goats, and it is in this village. It can infect them when they go to graze around the hill. It will infect all the cattle because all of them drink from one dam, and all goats in all households will be infected because they drink from the same water source. Orkipei does not infect cattle; it infects goats. And it has no other drug. The drugs we use are the ones I told you about, and their efficacy is when the animal is in the window period, but when the infection increases in the animal, the drug will not be effective at all.

I: What about Olomooroj (Lumpy skin)? Does it infect cattle?

R: Olomooroj infects cattle and sheep. But honestly, we have not encountered any outbreaks this season. Olomooroj infects sheep a lot, but due to climate changes, it also infects cattle. In 2017, this village had a high outbreak of Olomooroj in cattle. But, in the years that followed, Olomooroj infected sheep only.

I: In the last two years, 2021 and 2022, which diseases have greatly affected cattle?

R: Both cattle and sheep or?

I: Cattle alone? Olkirobi affects the mouth and foot of cattle, and it causes a lot of swelling of feet to the extent of trampling of the hooves. It can severely infect cattle for three weeks and is very dangerous. When it infects a pregnant cow, it can cause the cow to miscarry the pregnancy. It is a very dangerous disease

I: That is Olkirobi. Are there any other diseases?

R: There is Olkirobi, Olomooroj, Olodua and Oltikana. You know Olodua (PPR) and Oltikana are identical; the naming makes it different and is a very dangerous disease.

I: What are the signs and symptoms of Oltikana in cattle: the mouth, nose, and appearance of the eyes?

R: For Oltikana, the fur on the hide will stand erect like a dog's skin. The cow will not feed on anything; it will not drink water or graze on grass. It will cause body weakness. For a cow that is milked, it will lose milk completely. Even the calf will be very weak.

I: What will the appearance of the mouth be?

R: The mouth will be dry like a stick

I: It will not have saliva.

R: Yes, it will not have saliva

I: In your opinion, what causes Oltikana in cattle?

R: When a cow drinks dirty stagnant waters, or when it feeds on some insects on grass, the insects fly about and bite cattle. That will cause cattle to be infected with Oltikana

I: When cattle are infested with ticks, will they be infected with Oltikana?

R: Yes, you see the ticks that infest snakes, and this other... You know there are three groups of snakes; there is one that is black, and there is another one that is brownish, right? The brownish one has poison and the ticks that infest that snake, and it goes to bite cattle, it will cause Oltikana a lot. During the outbreak of Oltikana, it is better to spray your cattle with dip, to kill the ticks and they will not be infected by Oltikana. Even the tick that bites a snake and it comes to bite cattle, will not cause any disease harm. So, it is better... But it is very dangerous.

I: Are those snakes around the village?

R: Yes, there are many

I: When you go to graze your cattle, do you meet with them?

R: We do meet them because the ticks do not move with the snakes. They remain on grass and bite cattle more so when grass is long, they will have so many ticks in them. And even the snakes will be there.

I: You said you use Terramycin to treat Oltikana, is there any other drug you use to treat the disease?

R: There is no other drug. It is only Terramycin that we inject

I: Is there a time, you treated with Terramycin, and the cow did not heal?

R: Yes, we give it a span of three days, you can treat it either in the morning or evening then you wait the following morning to check on it. When I notice a cow has signs of a disease, I will treat it's then tie it with a rope in one place so that it will not go to feed on grass nor drink water and it also so that it cannot go anywhere.

I: Is there a time you have called a veterinary doctor to come check on cattle when it has not healed?

R: No, I have never done that

I: Do you have any traditional herbs that you use to treat Oltikana?

R: There is another drug that treats, you see this iron rod, when you put it on fire, there are some diseases it will manage. When you see a cow is infected with Oltikana mostly, we will heat this rod then I will press its hide all around the body and I will put these marks, starting from the head up to the fur on the tip of the tail. I will cut the fur on the tail until they finish, and the tail will be without any fur. That is also a treatment option for Oltikana

I: It will treat Oltikana in cattle?

R: Yes, it is a drug also. It will heal

I: What! So, when a cow gets infected, what will be your first course of action?

R: We will inject first Terramycin, if it does not respond, I will use the heated rod. One must work

I: What do you do to prevent cattle from being infected with Oltikana?

R: We will spray thoroughly, every week we spray

I: How many times in a week?

R: Two times in week, in a month four times. And it will prevent Oltikana

I: Right now, when you notice a cow has changed...

R: You see during the season with long grass, you will spray cattle every week, twice in a week, Oltikana will not infect cattle

I: During the drought season and ow during the rainy season, when are Oltikana outbreaks high in cattle?

R: During the rainy season. When it rains and there is cold, when cattle drinks water that is stagnant at a place, it will be infected with Oltikana.

I: Which sources of information do you rely on when you notice strange symptoms in cattle?

R: There is no one I will ask, it is just me alone. You will just assess the symptoms of a particular disease

I: Do you know all the symptoms for all cattle diseases?

R: Yes, I know

I: So, you cannot ask any other person?

R: There is no need of asking anyone. I know all of them

I: How did you know all of them?

R: Those four diseases namely, Olomooroj, Oltikana, Kububuo for cattle. Have you heard of Kububuo?

I: Yes, I have

R: Kububuo is dangerous, it infects cattle when they go to drink water, and it will not heal. Even if we treat with which drug, as long as it drinks water, it will die.

I: Any other one?

R: So, there is Olomooroj, Oltikana, Kububuo, Olkirobi, only those. Four diseases.

I: Do you have a wife here in your household?

R: Yes

I: You have cattle as well?

R: Yes

I: So, between you and your wife, who makes the decision about breeding, treatment and feeding of cattle?

R: All those plans are mine alone. The wife just milks cattle, there is nothing else she decides

I: Why is that it is you alone who decides?

R: And she stays at home, I provide everything for her

I: Why is it you that makes all the decisions?

R: The wife does not know anything. When a cow does not produce milk, she will ask me why is a particular cow not producing milk. It has produced a small amount of milk as compared to the other days. All the decisions are mine alone. The main role of the wife is to milk the cattle, if she does not, she will ask questions to me. For example, is this cow sick today? There is no milk. Like that.

I: How is cattle ownership distributed in your household?

R: All the cattle are mine here

I: So, you have cattle, sheep and goats?

R: Yes, I have all of them.

I: Do you have chicken?

R: Chicken belongs to the wife, but you see the time I brought my wife from her maternal home and married her, it is a must, she be given three cattle, ten goats and five sheep. It will be hers and also mine, but she cannot do anything with them.

I: So, it will be part of your herd?

R: Yes

I You have your wife has chicken?

R: Chicken is for the wife; I don't know about chicken.

I: It is for the wife alone.

R: Yes, it is the affairs of the wife

I: How did you obtain the cattle and goats that you have? Was it an inheritance from your father or you bought them?

R: I received a share of my father's inheritance, and I bought others. I did go for work and bought about forty sheep and seven cattle, but the rest I was given by my father. He gave me it completely.

I: Why can't your wife or women own their cattle?

R: No, No

I: Can't you give her now?

R: I will give her but... I will give her by name only. I will name some cattle after her. But she cannot do anything with it. If she has a friend, she will gift them, but she cannot give them. If she wants to do anything, she must ask me.

I: Why is it that the wife can only own chicken and not cattle?

R: The wife knows how to take care of the chicken alone. But she does not know how to take care of cattle. You know cattle has much expenses, you see during this rainy season, you need to lease from people land for pasture, but the wife does not have money to pay for all this work.

I: How do you see the cattle and sheep that you have to benefit your wife here?

R: It will help because you see this time when she gets children, I will provide for her through the cattle until she is fully satisfied because of the wealth of cattle that I have.

I: Are you meaning when cattle get calves?

R: Yes

I: Am asking, for example, does cattle help you in getting food?

R: No, No

I: The food that you eat here, how do you get it?

R: No, we cannot give cattle food. You see this cactus, that is found in the bush, during drought season, cattle will feed on it

I: How do you get food such as maize floor, sugar, salt?

R: I just buy them and bring them home. During the market day like on a Saturday, I will shop for food stuffs that will sustains until the next Saturday. There is nothing that I will borrow from neighbors, if my wife needs clothes, I will go buy for her during the market day until she is happy. If the children need clothes, I will go buy for them. If there is a sick person, they will go to the hospital on Saturday. All food, I know where to get them. During the market day, I will buy enough food; we will not borrow anything here.

I: Is there a time that the wife buys food by herself during the market day?

R: There is, you see like this rainy season, my wife will get more milk, and she knows where to sell it, she can even earn 10,000 of her own from milk and it will be hers. She will sell the milk to the cooler.

I: When she gets that milk, is she the one that will decide whether to sell it or consume it here at home?

R: It is just her who will decide, the decision is for the wife

I: When she gets the income like that 10,000, how will she use it?

R: She will know how to use it on her own

I: You will not ask her?

R: No, I will not

I: When a cow gives birth, you will start getting milk, do you ever tell her how to divide the milk for the household and the one for the calf?

R: You see this calves of cattle, a cow has teats which are erect, the calf will breastfeed from two and my wife will milk from two.

I: If you wish to sell cattle now, who decides on that?

R: It is me who will decide. If I wish to take cattle tomorrow to the market, I will not ask my wife, I will just take direct

I: After selling the cattle and getting the income, will you ask your wife of how you will spend it?

R: That is unheard of. I can even sell five cattle, and I get 100,000 shillings, that budgeting is mine alone. There is no one I will consult. When my wife notices that some cattle are missing from the herd, and she asks me if the cow got lost, I will just tell her, I sold at the market. And that story ends like that. I will just inform my shepherd when taking cattle to the market.

I: When your wife gets income from sale of milk like the 10,000 shillings you told me, is there a time that she gives you money to purchase drugs for cattle?

R: She does not do that. She will only buy kitchen utensils such as cups

I: What changes have you seen from your wife's milk income?

R: Clothing alone and plaiting her hair and some household items. She never buys cattle drugs. Right?

I: What of food?

R: She does not buy. When I have travelled to a far place and she has income from milk, I will call her on phone and inform her to buy some food for the family. And that will be sorted

I: When you have an activity that needs money, will you sell, sheep, goats or cattle?

R: When I have an activity that needs money, I will sell cattle. I will also sell sheep depending on the budget of the activity. If I need 10,000, I can sell one goat. If I need 15,000 or 17,000, I will sell two goats for 7,000 each and get the money

I: Which markets do you sell to?

R: Ewaso Ngiro and Ololulunga

I: Do you sell on your own or you combine together with other people so as to sell together?

R: I will take on my own. I will transport via a vehicle on the road, and I will take to the livestock auction market.

I: The milk that your wife gets, which market does she sell to?

R: She sells to the cooler in Ewaso Ngiro. Or if she gets orders for people like in hotels, there is no other place to sell.

I: Who do you sell to the livestock that you take to the market?

R: I sell to businesspeople mostly who are of different ethnic groups. They will just come and look for cattle and sheep. Mostly it is these people come in the cattle auction market while another group in the sheep auction market.

I: Why do you choose to sell your cattle to Ewaso Ngiro and not any other market like Naroosura?

R: It is only Ewaso Ngiro that I take

I: Why do you take only to Ewaso Ngiro?

R: Because it is a good place and near and the business in Naroosura and Ewaso Ngiro is different because the business climate in Naroosura is not that conducive for trade people.

I: How do you compare the price in Naroosura and Ewaso Ngiro?

R: The price in Naroosura is low compared to Ewaso Ngiro. In Ewaso Ngiro the prices are good

I: What challenges do you get at the livestock auction market?

R: There are no challenges.

I: There is no day where the prices were low?

R: There is, there is. You see from January through February to March, the prices are very bad, but after March, business is very good. In cattle, sheep and even the sheep kids, will take to the market and get good income from their sale.

I: With the prices being low at some time, what recommendations will you give to address that?

R: You see, now during the rainy season; the cattle trade is good. The businesspeople will get good and healthy sheep. You know in commercial hotels, they do not want weak cattle, they will not take that order of such cattle. It is better... You see the period starting from January, is when business is very bad because farmers bring in a lot of cattle and sheep. After all, it is time for children are resume school. But now there is rain, and children are already in school, so, that issue will now be okay.

I: The milk that your wife sells to Ewaso Ngiro, does she take it on her own to Ewaso Ngiro or do you take it on her behalf?

R: She takes it on her own. My wife will come together with the other two women, and they become three so, each will have their own day to take milk from the other women.

I: There is no day that you will take for her now that you have a motorcycle.

R: I will take for her but not very often. I will take for her and, on such days, she must pay for the motorcycle. I will charge them a hundred shillings only. When she does not have the time to take the milk. She will pay because you see the day that they get the payment, she will not give me anything, so when she does not get an opportunity to take the milk, I will charge her a hundred shillings, and that story will be closed.

I: On the day you take milk for her, you said you would charge her.

R: Yes

I: On that day, will you be the one to receive the payment or will it be her?

R: That milk is paid every end of the week and on cash on delivery.

I: So, when the week gets to the end, they get the payment?

R: Yes

I: Okay, the questions are over, and you answered them well.
