# Supplementary material for: Beyond the jab: Unravelling the complexities of vaccine adoption for East Coast Fever in rural Kenya
Source: PLoS One. 2025 Jan 28;20(1):e0315906. doi: 10.1371/journal.pone.0315906 (PMC11774369; doi:10.1371/journal.pone.0315906)
Supplement: S1 Dataset — (ZIP) [file pone.0315906.s001.zip › Supporting information (R)/FGD/FGD 230621_1325.docx]

**FGD MEN 230621_1325**

**Researcher:** Which diseases do you know that the livestock is usually infected within this area?

**Man:** Foot and mouth disease.

**Researcher:** Is there any other disease?

**Man 2:** Olkirobi (Foot and mouth disease).

**Researcher:** Is foot and mouth also called Olkirobi?

**Man:** Yes.

**Man:** The sheep and goats are infected with Ormillo(coenuruses).

**Researcher:** So, are the cows and goats infected with Olkirobi?

**Man:** Yes.

**Man 3:** There are times that the sheep are infected with a disease that affects their mouths and produces a lot of saliva called Shamshami (Bluetongue virus).

**Researcher:** Is this disease common in this area?

**Man 3:** Yes. It has killed a lot of sheep.

**Man 3:** Also, Oltikana (ECF).

**Researcher:**  Is the disease also common in this area?

**Man:** Yes.

**Man:** There are different types of Oltikana. There is the one that affects the intestines.

**Researcher:** Which other type of Oltikana exists?

**Man:** There is one that affects the eyes,

**Researcher:** So, it affects the eyes when the cows are infected?

**Man:** Yes.

**Researcher:** Is there any other type?

**Man:** There is also Oltikana, which causes these areas to swell and affects the cows.

**Researcher:**  I have also heard of Oltikana, which affects cow dung. Is this not a type of Oltikana?

**Man:** This is the type of Oltikana that affects the eyes of the cows when they are infected with it, and I think you have already listed it.

**Man:** When you slaughter the cows that are infected with this type of Oltikana, then they will have a damaged liver.

**Researcher:** Will the cows also be affected by the eyes?

**Man:** Yes. They will become blind.

**Researcher:** Is there any other type of Oltikana?

**Man:** Another disease your cows are infected with is Olkipei (Contagious bovine pleuropneumonia (CBPP).

**Researcher:** Are the cows only infected with it, or was it all the livestock?

**Man:** The cows and the goats are infected with it.

**Man:** It is similar to pneumonia, which affects humans. However, cows and goats are the only livestock infected with it.

**Researcher:** The sheep are not infected with it?

**Man:** No, they are not infected.

**Researcher:** Is there any other disease that you have not mentioned?

**Man:** There is a disease that the sheep are being infected with within this area, and we do not know the name of the disease, but it is causing many deaths for our sheep. This disease has three signs: you may find that a sheep or cow will swell on their heads, and even their eyes will swell. When you prick that region with a needle, warm fluid will gush. There was a time when one of my cows was infected with the disease, and it had swelling. sow when I pricked the swelling, the pus poured out of the region, and it was very hot. Also, when the sheep are infected with the disease, they lose a lot of weight and become smaller. The sheep will be very small, and then they will not have the wool, which will fall off their bodies. The disease makes the sheep very thin.

**Researcher:** This disease has begun affecting sheep recently.

**Man:** Yes. The sheep were infected with this disease from three years ago. At times, when the sheep are infected, they will be affected mentally, and it will make them eat the soil, and they will be moving backwards.

**Researcher:** Is this the same disease?

**Man:** Yes. The disease manifests itself differently in different sheep. When the sheep are eating the ground, it shakes a lot because if they are infected, they will die shortly.

**Man:** When they are infected today in the evening, the following day in the afternoon, they would have died.

**Man:** They may die after four days,

**Man:** In a week, the five to ten sheep may die of the disease.

**Researcher:** in a week?

**Man:** Yes. Then the sheep will be infected after a month. Later in the other month, there is a time when many sheep die.

**Researcher 2:** Are the sheep usually infected with the disease in the dry season or in the rainy season kith that dies?

**Man:** They may be infected with the disease in any season, but the cases increase in the dry season. We have never known the name of the disease, and it has been three years. There was a time when it had rained heavily, and it made the sheep have bruised mouths both outside and inside the mouths.

**Researcher:** Did this happen to the sheep only?

**Man:** Yes. Since that time, the sheep have been infected with the disease. This has led to many sheep deaths because I had eight hundred sheep from my example, and now I have twenty sheep. All my sheep died of this disease, and I tried to treat my seep using all the medicines from the veterinary doctor bit I was not able to.

**Researcher:** For three years, it has caused so much death.

**Man:** Yes.

**Researcher:** has every farmer had their sheep infected with this disease?

**All Men:** Yes.

**Man:** every farmer from this area, Ngorongoro, Lemek, Mara, Narosura and all these areas, has had their sheep infected with this infection. But we have never known the name of this disease, and it affects us.

**Researcher:** They have never determined because of this disease.

**Man:** They have never determined the cause of their disease; they have taken a lot of blood samples from the cows, and they have also taken the sheep to Nairobi and Eldoret to be examined, but they have never given us any report on this disease. The sheep are still dying of the disease.

**Researcher:** What do you do when your sheep are infected with such a disease because you don't have the medicine? Do you just let the sheep die or do you sell them?

**Man:** It is very hard to take all the sheep to the market to sell them. You may take very few to the market to sell them while some will die of the disease and in the end, almost all your sheep will die of it. Another symptom of the disease is sheep will have diarrhea.

**Man 2:** They will have diarrhoea until they die.

**Man:** That time when the sheep’s diarrhoea they cannot be treated using medicine. At this point the disease is incurable and they eventually die. When the sheep are about to die, they are usually very thin and when you slaughter the stomach of the cows, they will have the parasites, at the intestines. *Inaudible.* They will have the parasites.

*(People talking over each other)*

**Man:** We are not sure that it is the parasite that infests the sheep, but it looks similar to that one.

**Researcher:** I have noted that disease and I would like us to move on. Among the diseases that you have mentioned?

**Man 3:** There is also a disease that affects the farmers in this area, and it has also affected me. The disease manifests itself as a skin disease, and it is when *inaudible* enters the skin it has caused the death of five of my cows during the dry season and three or my cows are currently infected with it. It affects the udder and the tail of the cows.

**Researcher:** Are the cows infected with it in the dry season or in the rain season?

**Man 3:** In the dry season.

**Wazee:** They affect the cows at any time.

*(People talking over each other)*

**Man 2:** It affects the udder such that the calves cannot drink milk from the cows. This is for both the sheep and the cows.

**Man 4:** When the sheep are infected with the disease when they give birth, they may lose skin up to the udder.

*(People talking over each other)*

**Researcher:** Among the disease that you have mentioned which disease are the livestock mostly infected with in this area?

**Man:** All the diseases that we have mentioned are the disease that mostly affect the cows in this area.

**Researcher:** Do the diseases affect the cows multiple times a year or is it a specific time that the livestock are infected with the diseases?

**Man:** the livestock are infected with the diseases constantly and there are some that are even infected with these diseases currently.

**Researcher:** which disease is the hardest to treat among the three diseases?

**Man 4:** The disease that we have stated that affects the sheep does not have the medicine to treat them.

**Researcher:** So, this is the most difficult disease to treat?

**Man 4:** Yes. Also, Oromilo that the goats are infected with does not also have the medicine used to treat the goats when they are infected with it.

**Researcher:** So, the other diseases have the machines?

**Man 3:** Shamshami also does not have the medicine to cure the sheep when they are infected with it.

**Researcher:** What about the medicine that is usually written that it treats seven infections?

**Man:** They use that for branding because when we could not fond out the seven diseases that they treated, and they had not stated the seven diseases that the medicine treats.

**Man 15:** But that medicine is effective.

**Man:** The medicine is very effective, but the issue is we do not know the diseases that it treats.

**Man 15:** It prevents the cows from contracting several diseases and this is if you administer the medicine when they are healthy.

*(People talking over each other).*

**Researcher:**  So, they protect the livestock from infection if you administer the medicine earlier?

**Man:** Yes. *Inaudible.*

**Man:** He has not said that the medicine is not effective...

*(People talking over each other)*

**Man:** *Inaudible.*

**Researcher 2:** There is also a bird that really affects the farmers in this area.

**Man:** The bird really affects us and has caused the death of many cows.

*(People talking over each other)*

**Researcher:** The birds do not cause any disease, they just feed off the cows?

**Man:** Yes.

*(People talking over each other)*

**Man:** The bird will contribute to the transmission of the diseases because even if they fed on one cow when they feed to the next cows, they will transmit the disease.

**Man 4:** I was recommending that there should be medicine like pesticides that should be used on the cows that will protect them from the birds,

**Researcher:** Was that a recommendation or a question?

**Man:** We are asking if there is medicine or something that is used to prevent the birds from eating of the cows.

**Researcher:**  So, you have not gotten any method of preventing the bords from affecting your cows?

**Man:** The only, method of preventing the birds is chasing them away or killing them.

**Researcher:** For now let us note this then at the end I will give you the feedback.

*(People talking over each other)*

**Researcher:** Because there are different types of Oltikana in this area, what are the causes of Oltikana in your own opinion?

**Man 4:** When the number of ticks affecting the cows is numerous, the cases of Oltikana increase. The ineffective medicine that we are usually sold to treat the cows against Oltikana causes the extinction of the disease, so this also causes an increase in the cases of Oltikana.

**Man:** I will also add to what he said. The water the cows drink can also cause them to be infected with Oltikana. Because when they drink the stagnant water the is contaminated, they will be infected with Oltikana.

**Researcher:** Could you explain this more?

**Man:** When the stagnant waste is contaminated by the leaves of Elarai, when the cows drink this water, they will be infected with Oltikana.

**Researcher:** So, those are there causes that you have stated. Are there any other factors that contribute to the cows being infected with Oltikana?

**Man:** The ticks. These are the two major causes of Oltikana.

**Man 4:** When the ticks are on the snakes then they infect the cows, and they will transmit Oltikana.

**Researcher:** Have the cases of Oltikana increased or decreased over the years?

**Man:** The cases have reduced. But it will end if we get little help.

**Researcher:** What have you done to prevent your cows from being infected with Oltikana?

**Man:** We have built the Silanga.

**Researcher:** I know of washing the cows to manage the ticks.

**Man:** Yes, that is the method that we use.

*(People talking over each other)*

**Man:** Since everyone has a dam on their farms, each of us is usually cautious about the cows that drink water in them and we make sure that other cows do not drink water from our farms, thus managing the transmission of the disease.

**Researcher:** Is there any other reason?

**Man:** The cases have reduced because the farmers have fenced their farms, preventing interaction between the cows.

**Researcher:** So, interaction among the cows is a cause of Oltikana?

**Man:** Yes. When the cows drink from the same waterpoint and when they are having the salt, this may cause them to transmit the infection. So, fencing the farms has really helped in managing Oltikana. Also, washing the cows prevents the cow from being infected with Oltikana.

**Researcher:** How many times is it ideal to wash the cows?

**Man:** Twice a month. Recently, we have been having ineffective pesticides because you can spray the cows in the morning, and in the evening, they have ticks on their bodies.

**Researcher:** This is after you have sprayed the cows in the morning?

**Man:** Yes. You may have washed the cows in the morning and in the evening, they have the ticks. The medicine does not kill the ticks, but the smell of the pesticide repels the ticks, so as the days progress when the smell of the pesticide wears off then the ticks return to the cow’s body. So, currently, we even go to Tanzania to buy pesticides because in Kenya they are ineffective.

**Researcher:** I have noted that down. So, which signs and symptoms will you observe in the cows when they are infected with Oltikana?

**Man 15:** Their eyes will change the colour and they may be blue.

**Man:** Also, there are three more signs. The first one is that the cows will be very weak and you will observe that they will not be moving as the others and they will also lack the appetite.

**Researcher:** What about their water drinking habits?

**Man:** They will not drink a lot of water. If they are infected, they will have watery eyes.

**Researcher:** What about the nose and the mouth?

**Man:** In the first stages of infection, their nose will dry up. But as the infection proceeds, they will produce a lot of hard mucus. So, these are the signs of Oltikana.

**Researcher:** So, they will have a reduced appetite?

**Man:** Yes. When the cows have not been infected with the disease, they will eat little grass but if they are severely infected, they will eat grass. In the evening, you will observe that they are just weak, and they have not eaten grass.

**Researcher:** Since most farmers have ever experienced Oltikana, I would like each of you to tell me what is the first thing that you do when you realise that your cows are infected with Oltikana.

**Man:** When I have seen that the cows have their hairs standing, watery eyes and have lost their appetite then I start treating the cows.

**Researcher:** So, what is the first thing that you do?

**Man:** I search for the medicine and administer it to the cows.

**Researcher:**  Is there any specific medicine that you administer to the cows?

**Man:** Adamycin.

**Researcher:** What about you what do you do?

**Man 16:** I administer Terramycin, although sometimes it is not effective, so I have to try the red medicine that is used to treat Oltikana. You will observe the signs that we had mentioned till the cows will die of it.

**Man 15:** When we have observed that the cows are infected with Oltikana, we usually administer 10% Adamycin and some administer Adamycin 20%. If they are not effective then we administer Butalex, *inaudible,* to treat Oltikana.

**Researcher:** This is after Oltikana has not been effective.

**Man 15:** Yes. If the symptoms persist, we use the Butalex and *inaudible.*

**Researcher:** Are there times that those medicines are also ineffective?

**Man 15:** Yes.

**Researcher:** What do you do at that time?

**Man 15:** If the medicine is ineffective ten the cows are about to die.

**Man 4:** We go to the river and fetch water so that we can boil the cows when they do.

**Researcher:** What is the first thing that you do when your cows have been infected with Oltikana.

**Man 4:** In this area, every farmer when their cows have, been infected with Oltikana will administer Terramysin and usually we administer the highest percentage that has been written on the bottle. If it is not effective then we administer Butalex. If it is also not effective, then we just wait for the death of the cows but in most cases the drugs are effective

**Researcher:** Is there a time that you call the veterinary doctor to administer the medicine?

**Man 4:** Butalex is usually administered by the veterinary doctors. Currently, we have veterinary doctors in this area who usually call when we want our cows to stop to be treated when they are infected. So, after the medicine is not effective, we just wait for the death of the cows.

**Man:** Sometimes we decide to sell our cows when the cows have not been responsive to the medicine, and it is the time that they are about to die.

**Researcher:**  Is Oltikana from this area similar to Oltikana from Mara?

**Man 4:** It is the same type, but Oltikana from Mara is deadlier. This is because the farmers in that area they usually graze with the wild animals. Thus, there are higher cases of Oltikana from that area.

**Researcher:** Is there anybody that has another opinion?

**Man 16:** In Mara, there are higher cases than in the area because, here each farmer has their dam, and they have fenced their farms. So, this causes the reduced cases of Oltikana. But in Mara, they have not fenced their farms and they graze together thus there are higher cases of Oltikana. When you take your cows to that area and they are infected with Oltikana, they will hardly get cured of the disease.

**Researcher:** Is there a time in the dry season the the people take their cows to Mara to graze and they are aware of the cases of Oltikana?

**All Men:** Yes.

**Man 3:** There is a time that the drought may be too much on us that we have to take the cows to mara.

**Researcher:** So, you just take the cows to Mara?

**Man 3:** Yes, and they may get infected with Oltikana in that area, and some may even die of it.

**Researcher:** Is there anything that you do before taking your cows to Mara to prevent them from being infected with Oltikana?

**Man 3:** There is nothing that we do and there is no vaccine that we administer to the cowdom. We just carry the medicines that we use to treat the cows when they are infected with Oltikana.

**Researcher:** Are there a certain breed or age of the cows that are mostly susceptible to Oltikana?

**Man 15:** The Grade breed of the cows when they are infected with Oltikana will hardly get cured of the disease and they usually die of it the indigenous breed which is sometimes resistant to the disease is cured of the disease when they’re infected.

**Researcher:** Is there any other type of cattle that is susceptible to Oltikana?

**Man 4:** The Grade breed of the cows is usually susceptible to many diseases not just Oltikana because when they are also infected with Foot and Mouth, they may die of it while the indigenous breed does not die of it easily. We can say that the Grade breed is usually weaker than the other breeds.

**Researcher:** Can the calves be more susceptible to Oltikana than the matured cattle?

**Man 4:** With the calves, when they drink a lot of milk they may be infected with Oltikana but if you control the amount of milk that they drink, they will not be infected with Oltikana. *Inaudible.*

**Researcher:** I have heard that you have mentioned that there is no vaccine used to prevent the cows from being infected with Oltikana.

**Man 4:** Yes, there is no vaccine.

**Researcher:** If you heard that the vaccine is available in the Town Centre, and it costs between nine hundred shillings and one thousand two hundred shillings (7-9$) per cow will you purchase it?

***(****People talking over each other)*

**Researcher:** I would like each one to state their opinion on whether they would purchase the vaccine if it was being sold at that price.

**Man 3:** I will purchase the vaccine.

**Man 3:** Yes. So, as long as the medicine is not counterfeit and it is effective, then I will purchase it because I will be the one benefiting since my cows will not die of Oltikana.

**Researcher:** So, you will vaccinate all your cows?

**Man 3:** Yes, I will.

**Researcher:** What about you?

**Man 15:** I will vaccinate all my cows even when the vaccine is being sold at that price.

**Man 3:** The best option is to vaccinate the cows then they get infected and will die of the infection.

**Researcher:** So, if you have one hundred cows you will vaccinate all of them?

**Man 3:** Yes, I will. Because in the dry season, many cows will die of Oltikana, if there was a vaccine that protects the cows against it then I would have purchased it and administered it to my cows.

**Researcher:** Is there anybody that has a different opinion?

**Man 15:** I disagree with that because their medicine may be expensive, and it is not effective. Currently, some medicines are very expensive and they are not effective and we are not sure if that vaccine is effective...

*(People talking over each other)*

**Researcher:** So, you would not believe that the medicine is effective?

**Man 15:** Yes.

**Man 3:** He has said that he cannot buy the vaccine.

**Man 15:** *Inaudible.* Many farmers in this area usually vaccinate their cows, but that price is too expensive for some of the farmers thus they will not vaccinate their cows. Also, the vaccine should indicate the disease that it is protecting the cows against. It should state whether it prevents Oltikana or Oltikana and other diseases. But that price is very expensive. I would request you to have it sold at two hundred shillings per cow.

**Researcher:** So, according to you two hundred shillings per cow will be affordable?

**Man 15:** Yes, it will be affordable, and I would vaccinate all my cows. It should be sold at the same price as the vaccine that protects the cows against Olkirobi.

**Researcher:** Does anybody else have any other opinion?

**Man 4:** I will buy the vaccine if it is two hundred shillings but at nine hundred shillings, I may not purchase the vaccine for all my cows.

**Researcher:** So, two hundred shillings would be the ideal price for the vaccine?

**Wazee:** Yes.

**Man 16:** Personally, if I am told that the vaccine is effective and prevents the cows from being infected with Oltikana and I am supposed to vaccinate it every year or during that time, I will buy the vaccine.

**Researcher:** Even when it is being sold at one thousand shillings per cow?

**Man 16:** Yes, I will still buy the vaccine. If the vaccine is effective then I will vaccinate all my cows.

**Researcher:** I have heard many of you complain that you do not trust the agrovets and the medicine that they sell you because most of the time they are not effective. So, how do you prefer getting the information on the medicines and the vaccines?

**Man:** I will vaccinate my cows to see whether the vaccine is effective. If it is effective, then I will influence Man 4 to use the vaccine on his cows.

**Researcher:** Has everybody understood the question?

**Man 3:** We have understood the question but when you have enrolled on the use of the vaccine, you should first use them in Mara because there are many cases of Oltikana in that area than this area. If the farmers of that area tell us that their cows have been protected from Oltikana and the cases of Oltikana are lower, then we can administer the vaccine to our cows. Because in this area, you will hardly hear of cases of Oltikana unless we have taken our cows to Mara to graze in the dry season. But in this area, there will be no rush for the farmers to administer the vaccine. We have other diseases to worry about

**Researcher:** So, regarding the question I asked, information is usually passed from the chief, the radio, the village elders and other means. How would you like that information to be passed?

**Man 4:** Through the village elders.

**Man:** Through the radio.

*(People talking over each other)*

**Man:** We usually trust the chief or the village elder, so we would prefer them to pass the information.

**Researcher:** is there any other means that you may prefer other than the radio or the chief and village elder?

**Man 3:** Because we so not know of the medicine is effective, could you give us the vaccine we administer it to our cows then if it is effective we can now purchase it.

**Researcher:** So, you are requesting that all your cows be vaccinated for free, and then you will purchase the vaccine later?

**Man 3:** Yes.

**Researcher:** Does anybody else have a different opinion?

**Man 4:** I believe most of us have a similar opinion. We have to test the vaccine and if it is effective, we will purchase the vaccine.

*(People talking over each other)*

**Researcher:** Will you purchase the vaccine if the veterinary doctors from this area tell you that the vaccine is effective?

**Man 3:** They usually deceive us.

**Man:** When they treat the cows, they usually die. So, we would prefer to use the vaccine first then we will know what to do afterwards.

**Researcher:** Is there any other factor that would make the farmers in this area not use the vaccine other than deception from other people?

**Man:** What we are saying is that when the cows are infected, and I go to the veterinary doctor and buy the medicine if is effective, if my neighbours’ cows are infected, I will recommend this medicine, and they will get cured. Through this network, the people will know that the medicine is effective.

**Researcher:** So, is passing this information on to other farmers, according to your experience, the best method?

**Man:** Yes. Because if few farmers know that the medicine is effective, they will use it, but if they administer it and their cows still die, then we will know that it is ineffective.

**Researcher:** I have understood what you are saying. Does anybody else have another opinion?

**Man:** Farmers will buy the vaccine regardless of the price. The factor that will determine the purchase of the vaccine is if the vaccine is effective.

**Researcher:** I would like to end the meeting at this time. Thank you very much for coming and for your time.

**[END]**
