# Supplementary material for: Beyond the jab: Unravelling the complexities of vaccine adoption for East Coast Fever in rural Kenya
Source: PLoS One. 2025 Jan 28;20(1):e0315906. doi: 10.1371/journal.pone.0315906 (PMC11774369; doi:10.1371/journal.pone.0315906)
Supplement: S1 Dataset — (ZIP) [file pone.0315906.s001.zip › Supporting information (R)/FGD/FGD 230622-1049.docx]

**MEN FGD TRANSCRIPT**

R: In this area of Olgilai, which is the most prevalent animal disease for which you face a challenge in its management?

MAN 1: The first disease here is Olkirobi (foot and mouth (FMD)), the cold for cattle. He knows the disease. The second one is Malaria or Oltikana because it results from, you see, even when you spray cattle, it cannot take time before you start seeing the ticks. Mostly, we have witnessed Oltikana come because of the issue of ticks. So, all people do not spray their cattle at the same frequency. There is one that can wait for two weeks or a month before you come to respray cattle. It will have ticks. And you see, it will be suffering from Oltikana. Those two diseases are primarily prevalent in cattle in Olgilai, together with drought, which has also been very high. Those three diseases have been so prevalent. Orkipei has not been that rampant, and if someone has looked after their cattle because of Orkipei, when there is an outbreak of Orkipei, let us say in Masaantare or Narok or any place. It is reported there, and people nowadays understand the importance of vaccinating. Nowadays, cattle disease management is not as complex as it was in the past. For the Maasai people in the past, it was hard to see the disease infecting an animal, but today, when it is reported, there is an outbreak of Olkirobi somewhere. Because it has started to reduce, it has diminished. And some people have advanced in vaccination for animals. The biggest problem we have is the issue of animal drugs because the available drugs have not satisfied us.

R: And now...

MAN 1: I say that because these drugs which you come to... Let us say now, you doctors when the doctors go and want to treat cattle and say there is a drug for Olkirobi because I do not want to lie to you, even the treatment has not satisfied us. After all, it does not take time. Also, there are other places we have also discovered that even if you bring the drug together with the veterinarian, you see that it will seem to last.

**R:** So, I want you to open up like him and express all your opinions. The village elder told us that there are two diseases: Olkirobi, Oltikana, and drought. Do we have another disease that affects cattle mainly?

MAN 1: I have said Orkipei but not so much because now, when an outbreak of Orkipei is heard somewhere, the drug for Orkipei is better off because when an outbreak of Orkipei is heard somewhere and people vaccinate cattle, even that disease will become extinct. It cannot infect cattle again. And even this Olkirobi, when I even vaccinate my cattle, and (look after the young sheep) when I vaccinate now my cattle and the other has not vaccinated theirs, it will make my cattle be infected again even if I had immunized them—something like that.

MAN 2: It will infect again.

R: And now this Olkirobi?

MAN 1: Another problem is that people do not vaccinate at the same time,

R: They vaccinate at different times,

MAN 1: Yes,

R: So, these three diseases you have said, Olkirobi, Orkipei, Oltikana, do they infect all livestock: sheep, goats, cattle?

MAN 1 Nowadays, it has come; Olkirobi infects all of them.

R: It infects even sheep.

MAN 1: Yes, you see something like that we use to feed the mineral salts; I want to inform you that Olkirobi has infected another sheep of mine. And you know, this basin is used to feed mineral salts to cattle; it is the one that has made all of them infected.

MAN 3 It infected the sheep.

MAN 1 Yes, it infected utterly.

MAN 3 And now,

R: Let us hear from another person.

MAN 1 It infects because when it gets infected in this cow shed, it will also infect the sheep. I know very well what he is saying.

MAN 4 Those two diseases infect so much. There is also another sheep disease called PPR, which infects sheep in the head and makes the sheep bulge; the head will swell; there are other diseases, and there is no drug for it. If you inject a particular medicine, the sheep will stay while it dies. So those are different diseases. There is also another disease for sheep called Olodua and also for goats. If it infects an animal, it will persist so much. It is another disease that is on the rise. Those are the other diseases in addition to what the elder has said. That one Oltikana, the Olkirobi and the cold has spread. It is almost everywhere. So, we have a challenge there.

R: Is there anyone with a different disease he has seen on his livestock?

MAN 7 There is because, like for goats, there is that madness that infects goats and sheep.

MAN 8 That one is Ormilo

Millicent: Ormilo?

MAN 8 Ormilo is on the rise for goats in this area.

R: It infects goats.

MAN 6 Yes, I do not even know what caused that.

R: Does it infect cattle as well?

MAN 2 It infects goats mostly of this young age.

MAN 8 Talk about Orkipei in goats. And also, Cattle

R: There is another one who had said...

MAN 6 It is that one

R: So, he has added Ormilo,

MAN 1 Ormilo is there nowadays but not on cattle; it has disappeared on cattle, please. What infects goats the most is goats; I do not know what has caused goats to become infected with Ormilo. I do not know if it is drugs or maybe other acaricides that are very strong.

MAN 3 Even if you treat with what drug, it does not respond to heal.

MAN 1 It does not recover.

MAN 4 Lastly, as the chairman said, when you come, when the doctors come to vaccinate livestock. You are supposed to give a date when you will come to vaccinate livestock in this area. So that the chairman may inform every parent nearby to get his livestock ready on that day, the doctor should not be in a hurry because when he comes in a hurry, he will only vaccinate in two households then leaves as the elder said, so you know the livestock will interact at the fields while grazing. Still, the neighbour's animal will infect the others again. So that is what I want you to know. Mine, I have my livestock, donkeys, they have been infected with this disease, anthrax. I would like, even when we finish this discussion, to go to my household and examine it so that you can tell me what the drug for it is. Yeah.

R: We will look into that as well. So, I want you to tell me.

P9: There is another one. I want to add another one, which the chairman said is the way doctors are... There was another day we went to Narok, and we were hosted at Chambai for three days with doctors. Many doctors were there training on livestock management issues and others. All the things they told us, even another one, said he was in charge of Narok. He said that the people of Narok South have that disease, and they never taught us about it. Honestly, the way they tried us that day, we told them all our problems. They wrote it down. It has been about two years, and we have not seen any doctor interventions. Nowadays, like me, I am told to attend another seminar for doctors. I could not even go because we went there for a week, and there were doctors for people, cattle, and others. They even took us to Lanet to benchmark livestock practices. They told us to fill out the papers, which we filled out, and from that day, we have not seen any help. When cattle become sick, they say you do this and that. If we, the Maasai, do not know how to treat cattle, then there is no other help. Nowadays, what we fear is this drug because this drug, like he says, is good. We do not need any other assistance; we will just go and buy at the agro vet, and we will come and treat our cattle. The worst part is when they bring that drug...

MAN 4 And you do not know how the drug helps in which way.

P9: That is the only problem we have seen; the issues have become so many.

R: Let us continue. We are lucky that you are addressing the challenges you are going through, and we are capturing them well here. So, I would also like to know which diseases you have told me to infect your livestock: Orkipei, Ormilo, Olkirobi, and Oltikana. Which one brings the most loss to the livestock when it infects cattle? And you find a problem managing it.

MAN 4 Most of the time, it is that Oltikana and Olkirobi.

R: Let us start with Olkirobi. When it infects cattle, how long will it infect them, and when you begin treatment, will it respond to the drugs?

MAN 4 It will respond favourably to the drug. When you get a drug that you have done research on, it will treat.

MAN 5 For the drug, the drug for Olkirobi, when cattle of this area are treated at the same time the outbreak comes, they will recover—another problematic disease, Ormilo, for goats. I have not seen a drug which treats it. Then, these small livestock, like goats and sheep, have their disease, which is terrible, like Olodua, which, when it infects sheep, and you treat it, and it does not respond to the drug, will die. And it makes it wrong; the animal will be impoverished, not heal, and will be useless. It may also cause the sheep to have diarrhoea, weakening its immunity until it dies. So, we see many diseases. Even the one called PPR, we have injected till you leave it to die and leave God to know their fate.

R: Are these diseases contagious, or do they only infect one cattle like Orkipei Olkirobi?

MAN 5 When it infects one cattle, like in this farm, it will infect the other cattle in the whole area.

R: Which disease mostly?

MAN 5 Olkirobi mostly is the most contagious,

MAN 2 It will infect many cattle and not one. It will spread to many cattle in a few days

MAN 3 Olkirobi is the one most prevalent till it finished all cattle.

R: With Oltikana, is it contagious, or it infects only one cow?

MAN 3 No, it infects one cow only

MAN 5 It can infect one, two, or three cattle

MAN 1 Oltikana outbreaks are high in the areas of Lemek Enkipai, where Oltikana is very bad.

MAN 7 That other one which infects the eyes of cattle, and it makes the eyes shed excessive tears,

R: Here, Oltikana does not infect cattle mainly?

MAN 5 It infects a few cattle, just a few cattle, some, not like there, it is not that dangerous

MAN 4 Malaria is present in cattle, but you find it does not infect all the cattle at once; you can find it infects one cow and can infect till the cow dies.

MAN 3 The one it infects mainly is these young calves, like here it can infect like, four calves, but in large cows, it is not that often.

R: And it is calves of what age or months?

MAN 3 Even these calves which are still breastfeeding, even this...

MAN 7 We can say calves between twelve months and below

R: So, twelve months calves and below? Does it often infect these big cows?

MAN 2 It infects but is not that rampant. It is not common

R: When you treat it, does it respond to the drug?

MAN 1 We have seen that the drug for Oltikana is better because nowadays we have witnessed another drug being used by doctors because that drug is not like this other drug, which I told you is not good—this one of Oltikana. We have seen it not to be that bad. Oltikana has only two drugs, and we have seen they are not that bad. It is effective.

R: Which disease is resistant to drugs?

MAN 8 I have another question.

R: Questions...

MAN 1 I told you earlier, if you want to understand, Olkirobi disease is resistant to drugs because let us say you are now a doctor, and I have told you I want you to inject me the drug for Olkirobi and you come with the drug. So that is the drug we have told you it has not made us satisfied because you came to inject and still it got infected. About the drug for Olkirobi, for you to get the drug from Nairobi, in Kabete, when you see me standing here, for me to get a means of getting the drug from Kabete, that is a drug that will help us because a cow can stay up to six months without being infected with the disease but this other drugs. Some people came to inject some drugs for farmers, and it was not effective. That is our biggest problem as cattle keepers.

R: I have that right. What causes Oltikana in cattle?

MAN 1 I have told you it is ticks, ticks. If I do not spray my cattle with acaricides, they will not lack Oltikana, like one or two. But if you spray it often, it will not be that frequent. Spraying is the factor that determines if cattle will get Oltikana. There are others when you spray...

(Background talk from the participants in Maasai)

MAN 4 Give the old man an opportunity to speak

R: Someone to explain to him in Maasai so he tells us his views

MAN 1 Just leave him because the old man does not understand Kiswahili and he is very old. I have told you the problem we have with cattle is the drugs are not effective. I don't know if you have understood that.

R: I understand that

MAN 1 The issue of Oltikana is ticks, when I spray my cattle let's say after one week or I spray after a month. People do not spray the cattle the same for example I can spray mine after one week, if it delays two weeks, and there is another who sprays after a month. Also, when spraying the cattle, even before one week ends, we find that the ticks are again on the cattle. And it is that tick which has caused Oltikana in cattle.

R: So, it is ticks which contribute a lot to Oltikana in cattle?

MAN 4 Yes

MAN 5 Yes

MAN 1 Yes, mostly

MAN 4 So, doctor the way the chairman is saying when there are ticks in cattle, you know the ticks are the bacteria carriers which results in Oltikana. So how what sample do you take from the sick cattle that you research?

R: There is the calves team led by a well-known local doctor who normally comes and draw blood samples, and they take it to the laboratory for testing, that is why you see them taking the blood samples. As you have said that it is ticks which causes Oltikana, so which time does Oltikana infections are most high on cattle? Is it during the rainy season or drought season?

MAN 1 During the rainy season

MAN 4 Mostly during the rainy season

R: What might be the reason?

MAN 7 When you spray cattle

P: I don't know but when you spray cattle during this rainy season, and there is plenty of grass, I don't know if it is the stagnant waters, but during the rainy season, that is when I have seen Oltikana infections being high.

MAN 4 And that tick, you know when there is grass, maybe they breed it the grass so the cow when the cattle go to graze, that is when they stick on cattle and then they start biting the cow.

R: So, during the dry season, Oltikana infections are not high?

MAN 4 During the dry season, Oltikana infections are not high

R: Any different opinion on that question?

MAN 8 There is another time when the cow gives birth before the maturity of the pregnancy, so we don't know, Oltikana is what causes the cow to give birth before the pregnancy matures and also when the pregnancy matures, the cow would still have unusual signs, it will have dirt, so I don't know if in the inside it is Oltikana is what makes the cow to give birth before the maturity of the pregnancy.

R: Now to the signs and symptoms for Oltikana, which ones do you see when a cow is having an infection of Oltikana? How will the body change?

MAN 1 It has two signs or three, on the eyes of the cow, till the eyes of the cow become green. And there is swelling of this muscle around the neck on the body and fur erecting vertically. The fur will not be in its usual posture, it will erect, and the cow will shiver, it has those three signs.

R: How do the eyes change?

MAN 1 It changes when you see the eyes when the cow if infected by Oltikana, it is the cow which is infected mostly by Oltikana. Till the cow heals so that the eyes turn to be normal,

R: Does it shed tears or not?

MAN 1 Yes, it does

MAN 4 It sheds tears, and the fur become erect.

MAN 7 And there is mucus that comes out

MAN 8 Nowadays there is Oltikana that infects cattle that is very dangerous, you will see the eyes of the cow become dark red

R: It becomes very red.

MAN 8 And that one when you inject the drug once and the drug is not effective, the cow dies next.

R: Does the cow graze normally and drink water

MAN 1 It does not graze now drink water, till it starts recovering that is when it will start grazing slowly and drinking water but when it infects the cattle, even the body temperature rises.

MAN 4 Chairman, what of this disease called Olgila, is it here mostly?

MAN 1 It is not there much

MAN 8 The old man is asking if you will come to vaccinate cattle.

R: Does the cow dung change when a cow is suffering from Oltikana?

MAN 1 Yes, it will.

R: How will it be?

MAN 1 It will be so dry, it will lack the moisture

MAN 4 It will be hard

MAN 1 It will be very hard

MAN 8 You can find it has some blood patches and also the cow dung may have a mucus-like substance, and also it persists. You can find it will be there for like three days or two and then time it goes to excrete, you will find it removing something that is hard like some red patches.

R: I heard there are some types of Oltikana, so these types have you seen it in your cattle, or you just saw one? I don't know if you have seen that here.

MAN 8 You were told that Oltikana has various types, you find there is one which makes the glands swell all over the body

MAN 4 The glands swell

MAN 8 The glands well all over the body, so that one affects a lot. There is this one that infects the eyes, and it makes them shed tears, and the eyes turn green. You get even the eyes become sunken that it will not see,

MAN 6 That is the worst.

MAN 8 So that one it goes till the eyes do not see, that is the worst and then you will find the cow being, so the Malaria is of different types. There is also this one where the cow erects the fur, and you find the cow makes the ears to lie and it will not be feeding. You will find this can go three days, or two days so that one is Malaria as well

R: As cattle keepers, is there anyone whose cattle have been infected by Oltikana recently?

MAN 8 Right now, no,

R: Not now perse, maybe last year? In the past.

MAN 1 You cannot miss that.

P8; For last year there is,

R: In your farm, how many cattle were infected?

MAN 8 Two

R: Were they calves or large cattle?

MAN 8 Large cattle

R: How did it go?

MAN 8 I did call the veterinary and he treated it till it healed.

R: Which month was it?

MAN 8 It was during the drought season. That time of dry season

R: This year or last year?

MAN 8 It was just this year, the times when grass was inadequate and we were giving grass to cattle, so they were saying it is this grass that we were given to cattle. We are saying so, but we saw sometimes when there is hunger and the cow lacks pasture, it may be infected with Malaria.

R: Which grass was that that you were giving to the cattle during the drought season?

MAN 8 That one that we got from the farms,

R: Not that one that was being sold.

MAN 8 We used to go and bring from the farm that is there.

MAN 4 There is that you cut from the farm and there is that you buy.

MAN 8 Mine I got it from that farm, we give it many things, vegetables, grass, all things that cattle can feed on. Not just grass alone that is available.

R: And the one you saw is available...

MAN 8 And there is some certain bean species which we brought, and we had put it for a while, I doubt that is what brought it.

R: Any other person, whose cows were infected with Oltikana?

MAN 8 That time I called a vet doctor who came and treated it.

MAN 3 Which disease relief is he talking about for cattle?

MAN 8 He is talking of the diseases that infect cattle because they are researching a vaccine for some diseases

MAN 2 In my view, if your organization wishes to help cattle keepers, if you deal with the diseases which affect us, with Oltikana I can go and buy drugs and come to treat it. Oltikana is not that contagious, and it will not affect many cattle. The diseases which are affecting us mostly are these; Olkirobi, because it infects cattle, it will infect all cattle all over this area Up to Lemek, if the government would commit to vaccinating cattle every month a lot often, that disease can be reduced. That is one. There are goat diseases, goats have some diseases which are a problem to us, there is Orkipei that infects goats, and every year we vaccinate goats twice, another one is Olodua for goats that we also vaccinate. If the government had a schedule for strong vaccination for cows, that one from Kabete, let's say every month or every year, they would have the vaccination campaigns, then we would get help. If those two diseases are dealt with that is Orkipei and Olodua, those other diseases I see are not a big problem.

R: Has your cattle even been infected by Oltikana?

MAN 2 No, it has not. There was one cow which was sick from Oltikana, but I injected that drug, and it healed.

MAN 4 For goats, during this rainy season, you will see it coughing it will be having mucus

R: And here, has your cattle ever been infected with Oltikana?

MAN 5 It has been in the past

R: Which year was it?

MAN 5 Last year, 2022

R: Last year is recent, how many cattle were infected?

MAN 5 One

R: Calve or large cow?

MAN 5 Calve

R: What did you do?

MAN 5 We injected some drugs including butalex, but they were not effective, and it came to be this one for eyes, till it reached a point, the sight was lost, and the nose was dry completely, and the body health was deteriorating, and even the cow dung changed completely, it became black and not watery. And it will not graze, so those are the signs of the disease.

R: Did you treat?

MAN 5 Yes

R: If you look at Oltikana outbreaks in the past and today, when were the outbreaks high? Is it this current years or the past years?

MAN 1 Not this side, but the sides of Lemek even now, the outbreaks are very high,

P9: Not this side.

MAN 1 I think the team of some researchers are aware because farmers from there have complained a lot, you hear Lemek and the surrounding areas.

P9: Just the entire Mara region

MAN 4 You know Lemek?

R: Yes, Lemek, in the Mara sides?

MAN 1 Yes, that one

R: Have you migrated your cattle to there, I understand there is a time you may migrate your cattle.

MAN 1 We migrated our cattle to their till we escaped from there and we returned them here because of that disease; Oltikana.

R: What is the reason for the high outbreaks of Oltikana in Lemek?

MAN 1 I don't know because there is another son of mine who migrated his cattle there and he returned them back home, he slaughtered almost ten cattle, and we never saw any problems in cattle, they just said, it is that Oltikana. Even now the locals there, complain about it. Even large cows and bulls die as well, not just calves alone.

R: So, the infections are higher there than here?

MAN 1 Yes, it is too much, we are better here.

R: You are better?

MAN 1 Yes, because it infects one or two cows, we are better.

R: Why is it that here it is better?

MAN 1 We don't know; we are also wondering. There are many forests here

P9: Maybe the climate

MAN 1 We don't know... When you

MAN 4 When you go to any place...

MAN 1 You know there, there are still large plains where cattle can be taken for pasture. When you take there where there are large plains, you will just return them...

MAN 8 What has helped us here is that we do not have wild animals here, like wild beasts they are not here. The only animals found here are hares. Even the antelopes are not here. So there, there are many wild animals, and they migrate a lot, they carry the ticks from another place, they bring them here and those ticks bite the cattle, so that is the... There is also a fly which when it bites cattle, it will have diseases

MAN 4 That tse tse fly

R: When you check the climate and weather of here and Lemek, how do they compare?

MAN 1 It is the same, it is hot like here

R: Which interventions did you use when you migrated your cattle there to prevent Oltikana?

MAN 1 You go and spray

MAN 5 You just go and inform the doctor,

MAN 1 By spraying only, you spray every week

R: How often do you spray here?

MAN 1 I told you; you spray like once a week,

MAN 8 Here it varies.

MAN 1 Here it is not that common like there, but there is one week

MAN 8 You know here, it varies, I can be spraying cattle after two weeks, and after that, I spray again without even getting the ticks. You will find another one, sprays after one week or one month. Others spray only when they see the ticks on cattle. So that is the difference among people. People spray cattle at their frequency. There you find, farmers spray cattle after a week, and there they use a drug which you can buy and you find that after you have sprayed, it will not stay long before it gets ticks again, so you spray again,

R: Another person, like you how often do you spray your cattle?

MAN 4 I was like two or three times

R: Monthly or weekly?

MAN 4 Monthly

R: How often do you spray your cattle?

MAN 6 Twice a week

R: Which days?

MAN 6 One Saturday till the other Saturday

R: So, it is once a week

MAN 6 Yes

R: Don't you have another method of controlling ticks on cattle?

MAN 3 It is just dip alone

MAN 8 If they become many, you buy this drug specific drug I cannot remember its name

MAN 7 Nowadays it has helped us a lot

P8; You inject the drug, that is what helps now, because you find that you spray dip, and it is not effective until you go and buy the drug and then you inject so as to control the ticks.

R: Do you use here the drug, or it is the farmers from Lemek who use it?

MAN 1 It is for health, not that you use it for controlling ticks...

R: Is it vitamins?

MAN 1 Yes, it is a vitamin to boost a cow's health. When you use that, it works, and we notice that it has reduced ticks. But it is not a tick; we use it for our health.

(Local dialect to explain the point on the use of the vitamin)

MAN 4 You see the dip for spraying. That is where you need to improve because you need to know which type it is, know that you can use that dip, and know that it will finish the ticks.

R: What pathways do you use to treat Oltikana? There is someone who said he injected the drug, and it does not hear. Do you have other more effective drugs?

MAN 1 We only have two drugs that have helped us a lot.

R: Which are they? What do we call this drug sold at 2,200kshs (17$). It is butalex, and another one, what is it called?

MAN 4 There is this red drug that ...

MAN 8 It is measured with a syringe.

MAN 1 What is this drug called?

R: Terramycin, penicillin

MAN 1 No, when you say the drug for Oltikana you will be given the two options

MAN 6 And it is expensive

MAN 1 Line a small bottle for 20cc...

MAN 8 There is that drug for Oltikana that they sell it...

R: And how much is it?

MAN 3 One syringe of 10 ml is 500

R: You have said there are two types, one is 500, the other is how much?

MAN 1 Which one?

R: You have said there are two types, what are their prices?

MAN 1 There is one that is 1,200

R: And the other one

MAN 1 The other one is 500

R: Which one is the most effective?

MAN 1 This one for 1,200, it is very effective.

R: Where do you get information on livestock diseases and drugs? And also outbreaks of diseases in certain areas, which sources do you use?

MAN 8 We will just get it amidst ourselves, like now the way I have come here, I will see the cattle of this household how they are. If I see they have diseases, I will say that in a certain farm, I saw that the cattle have this disease. So, if it is that disease it is dangerous because you will not complain about every disease. There are those like Orkipei, and Olkirobi which you will raise alarm about because they are contagious diseases. Malaria can infect cattle on this farm, and it will not infect the neighbours. And you see, they are one; it will only infect certain cattle in some farms. But if it is Olkirobi, when I come here and see that cattle here have the disease, I will say that the cattle there are sick, the same as Orkipei because it is contagious.

MAN 4 When a cow is sick, he asks what drug you use to treat the ill cow. And which source of information do you use about the treatment of diseases and the use of drugs?

MAN 8 You asked, where will you get information,

R: Will you hear on the radio, through veterinary doctors, through chiefs, through the government announcement?

MAN 8 Saying that there is an outbreak of a disease.

R: Yes

MAN 8 Or when they announce a particular drug to be used.

R: Yes, all that general information about cattle. Is there a place you hear?

MAN 1 You do not know that the Maasai have their radio to exchange information. Like me now, if I go from here to Masaantare, I will go and talk like this: another comes to Lemek, and on arrival at Lulunga, we have another general talk, and he says that Lemek has some problem with cattle

MAN 8 Like that

MAN 1 Or there is some problem for people, they have their mode of talking and exchanging information well and plan, not your plan of putting on air on Radio Maa

MAN 8 For example, I arrived at this farm, and I started to talk with the head of the household about cattle

MAN 1 Even now, when you ask this old man where he is coming from, he will tell me all those problems about where he is coming from, another one like that. That is now our phone.

MAN 3 You see now, like this old man, he came from Olashapani, and now when we finish this discussion, he will tell us all the problems of Olashapani and everything he must say and us also we must tell him about here. When he goes to another place, he tells them that he went to Olgilai, and they say the place is this way and that way.

MAN 2 That is how our affairs go

MAN 4 Or when you meet another one from Lemek, you share information

R: I understand very well that when you meet as men, you must exchange information about the different places

MAN 1 That is now our phone

R: Is there a point where you will hear on the radio about...? And get their information

MAN 4 It is there...

MAN 1 We usually hear information on the radio. Even now, when I call with this phone someone in Aitong, I get this information, and I listen to it on the radio. You have heard that these Maasai radio stations have helped the people in a significant way.

R: Which radio station mostly does it teach you livestock issues?

MAN 1 Sidai FM, and another one Mayian. Those two are very okay for us. When news from Lemek happens, you hear it through Maasais's radio.

MAN 4 Mostly Mayian

MAN 1 Even Samburu they hear the radio stations of Maa

MAN 4 The old man is saying you visit those sides of Olashapani so that they get the information

MAN 1 You also get to sensitize them

R: Where is Olashapani?

MAN 1 This side of Olepolos, before you get to Olmekenyu

R: We will also add there and ask him if the disease, Oltikana, is also in Olashapani.

P10: Oltikana is not there, but Orkipei is the one that is there

MAN 8 You know Oltikana does not like a cold place; it likes hot areas

R: Have you heard of the vaccine for Oltikana, like in Lemek? Have you heard of a place where farmers are using vaccines to prevent Oltikana?

MAN 1 No

MAN 4 We have not heard any doctors coming with it

MAN 8 Ultimately, there is no vaccine for Oltikana. It has never been heard

R: I also heard that in the areas of Mau, when people migrate their cattle there, they complain of Oltikana infections. What do you see? Have you ever migrated your cattle to Mau?

MAN 1 We have taken until we have feared. When you take the cattle of this side to Mau, I once migrated 150 cattle there and only returned about 50.

R: They were infected with Oltikana as well?

MAN 8 Yes

MAN 1 I don't know even the nature of that disease there. Mau is different

MAN 6 When cattle from here go to Mau, things become different

R: It becomes terrible!

MAN 1 They die almost all

MAN 8 You see now, even now, during drought here, and they say there is plenty of pasture there, it is better you go and buy harvested grass there but not to take the cattle there. It is better to struggle to feed them with hunger than take them to those sides

MAN 4 You should follow up when you hear of any death cases; you take the samples and research them.

R: Right now, when you hear of the vaccine for ECF being sold at agrovets, let's say, like here in Lulunga, and it is being sold at 1000kshs(7.73$), would you be willing to buy the vaccine and vaccinate your cattle against Oltikana?

MAN 8 Yes

MAN 1 That is not to prevent but to treat when the cow is infected.

R: You have told me there are outbreaks of Orkipei, and you usually vaccinate, so there is also the vaccine for Oltikana, which can be there, and they sell it at 1000((7.73$). Would you buy it to inject your cattle?

MAN 1 To prevent?

R: Yes

MAN 1 That one will be expensive

MAN 3 There is a challenge because if a person has 800 heads of cattle, the syringe goes for 1000 for one cow. The person will have to use 800,000 to vaccinate all the cows, which is impossible. People will not be able to afford it due to the high cost.

MAN 4 You know, for example, if we have not gotten information about the doctors coming to vaccinate livestock, you will have to use your means for saving your animals so that you prevent them from being infected

R: So, you would want this vaccine if it is made available. How much are you willing to buy it at a place that will be affordable for you?

MAN 1 You see how the economy has gone up

MAN 8 It should be less than 50kshs (0.39$)

R: Less than 50kshs (0.39$)

MAN 8 Yes

MAN 4 Just a small token that will not disadvantage the farmer

R: What would you suggest?

MAN 4 I am not very far from 100

R: 100 is okay?

MAN 4 Yes

MAN 8 But I saw, if it is possible for the government to come to help people, they issue the vaccine for cattle, they issue the vaccine at 20kshs (0.15$)

MAN 1 I suggest 50

P9: 50.

R: And with Orkipei and Olkirobi, you told me you vaccinate your animals. How much do they charge to vaccinate against them?

MAN 1 120 up to 100kshs

MAN 8 Let's say 120

P9: It can be 130

MAN 1 That is what I am telling him. They were charging it at 120, but we would wish it dropped to 60, half of it. We have had hardship in reserve.

R: We have seen that for sure because we visit many villages daily

R: What would you want to be done so that you can manage this cattle disease?

MAN 1 The drug to be brought

MAN 8 The drug to be made available. And the drug is available to be effective so that when you inject cattle, it stays on the body of the cow for not less than six months. It should be six months and above

R: Any different opinion

MAN 7 If there is a vaccine that will come for the first time, people will be given it free to see if it is effective. Everyone will be willing to buy when they confirm that it will work well.

MAN 8 At the agreed price

MAN 7 And you will have surety.

R: What is the hindrance to using the vaccine for ECF?

MAN 8 We have not got

MAN 1 It has not been available.

MAN 8 The vaccine is not there; the one available is for Olkirobi Orkipei, and we are using the vaccine for Oltikana, but we have not got it.

R: Do you see if there are veterinary doctors available when you call them to treat cattle, do you see it?

MAN 1 The doctors are okay; they are helping us a lot

MAN 8 They are okay because the doctors we have, when you call him, he asks you first how you see the cow, the signs of the disease before he tells you the drug you can go to buy for first aid, or he comes himself.

R: The last question is...

MAN 4 My last one is there is a cattle centre that is built and put up in a particular centre, let us say on his farm, if he has space, so that when you need to vaccinate the animals, all the farmers will bring their cattle there for them to be vaccinated, so we want help on that

R: So, you need the help of a cattle centre. We are recording everything and will see what needs to be done. What other vaccine do you need?

MAN 8 For Olkirobi, for Oltikana to be available, and also for Ormilo in goats, if that can be available, it will help us a lot.

MAN 7 And this other called Olodua?

R: You need its vaccine?

MAN 7 Yes

R: Any other view to be added

MAN 8 If you have that you wish to add

R: I came to discuss this with you, then made the report, and will devise the interventions for the problems you mentioned later. When we come back, we will sit with you again and give you the answers. We will end there.

**THE END**
