# Supplementary material for: Beyond the jab: Unravelling the complexities of vaccine adoption for East Coast Fever in rural Kenya
Source: PLoS One. 2025 Jan 28;20(1):e0315906. doi: 10.1371/journal.pone.0315906 (PMC11774369; doi:10.1371/journal.pone.0315906)
Supplement: S1 Dataset — (ZIP) [file pone.0315906.s001.zip › Supporting information (R)/FGD/FGD 230613_1108.docx]

**FGD MEN 230613_1108**

**Researcher:** We will discuss the diseases the cows are infected with within this area. I have seen that you are farmers, and you know of these diseases, and each answer you give is correct. Could you tell me the months that the ticks usually affect the cows?

**Man 5:** In the dry season, especially this month of June. Also, In the short rains, the ticks may affect the cows.

**Man 3:** The ticks can affect the cows in short rains, but they are reduced when it rains for a prolonged period.

**Researcher:** Which months are these?

**Man 5:** June.

**Researcher:** Why does the number of ticks affecting the cows increase in the short rains?

**Man 3:** The ticks are hatched in the soil. So, in the short rains, that is the time that they hatch they increase.

**Researcher:** What do you do to control and manage the ticks in the months that the ticks are affecting the cows? Are there many, like now in June?

**Man 3:** We usually go through a lot of challenges. But we buy the acaricides and wash the cows at the end of the week. So, you will find that we wash the cows four times a month.

**Man 5:** This will also depend on your financial ability. If you can wash the cows four times a month, that is fine.

**Researcher:** This is when the number of ticks affecting the cows is many.

**Man 5:** Yes.

**Researcher:** Man 4, how many times do you wash your cows when the number of ticks affecting them is many?

**Man 4:** Twice a week.

**Man:** Once a week.

**Man 2:** Thrice in two weeks.

**Researcher:** Do you still wash the cows at the same frequency in the months when the number of ticks affecting the cows is few?

**Man 5:** No.

**Researcher:** Which month does it usually rain in this area?

**Man 5:** March.

**Researcher:** How many times do you wash the cows in March?

**Man 5:** Once a month or sometimes twice a month.

**Researcher:** Is there any difference in washing the cows twice amount in the rainy season?

**Man 3:** In the rainy season, we usually wash the cows once a month.

**Researcher:** Other than using acaricides, is there any method you use to control the ticks?

**Man 5:** Yes. We administer a specific drug called Ivermectin. When you administer that medicine, the ticks will not affect the cows.

**Researcher:** So, when you administer that medicine, you will not have to administer the pesticides again?

**Man 3:** You will, but it will reduce the ticks.

**Researcher:** Which one do you prefer between the acaricide and this medicine?

**Man:** The acaricides.

**Researcher:** Why do you prefer acaricide to medicine? Is it because of the cost?

**Man 5:** It is because it is effective.

**Researcher:** Are there different types of ticks?

**Man 5:** Yes, there are many types of ticks.

**Researcher:** Could you describe the different types of ticks?

**Man 5:** There are three types of ticks. There are the red ones that are usually on the ears of the cows. There is the brown one.

**Man 3:** It usually looks swollen.

**Man 5:** It looks brown. There is also one that has spots on the body and usually has a pungent smell.

**Researcher:** Is there any other type that you know, Man 2?

**Man 2:** No, I do not.

**Researcher:** What about you, Man 4?

**Man 4:** They are only those types.

**Researcher:** In the tree types you mentioned, which type is usually common in this area?

**Man:** They are all common in this area.

**Researcher:** Among those, are there some that have effects on the cows, or do they not have effects?

**Man 5:** Some have effects. The one that has the spots on its body has the impact. When they are on the goat’s body, they usually make them sick for up to a month.

**Man 3:** This is usually on the goats and the sheep. They are typically infected.

**Man 5:** When they are also on the anus of the cows, they usually swell at that region.

**Researcher:** This is the tick with the spots on the body.

**Man 5:** Yes.

**Man 3:** The tick with the spots on the body is usually more poisonous than the other ticks.

**Researcher:** Man 2, there is something that you wanted to say.

**Man 2:** I only wanted to say that.

**Researcher:** Which diseases are caused by the ticks to the cows?

**Man 5:** They cause Oltikana (ECF) and Ormilo (coenuruses).

*(People talking over each other)*

**Man 5:** The cows are mentally affected with this Ormillo

**Researcher:** Does this only affect the cows or the goats?

**Man 5:** They are all infected with it.

**Researcher:** What about Oltikana?

**Man 5:** They are also all infected with it. But mostly the sheep and the cows. There are few cases of the goats being infected with it.

**Researcher:** Is there any other disease the ticks cause in the cows?

**Man 5:** Those are the diseases that they usually cause.

**Researcher:** I would like you to mention the diseases that you know.

**Man 5:** Foot and Mouth.

**Man 3:** Anthrax.

**Man 4:** Olkipei (Contagious bovine pleuropneumonia (CBPP) and Contagious caprine pleuropneumonia (CCPP).

**Man 5:** Kububo (Brucellosis).

**Man 2:** Oltikana.

**Researcher:** Is there any other disease?

**Man 4:** Another disease called Ndorobo (sleeping sickness) is caused by Tsetse flies. The cows usually get thin even after eating a lot of grass.

**Researcher:** With Foot and Mouth, are all livestock infected with it, or is it specific to certain animals?

**Man 5:** All the livestock are infected with it.

**Researcher:** What about anthrax?

**Man 5:** The cows are infected with it.

**Man 2:** With Olkipei, the cows and the goats are infected.

**Researcher:** Are the sheep not infected with Olkipei?

**Man:** Yes.

**Researcher:** What about Kububo?

**Man:** The cows only.

**Researcher:** What about Oltikana?

**Man 5:** The cows are only infected with it.

**Researcher:** What about Ndorobo?

**Man 4:** The cows are only infected with it.

**Man 5:** The other livestock can also be infected with it when the Tsetse flies attack them.

**Man 2:** I believe there is a relation between Ndorobo and Oltikana.

**Researcher:** Which of these diseases are usually common in this area?

**Man 5:** Foot and mouth, Olomorooj, Kububo and Olkipei.

**Researcher:** there are four common diseases that livestock are usually infected within this area.

**Man 5:** Yes.

**Researcher:** I would like us to discuss about Oltikana. So, are the cases of Oltikana common in this area?

**Man 5:** Yes. The cows are usually infected with it.

**Researcher:** Starting with Man 2, are there signs and symptoms you will observe in the cows that will make you know they are infected with Oltikana?

**Man 2:** I usually observe that the cows have their hair standing.

**Researcher:** So, Man 3, what will you observe in the cows that will make you know they are infected with Oltikana?

**Man 1:** The eyes.

**Man 5:** They will have watery eyes.

**Researcher:** Any other signs and symptoms of Oltikana that you will observe?

**Man 4:** They will lose their appetite.

**Man 3:** The cows will be inactive, and you will notice they are just in the shade. And they will lose appetite. This will show that the cows are infected with Oltikana.

**Researcher:** What about them drinking water?

**Man 3:** The amount of water they drink will also reduce. They will stop drinking water and may even stay up to two days without it.

**Researcher:** With eating, do they stop eating, or do they just lose this appetite and eat some grass?

**Man 5:** They stop eating

**Man 3:** They may eat some grass in the evening, but not much.

**Man 4:** They will also stay in the shade for extended periods.

**Researcher:** So, these are the signs you will check for a cow infected with Oltikana?

**Man 4:** They will also produce a lot of saliva.

**Researcher:** What about the nose?

**Man 4:** They usually produce mucus.

**Man 3:** They produce stocky mucus, which is not normal for a healthy cow.

**Researcher:** Since you said that they will have watery eyes. Do they change colour when they are also infected with Oltikana?

**Man 3:** The eyes usually change in colour, and they become red.

**Researcher:** What contributed to the cows getting infected with Oltikana?

**Man:** The ticks.

**Researcher:** Which type of ticks causes the cows to be infected with Oltikana?

**Man 3:** The ticks that have the spots on their bodies.

**Man 5:** Also, the red ticks that are usually in the ears of the cows and also at the anus also cause them to be infected with Oltikana.

**Man 3:** The red ticks usually affect the cows in the same region and are usually numerous.

**Researcher:** So, when many cows are usually at one point, they can get infected with Oltikana?

**Man 3:** When there are many at that point, they may cause them to be infected with Oltikana.

**Researcher:** So, you have mentioned the ticks, and when the cows are many at one point, they may be infected with Oltikana.

**Man:** Yes.

**Researcher:** Is there anything else that may cause the cows to be infected with Oltikana?

**Man 3:** There are the Tsetse flies that are usually in the lakes and the damp regions. So, when they sting the cows, they will also get infected with Oltikana. This is because they have a long mouth.

**Researcher:** What about if they interact with the wildebeests and other wild animals?

**Man 3:** The wildebeest usually causes a very dangerous disease to the cows. There are months when the wildebeests give birth to the calves. When they do so, and the cows eat the grass from the region where it had given birth, they will be infected with that disease.

**Man 5:** That disease had no cure.

**Man 3:** When the cows are infected with that disease, the result is the death of the cows. So, the main sign of a cow that is infected with Engati they usually have prominent watery eyes, and they will have their hairs standing.

**Researcher:** They almost have signs similar to Oltikana.

**Man 5:** Yes. But with his, they will have the cracks on their backs.

**Researcher:** Though they are different?

**Man 3:** Yes.

**Man 4:** The only difference that they have is that this disease is incurable.

**Researcher:** Is this disease common in this region?

**Man 4:** Yes. During the time that we had not fenced our farms, the wildebeests that came to this area were numerous and at that time they would give birth to the calves. There would then be an outbreak of the disease three to four months after this. So, many cows usually die at this time.

**Researcher:** This is after they eat grass in the areas where they have given birth.

**Man 4:** Yes. They also say that when the cows eat the grass that has the fur of the calves of the wildebeest they are also infected with this disease.

**Researcher:** And the disease cannot be treated?

**Man 3:** Yes. We have never found the medicine for that disease called Entorobo and Engati (Malignant Catarrhal Fever) the disease is more severe than Oltikana.

**Man 4:** there is also no medicine for Oromilo the livestock just die. These are the most devastating diseases. At least if they would have a cure, we would say that it’s a bit manageable

**Researcher:** Let’s move on to Oltikana. When was the last time that a farmer reported their, cows had been infected with Oltikana?

**Man 5:** We have not yet heard of farmers reporting that their cows are infected.

**Researcher:** Is there a time that there are many cases of Oltikana?

**Man 5:** Yes. In the season that we are currently in there are usually many cases of Oltikana.

**Researcher:** In June?

**Man 5:** Yes. Also, when there is plenty of grass, there are usually high cases of Oltikana at that time. But it’s been a long time since Oltikana has affected cattle in this area.

**Researcher:** Is there any other time that there are high cases of Oltikana in this area?

**Man 4:** This is when there is plenty of grass and the month of June.

**Researcher:** So, this is the month of June…

**Man I:** Also, in April.

**Researcher:** So, this is March and April also?

**Man 5:** Yes.

**Researcher:** Have the cows usually been infected with Oltikana majorly in these months or has it changed over the years?

**Man 5:** Previously there were many cases but currently, there are few cases of Oltikana.

**Researcher:** What do you mean by this?

**Man 5:** Currently, there are fewer cases of Oltikana than in previous years. Previously, many cows were infected with Oltikana and some even died of it. But recently, you may even stay for two years without your cows getting infected with Oltikana.

**Researcher:** Currently, why have the cases of Oltikana reduced than in the previous years?

**Man 4:** In the previous years, not many people washed their cows with acaricides. But currently, many people wash their cows frequently with acaracides which caused a reduction in the cases of Oltikana.

**Man 3:** Previously we usually had the challenge that many cows usually grazed the cows in one region for a very long time. So, during that time you may find that some cows are infected with Oltikana, and they are grazing with other healthy cows, and this caused the transmission of the disease to the other cows. But currently, since each of the farmers has their farms that the cows usually graze in, this caused a reduction in the cases of Oltikana. So, previously, grazing the cows in one area caused higher cases of Oltikana but recently since each farmer has their cows in their farms and the grazes them there have been reduced interactions between the cows lowering the cases of Oltikana in this area.

**Researcher:** So, currently the cows do not graze with each other and interact with each other?

**Man 3:** No, they do not.

**Man 4:** Each cow is on their farmer’s farm.

**Researcher:** Is there anything else that has caused the reduction of the cases of Oltikana or are those two the only reason?

**Man 3:** Those are the main reasons.

**Researcher:** So, are there different types of Oltikana?

**Man 3: Is a**ny disease different from Oltikana?

*(People talking over each other)*

**Researcher:** The different types of Oltikana such as Oltikana from this area are different from the one in Mara or other areas. When you migrate with your cows have your cows been infected with Oltikana?

**Man 3:** Oltikana from Mara is usually more dangerous than the one in our areas.

**Man 2:** It is usually very dangerous because of the different animals that are usually in that region.

**Man 4:** When the cows are infected with Oltikana in this area, you may treat the cows with only one dosage of the medicine but when they are infected with Oltikana in Mara, you mat tret the cows, but they will still end up dying.

**Researcher:** So, there is a difference between Oltikana from this area and Mara?

**Man 4:** Yes, a very big difference.

**Researcher:** What do the people in that area do so that they prevent their cows from being infected or treating them with Oltikana?

**Man 4:** In that area, the farmers usually use strong acaricides to wash the cows, and they wash them once a week.

**Researcher:** Are there different types of acaricides?

**Man 4:** Yes. So, they usually wash their cows with strong acaricides and then they also use many medicines to treat the cows when they are infected with Oltikana.

**Researcher:** So, there are also different types of medicine that they use?

**Man 4:** Yes. In this area, we only usually use Terramycin to treat the cows when they are infected with Oltikana, and they get cured of it. In that area, they may even treat the cows with three medicines at once.

**Researcher:** So, Oltikana from Mara is very dangerous to the cows?

**Man 4:** Yes.

**Researcher:** Is there any other type of Oltikana from another region?

**Man 4:** Yes. When we take the cows to Mau Forest, in the dry season, when we return them many of them die of the disease.

**Researcher:** They are also infected with Oltikana?

**Man 6:** Yes.

**Researcher:** So, before taking your cows to Mau or Mara, is there anything that you do to prevent the cows from being infected with Oltikana so that you cannot get the losses?

**Man 4:** If you were washing the cows once a week, when you go there you will wash the cows twice a week. Also, you will have to carry specific drugs other than Terramycin to treat these cattle.

**Researcher:** So, you usually increase the frequency of the number of times that you usually wash the cows?

**Man 4:** Yes. You will also have more effective medicines when you are in that area so that you can treat the cows if they are infected.

**Researcher:** So, other than Oltikana which other diseases are the farmers usually affected within this area?

**Man 4:** Olkipei.

**Man 2:** Kububo.

**Man 5:** Olkipei and Oltikana

**Man 1:** Olomoroj (Lumpy skin)

**Researcher:** Could you describe how the livestock are affected by Olomoroj?

**Man 5:** The cows’ goats and the sheep are usually infected with it. This usually makes their skin have sores and this even results in death.

**Researcher:** Is it common in that region?

**Man:** Yes.

**Researcher:** What about you Man 3?

**Man 3:** Kububo. The cows are usually infected with this disease in this area.

**Man 5:** Oromilo also affects us in this area and the sheep are mostly infected with it in this area.

**Researcher:** Among Oromilo, Olkipei and Oltikana which of the three diseases causes more losses to the farmers?

**Man 5:** Oromilo. This is because it does not have the medicine.

**Researcher:** How many days will it take for the cows to die of the disease when they are infected with Oromilo?

**Man 5:** After some time.

**Man 4:** It does not cause death to the cows. The cows may be infected with it, but they may stay for five months without showing the signs and symptoms then they will later.

**Man 5:** But they will be mentally retarded.

**Man 4:** They will be mentally retarded, and they will also eat a lot of grass. Over time they will lose weight, and they will become thin.

**Researcher:** Do they later die of the disease?

**Man 3:** They will later die of the disease.

**Researcher:** They cannot be cured of the disease.

**Man 4:** Yes.

**Researcher:** So, this is the most dangerous disease among the three?

**Man 4:** Yes.

**Researcher:** So, we have stated the most dangerous disease is Oromilo. What is the more dangerous disease between Olkipei and Oltikana?

**Man 5:** Oltikana. In this area there are hardly cases of Olkipei.

**Man 4:** But the goats are usually infected with it.

**Man 5:** Yes. But there are few cases of the cows being infected with Olkipei.

**Researcher:** Can Olkipei be treated?

**Man 5:** Yes.

**Researcher:** So, between Oltikana and Olkipei, which one will you spend more money to treat the livestock when they are infected with either of the two?

**Man 5:** Olkipei.

**Researcher:** So, I would like to get everyone’s opinion on this. So, what is the first thing that you do when your have observed that your cows have been infected with Oltikana?

**Man 5:** When the cows are infected with Oltikana. They usually have a low appetite, and they usually stay in the shade inactive. They will also have their hairs standing.

**Researcher:** So, after you have e observed all this what will you do?

**Man 5:** I will administer Terramycin.

**Researcher:** Do you usually have the medicine at home, or do you buy it when the cows get sick?

**Man 5:** I usually have the medicine at my place.

**Researcher:** What about you Man 4?

**Man 4:** I will administer the medicine then I will observe the cows. If they do not get better, then I will administer the medicine again.

**Researcher:** How long will you observe the cows to determine if they are getting better or if they are not then you administer the medicine?

**Man 4:** After two days.

**Researcher:** What about you Man 3?

**Man 3:** I will also do the same. After I have seen the cows are infected with Oltikana, I will administer Terramycin. When the cows do not get better after two or three days, I will administer Penicillin.

**Researcher:** What about you, Man 1?

**Man 1:** When the cows are infected with Oltikana, I will administer the medicine. When they do not get better than I will administer another medicine.

**Researcher:** The same medicine or different one?

**Man 1:** Different medicine. I may administer the white Terramycin, or the medicine form the veterinary doctor.

**Researcher:** So, you will call the veterinary doctor?

**Man 1:** Yes.

**Researcher:** What about you Man 4?

**Man 6:** I will also administer Terramycin.

**Researcher:** When the Terramycin is not effective, what do you do?

**Man 6:** I will administer another medicine.

**Researcher:** Since you have mentioned that you do almost the same thing the first time your cows are infected with Oltikana. So, what do you do after you have administered Terramycin and other medicines and your cows do not still get better, and the medicines are inefficient?

**Man 5:** If the cows do not get better, we still administer Terramycin. We may have to administer Terramycin for up to four days so that the cows’ condition starts to improve.

**Man 6:** Also, when the cows do not get treated for the disease then we sell them.

**Researcher:** Do you sell them to the slaughterhouse or other people?

**Man 6:** The slaughterhouse.

**Researcher:** Man 4, what do you do when the decline is not effective?

**Man 4:** I will just let the cows die. There are times when you will administer the medicine, and the cows will still die.

**Researcher:** After how long will the cows be severely affected by the disease that it will result to their death?

**Man 4:** After two weeks.

**Researcher:** So, they will hardly survive after two weeks?

**Man 3:** Yes.

**Researcher:** Have any of you heard of a vaccine that protects the cows from being infected with Oltikana?

**Man 5:** I have never heard of the vaccine that protects the cows from being infected with Oltikana.

**Researcher:** Man 6, have you heard of the vaccine that protected the cows from being infected with Oltikana?

**Man 6:** I have not heard of that vaccine.

**Researcher:** If you heard that there is a vaccine that protects the cows against Oltikana, and it costs one thousand five hundred shillings per cows can you purchase the vaccine?

**Man 5:** Yes, I will.

**Researcher:** Will you vaccinate all your cows?

**Man 5:** Yes, I will.

**Researcher:** What would be your reason?

**Man 5:** This is to prevent me from getting the losses when the cows die. This is because I would rather spend the thousand five hundred shillings to vaccinate the cows. After all, they cost approximately sixty thousand shillings. So, I would vaccinate the cows to prevent them from dying.

**Researcher:** What about you, Man 1?

**Man 1:** I can vaccinate the cows, but not frequently. This will depend on the ability of the people because you may find that some people have five dairy cows, and all have calves. So, it will be hard for them to sell the cows to get money to vaccinate the cows because they have the calves, So, this will all depend on everyone’s ability to buy the vaccine. This has been a challenge in purchasing the medicines in this area.

**Researcher:** Will you buy the vaccines for your cows and vaccinate all of them?

**Man 1:** I can but not frequently.

**Researcher:** So, this depends on the ability of the person?

**Man 1:** Yes.

**Researcher:** What about you Man 5?

**Man 5** I will also purchase the vaccine if it is available.

**Researcher:** Will you vaccinate all your cows?

**Man 5:** I will vaccinate all my cows to prevent them from being infected.

**Researcher:** Yu have said that you get challenges in getting the vaccine.

**Man 3:** Yes.

**Man 5:** We do not have challenges in getting the vaccine.

**Researcher:** What about the other medicines such as Terramycin and the others?

**Man 5:** We usually get the challenges.

**Man 3:** It prevents the cows from being infected with Oltikana. Because when the cows are infected with it, they may be treated with Oltikana, and they get fine.

**Researcher:** Terramycin is available in this area?

**Man 3:** Yes. It is in every agrovet that it is in this area.

**Researcher:** Man 6, if you hear that there is a vaccine that is sold in the agrovets in Ewaso Ng’iro costing one thousand five hundred shillings will you purchase it and vaccinate the cows?

**Man 6:** Yes, I will, and I will vaccinate all my cows.

**Researcher:** Even if you have fifty cows?

**Man 6:** Yes.

**Researcher:** Why is this so?

**Man 6:** This is to protect me form loos. Because the cows are usually sold at between fifty thousand shillings and eighty thousand shillings, so if you spend one thousand five hundred shillings only to vaccinate the cows than you will not incur losses.

**Researcher:** There are ties that the cows are usually infected with Oltikana though the cases are few. So, when the cows are infected with Oltikana, is there any way that this affects your business as many of you have told me that you are in the business of selling the cows?

**Man 5:** Yes. When the cows are infected with Oltikana they may die, and this causes the losses. If you had ten cows that you were to sell when they are infected with Oltikana and three of them die, this will affect your business.

**Researcher:** You had mentioned that when the cows are infected with Oltikana, and they do not get well even after treating them you sell them to the slaughterhouse.

**Man 4:** Yes, we sell them in the slaughterhouse but not at the same price as the healthy cow.

**Researcher:** So, the price will change?

**Man 5:** Yes. If the cow you sold at fifty thousand when it was healthy, now you will have to sell them at either twenty thousand shillings or even fifteen thousand shillings because e they are sick.

**Researcher 2:** Which other vaccines do you administer to the livestock?

**Man 5:** We administer the vaccine that protects against Olkipei, and we usually administer it on the tails.

**Researcher:** Is it medicine or a vaccine?

**Man 5:** It is a vaccine.

**Man 4:** The cows are usually administered the vaccines on the tails while the goats are injected on this area.

**Researcher 2:** Does Olkirobi have a vaccine?

**Man 5:** There is a vaccine.

**Researcher 2:** Where is it usually administered?

**Man 4:** We have never administered the vaccine to our livestock, though we recently heard of the vaccine.

*(People talking over each other)*

**Man 4:** We have never vaccinated our cows using that vaccine. But we have heard that it is expensive, costing one hundred shillings per cow. But for the Olkipei vaccine it usually costs between twenty-five shillings and thirty shillings per cow.

**Researcher:** There are two types of Terramycin?

**Man 4:** Yes.

**Researcher:** What is the difference?

**Man 4:** There is one that is 30% while the other on is 10%. There is also one called Adamycin and Alamcyin all of which are 30%.

**Researcher:** If my cows are infected with Oltikana, will I administer any of the medicines?

**Man 4:** You will have to administer the one that is 30% because this is the most effective.

**Researcher 3:** With the one that you administer through the tail, I have heard that it usually protects against seven diseases, is this true?

**Man 5:** This is usually for the goats and the sheep it protects them against seven diseases but for the cows it protects them against Olkipei.

**Researcher 3:** Which seven diseases are the goats and sheep protected against?

**Man 5:** We only know of Olkipei.

**Researcher:** Which challenges do you experience in getting information on the health of the livestock, including the vaccines, the medicines and other diseases?

**Man 5:** There are times that you go to the agro vet, and they will tell you that the medicine is not available at that time, and you should come the following week. While all that time the cows are sick.

**Researcher:** So, there are times that the medicine is not available?

**Man 5:** Yes. When the medicine is not available then the cows will either die or you will have to sell them at a loss.

**Researcher:** Is there any other challenge?

**Man 2:** The other change is there are times that we usually buy the Terramycin and store it in our homes until it expires. Sometimes, the cows do not get sick, so we do not use the medicine. So, the Terramycin reached the expiration date, and we are still using them because we do not know.

**Researcher:** When there are cases of Oltikana in this area, is there a place that you report, or is it easy to report that there are cases of Oltikana or any other disease in this area?

**Man 5:** We do not report the cases. When your cows are infected with Oltikana, you usually visit the veterinary doctor in Ewaso Nyiro to get the medicine. But when the cows are infected with Olkipei, we usually report this, and the farmers vaccinate their livestock.

**Researcher 2:** What major challenge do you experience with your livestock?

**Man 5:** Drought.

**Researcher:** So, this is when there is no grass or water?

**Man 5:** Yes.

**Researcher:** Is there anything that you may propose to be done so that you do not experience this challenge? Or should the government bring alternatives to how you can get the grass and the water?

**Man 5:** The government should make the animals feeds, and they should be accessible in the dry season.

**Man 3:** They should also dig a dam.

**Researcher:** Where do the cows and the other livestock get water in this area?

**Man 5:** In River Ewaso Nyiro.

**Man 3:** But during the dry season, it usually dries up. Two years ago, the river completely dried up during the dry season.

**Researcher:** What do you usually do at that time?

**Man 5:** We use the boreholes.

**Man 3:** So, we usually go through this challenge a lot.

**Researcher:** Do you usually go through any other challenges?

**Man 5:** The diseases affecting our livestock.

**Man 3:** When the goats are infected with Olkipei and not vaccinated, they may die of it.

**Researcher 4:** Many of you have stated that you treat your cows personally. So, have there been times that you called the veterinary doctors to treat the cows, or do you usually treat the cows by yourself?

**Man 5:** We only call the veterinary doctors to vaccinate the cows. However, we usually treat the cows when they are infected ourselves.

**Man 2:** It is costly to call the veterinary doctors.

**Man 5:** During an outbreak of the diseases, that is the time that we call the veterinary doctors. There was a time that we saw the government send veterinary doctors to vaccinate the cows and goats.

**Researcher:** Thank you very much for coming. I am done with the interview.

**[END]**
