# Supplementary material for: Beyond the jab: Unravelling the complexities of vaccine adoption for East Coast Fever in rural Kenya
Source: PLoS One. 2025 Jan 28;20(1):e0315906. doi: 10.1371/journal.pone.0315906 (PMC11774369; doi:10.1371/journal.pone.0315906)
Supplement: S1 Dataset — (ZIP) [file pone.0315906.s001.zip › Supporting information (R)/FGD/FGD 230621_1044..docx]

**FGD MEN 230621_1044.**

**Researcher:** In which months are your cows usually affected by ticks?

**Man 6:** In the dry season, when the cows graze, they may be infected with the ticks.

**Researcher:** Which months is the dry season usually experienced in this area?

**Man 2:** in January, August and September. These are the months that the grass has dried up.

**Researcher:** Has the trend of ticks affecting these cows changed over the years, or has it been this way?

**Man 3:** It has been expected that the cows will be infected with the ticks at these months. When it rains, the number of ticks usually declines, but when the grass dries up and the dry season begins, the cows will be infected with the ticks every two weeks or a month.

**Researcher:** So, has it always been a trend for the cows to be affected by ticks in those months?

**Man 3:** Yes.

**Researcher:** So, there are times when the number of ticks affecting the cows is high, while there are times when the number of ticks affecting the cows is low?

**Man 1:** Yes.

**Researcher:** Currently, there are fewer ticks.

**Man 2:** Since it started raining recently, the grass has not yet gotten green as it usually is during the rainy season, and fewer ticks affect the cows.

**Researcher:** How do you manage and control the ticks?

**Man 4:** We usually spray them with acaricides. Some farmers spray their cows after every two weeks, while some spray them after every month.

**Researcher:** How frequently do you spray your cows?

**Man 5:** After every two weeks.

**Researcher:** When there are numerous ticks in the dry season, how frequently do you spray your cows?

**Man 2:** Twice a month, this is after every two weeks.

**Researcher:** What about when the number of ticks affecting the cows is few?

**Man 1:** I spray the cows after every month.

**Researcher:** Do the ticks affect all the livestock or the cows only?

**Man 5:** They affect all the livestock, but the goats are more resistant to the ticks. So, the cows and the sheep are the most affected animals.

**Researcher:** Are there different types of ticks?

**Man 2:** Yes, there are. There is a type that is usually large, and they are white, and there are small red ones. There is also the one with spots; we typically know it is the most dangerous.

**Researcher:** So, are the other types not as dangerous as the ones with the spots?

**Man 4:** Yes. Even the large white one does not affect the cows lethally compared to the one with the spots.

**Researcher:** Do all three ticks affect the cows in the dry season?

**Man 5:** The white ones are the common ones, and after you have sprayed the livestock for one week, they will be on the animals. They are also not that dangerous to the livestock. However, with the one that has spots on its body, it usually affects the livestock. If it is on the limbs of the livestock, it can make the animals lame, and also, if you do not spray your livestock on time and they affect them, they can lead to the death of the sheep.

**Researcher:** Do they affect the sheep or the cows primarily?

**Man 1:** The issue with the sheep is especially those that have a lot of wool on their body. It is challenging for the farmer to observe the ticks on the animal's body, so they do not spray them at the required time, leading to the sheep's death.

**Researcher:** Are there diseases that the ticks cause in the cows?

**Man 3:** They usually cause effects to the animals, but currently, we do not know the diseases that the ticks cause.

**Researcher:** But the deadly one is the one with the spots on its body?

**Man 3:** Yes.

**Researcher:** Does it majorly affect the livestock in this area?

**Man 4:** They majorly affect the livestock during the dry season, though they do not majorly affect the livestock in this area.

**Researcher:** Does the use of the acaricide manage that type of tick?

**Man 1:** Yes, it does. They will be affected when you do not spray your livestock on time.

**Researcher:** So, with the cows, which three diseases are the cows usually infected within this area?

**Man 2:** Olkirobi, and currently, a year cannot pass without having an outbreak of the disease.

**Researcher:** Which month is this that there is an outbreak of Olkirobi?

**Man 4:** In March and the beginning of the year. Mostly, it is once a year and at that time.

**Researcher:** You could also state the diseases affecting all the livestock.

**Man 1:** Shamshami (Bluetongue virus) that affects the sheep.

**Researcher:** Is this the disease that causes mental retardation to the livestock?

**Man 6:** No, that disease is Ormilo (coenuruses) and usually affects the sheep and the goats.

**Researcher:** Does this disease affect the livestock in this area?

**Man 6:** Yes, it does, and the issue with the disease is that we do not have the medicine to treat it.

**Researcher:** So, the disease has no cure?

**Man 1:** No, it does not.

**Researcher:** Are goats and sheep the majorly infected animals with the disease?

**Man 3:** Yes. The cows are rarely infected with Oromilo. When the animals are infected with it, it results in their death. So, with the animals that are infected with it, you will either sell them or slaughter them.

**Researcher:** How does Shamshami affect the sheep?

**Man 4:** It affects the mouth of the sheep and makes them lose their appetite. Sometimes, the sheep infected with it die of the disease.

**Researcher:** Does Shamshami have a cure?

**Man 5:** There is the medicine we usually administer, and we say it treats seven diseases. So, we typically administer it to the sheep before they are infected with the disease. The disease spreads quickly, so when the sheep in *Naroosura* are infected, sheep from this area will also be infected. In 2020, there were high disease cases, and many sheep were infected with it.

**Researcher:** Have you encountered any cases of Oltikana?

**Man 6:** We also call it Malaria, and it affects both man and animals. With the animals, it affects their mouth and limbs. However, we usually see that the one involving the mouth is deadlier because the cows do not eat, cannot drink water, and produce froth. However, with the one that affects the limbs, the cows have sores on the hooves, which makes it difficult for them to move.

**Researcher:** What about the eyes of the infected cow?

**Man 3:** Sometimes, the cows will have watery eyes. They will have a rough hair coat. If you do not treat the cows early, it will affect them, and with the one that involves the mouth, since they will not have eaten for some days, it will affect them.

**Researcher:** So, with the cows that are infected with Oltikana and have all those signs, how long will they stay till they die, or will it not cause the death of the cows?

**Man 4:** If you have observed the signs of Oltikana on the cows and administered the medicine earlier, the disease will be diagnosed in three days. But if you do not treat the cow after three, it may take up to a week for the cows. However, with the one that affects the limbs, it usually takes longer to treat it.

**Researcher:** But Oltikana results in the death of the cows?

**Man 1:** Yes, it does. In the dry season, when they are infected with Oltikana, which affects their mouth, the cows will have a hard time eating the dry grass, which is usually challenging, but if they are infected in the rainy season when the grass is soft, they may eat some grass.

**Researcher:** So, there is no specific season in which the cows are prone to Oltikana infection, so can they be infected at any time?

**Man 6:** Yes. However, in the rainy season, the cases usually decrease because the rain washes their feet, and the grass is soft, which is what the cows usually feed on.

**Researcher:** What causes Oltikana?

**Man 2:** There is a specific type of fly when it stings the cow. There are specific months when the flies are numerous, so they transmit the infection when they sting the cows. Also, Oltikana is an airborne disease, so when the cows graze in the same area as the infected cows, they transmit the disease.

**Researcher:** When the cows graze with the wild animals, will they be infected with Oltikana?

**Man 4:** Ticks can also cause Oltikana, and the wild animals have the ticks and those flies. So, when you go to Mara there are many cases of Oltikana than this area because of the wild animals.

**Researcher:** Have your cows ever been infected with Oltikana?

**Man 4:** Yes.

**Researcher:** When was the last time that your livestock were infected with Oltikana?

**Man 6:** I don't remember well, but they were infected in January or March of this year. Though they were not severely affected.

**Researcher:** How many cows were infected with Oltikana at that time?

**Man 2: At** that time, when I heard that there was an outbreak, I administered medicine to the cows to prevent them from contracting Oltikana.

**Researcher:** So, each year, your cows must be infected with Oltikana?

**Man 1:** Yes. Every year, there is an outbreak of Oltikana.

**Researcher:** Also, in this area?

**Man 3:** Not really. But in Mara, there are many cases of Oltikana frequently.

**Researcher:** So, you have said that every year, there is usually an outbreak of Oltikana in this area. Has it been this way over the years, or has it changed recently?

**Man 5:** Previously, there were few cases of the disease. But in the last five years, there has been a decrease in the rate of infections. But you must administer the vaccine for other diseases, preventing the seven diseases against Oltikana and the one administered through the tail.

**Researcher:** So, have the cases of the diseases increased over the years?

**Man 2:** Yes. Each year, you have to treat your livestock for a disease.

**Researcher:** What is the reason why there are many cases of the disease currently?

**Man 4:** We have not gotten the reason why there are many cases of the diseases that the cows are infected with.

**Researcher:** When your cows are infected with Oltikana, does that affect your livelihood and your occupation?

**Man 6:** Yes, it does. If there is an outbreak and the cows are infected with it, and they cannot eat or graze, it will affect me. If I were to travel, I would not because I would have to take water to the affected cows and grass. If you are alone in the home and two cows are severely affected, you must take care of the infected cows.

**Researcher:** So, in Mara, there are high cases of Oltikana?

**Man 2:** Yes. Oltikana affects the cows and also the people are very high because of the wild animals.

**Researcher:** Is there a difference in the types of Oltikana affecting the livestock in Mara and the ones affecting the livestock in this area?

**Man 6:** We have been mostly told that the Oltikana affecting the livestock in Mara is more dangerous than the one affecting the livestock in this area. This may be because of the wild animals or the fly transmitting the disease is a different breed because we know it is more dangerous and even a person when they are infected with the disease if they are not treated on time they may die.

**Researcher:** What do the farmers from Mara do to manage and deal with Oltikana and even the farmers from this area when you have taken your cows to graze in Mara?

**Man 4:** In this area we rarely have the medicine to treat Oltikana but in Mara, if you are not keen your cows may be infected with Oltikana and it may kill your cows after one day. So, in Mara you should always have the medicine to treat the cows but in this area the cows can stay up to four days without getting affected by the disease.

**Researcher:** Do the farmers from Mara spray their cows more often than the farmers from this area or do they spray their cows at the same frequency as farmers in this area?

**Man 1:** The livestock will depend on the farmers. You will find that the farmers that do not spray their cows frequently are the ones that are severely affected but with those that have a strict timetable and they spray their cows weekly or after every two weeks their cows are not infected with Oltikana.

**Researcher:** Other than Oltikana, which diseases are the farmers mostly affected with in this area?

**Man 5:** Shamshami, Olkirobi (foot and mouth disease) and Ormilo.

**Researcher:** Which of these diseases causes the death of the livestock rapidly?

**Man 4:** Shamshami because in its outbreak it usually causes the death of many sheep. Also, the disease is hardly curable unless you administered the vaccine before the sheep were infected. With Olkirobi there is an outbreak but with Oromilo is rare and if the goats are infected with it, they do not transmit the disease to other livestock. But with Shamshami and Olkirobi they usually are transmitted among the livestock.

**Researcher:** Comparing the treatment of Shamshami and Oltikana, does the price of their treatment differ or is it similar?

**Man 3:** Shamshami affects the sheep while Oltikana mostly affects the cows. So, treating the cows is much more expensive than treating Shamshami.

**Researcher:** What is the first thing that you do after you have observed that your cows are infected with Oltikana?

**Man 2:** Most of the farmers after they have observed that their cows are infected with Oltikana, they usually administer the medicine.

**Researcher:** Which medicine do you administer?

**Man 1:** Terramycin 10% or Terramycin 30%.

**Researcher:** How many times do you administer the medicine?

**Man 3:** It will depend on the size of the animals, and we administer it for three consecutive days, especially in the evening.

**Researcher:** What do you do after you have administered the medicine for three days and it is not effective?

**Man 6:** At this time the cows are affected by the disease, and we call the veterinary doctors.

**Researcher:** Are there times when the veterinary doctors are not able to treat the cows of Oltikana?

**Man 2:** Yes. There are times that the veterinary doctors will examine the cows and treat them and the condition of the cows does not get better.

**Researcher:** What do you usually do after this?

**Man 4:** The veterinary doctor may have come to treat the cow and the following day the cows die.

**Researcher:** So, at that time you do not sell the cows?

**Man 5:** Yes. When the cows are infected with the Olkirobi it affects the meat.

**Researcher:** Are there herbal medicines that is used to manage the disease before administering the medicine or calling the veterinary doctor?

**Man 2:** There is no herbal medicine that is used to treat Olkirobi.

**Researcher:** What about for Oltikana?

**Man 1:** There is the herbal medicine that may be used to treat Oltikana in people but with the animals, there is no herbal medicine to treat Oltikana.

**Researcher:** Have you heard of any vaccine that protects the cows against Oltikana?

**Man 3:** Not yet. Currently, before the cows are infected with Oltikana the farmers usually vaccinate their cows as I had mentioned earlier.

**Researcher:** Is it the vaccine that is used to prevent seven diseases?

**Man 4:** That one is administered to the sheep but with the cows it is the one that is administered through the tail.

**Researcher:** Is the vaccine that is administered through the tail used to prevent the cows from contracting Oltikana?

**Man 1:** No, prevents other diseases and each year you will have to administer the vaccine to the cows.

**Researcher:** Does it prevent Oltikana?

**Man 2:** To some extent it does because when the cows are infected with Oltikana, those that had been administered the vaccine will not be affected as the cows that did not get vaccinated.

**Researcher:** Have you ever vaccinated your cows with that vaccine?

**Man 2:** With that vaccine you cannot administer it to the cows alone, we have to call the veterinary doctors to administer it.

**Researcher:** When the cows are administered this vaccine, they do not get infected with Oltikana?

**Man 1:** This reduces the cases of Oltikana. So, we have to vaccinate the cows with this vaccine before they infected.

**Researcher:** If you heard that there is a vaccine that will protect the cows from contracting Oltikana and it costs between nine hundred shillings and sone thousand two hundred shillings can you purchase the vaccine?

**Man 3:** The seller will give you the guarantee that they will not be infected with Oltikana. Since we have never seen the vaccine that protects the cows from being infected with Olkirobi it will be very hard to believe. But if the vaccine is available, then it will be very beneficial. However, the vaccine should not be expensive because it will financially challenge those with many cows.

**Researcher:** So, will you purchase the vaccine and vaccinate all of your seven cows?

**Man 4:** If the vaccine is available, it will be very beneficial. If we are given the guarantee that it will prevent the cows from contracting the disease because Oltikana kills the cows then we can buy it.

**Researcher:** So, you will buy it and administer it to your cows after you are sure of the vaccine and you are guaranteed that it will protect the cows?

**Man 4:** Yes.

**Researcher:** Since you have complained that the price is very high, what price would you recommend selling the vaccine?

**Man 5:** Currently, since we vaccinate our cows frequently and the cost of living is very high, the price should be affordable. It would have been better if the vaccine had been sold at one hundred shillings per head of cattle so that even the farmers with many cows could vaccinate their cows. However, with one thousand shillings for the vaccine, the farmers may be reluctant.

**Researcher:** So, if the vaccine is discovered and available, what would be the best means of passing the information on to the farmers in this region?

**Man 6:** In this area, we believe the village elders and the chief. The people usually follow the information given by the local authorities and reach many parts of this area. But when you instruct only one person to pass this information, then it may not reach everybody,

**Researcher:** So, are the chief and the village elders the best people to pass this information on?

**Man 2:** Yes. Many people will listen to them, and people from different parts of this area will also listen.

**Researcher:** What about other sources, such as the radio?

**Man 3:** With radios, there are usually many advertisements. But when they call for the farmers to go to a specific area to get their cattle vaccinated, the people may turn up, but it will not be all the farmers. But it would be better if the vaccine were available. They announce it through the radio and the chief because it will be more effective, and many people will listen to them.

**Researcher:** What are the challenges that the farmers in this area experience?

**Man 1:** The rain trends in this area have changed over the years. In previous years, the animals did not have a problem getting fat. But the rains have reduced, and the cows only get three months a week to feed adequately. So, the farmers experience the challenge of not having enough grass to feed their livestock. Also, there is one rainy season each year. So, the cows only feed correctly in April, May and June, but they do not have enough grass from the other months onwards.

**Researcher:** So, the main problem that you experience is drought?

**Man 4:** Yes. The other problem that the farmers experience is diseases. This is a challenge because livestock are currently prone to these diseases, unlike in previous years.

**Researcher:** Are there any other challenges that farmers experience?

**Man 2:** Those are the challenges that we mainly experience.

**Researcher:** Thank you very much. I am done with the interview.

**[END]**
