# Supplementary material for: Beyond the jab: Unravelling the complexities of vaccine adoption for East Coast Fever in rural Kenya
Source: PLoS One. 2025 Jan 28;20(1):e0315906. doi: 10.1371/journal.pone.0315906 (PMC11774369; doi:10.1371/journal.pone.0315906)
Supplement: S1 Dataset — (ZIP) [file pone.0315906.s001.zip › Supporting information (R)/FGD/FGD 230620_1042.docx]

**FGD Men**

**Researcher:** Which diseases are the cows usually affected within this area?

**Man 1:** Foot and Mouth, which cows usually infect. It is common in this area.

**Researcher:** Does it affect the cows, goats, sheep or all the livestock?

**Man 1:** It affects all the livestock.

**Researcher:** Which other diseases affect livestock in this area?

**Man 2:** Foot and mouth affect the cows' limbs and mouth, making them inactive and preventing them from eating grass.

**Researcher:** Could you mention other diseases?

*(People talking over each other)*

**Man 2:** Olkipei (Contagious bovine pleuropneumonia (CBPP) and Oltikana (ECF).

**Man 3:** *Inaudible.*

**Man 2:** I will translate what he says. He has said that diseases affect this area's cows, sheep and goats. Some of the diseases include foot and mouth and Oltikana. For the sheep, they are infected with Olomoroj (Lumpy skin) and Oltikana. The goats are infected with Olodua (Peste des petits ruminants (PPR), Olkipei(Contagious caprine pleuropneumonia (CCPP), and Oltikana.

**Researcher:** Oltikana affects which animals?

**Man 2:** Both the cows and the goats.

**Man 1:** The cows are the animals that are usually infected with it.

**Researcher 2:** What about Olkipei?

**Man 2:** The goats are usually infected with this disease.

**Researcher:** What about Olkirobi (foot and mouth)?

**Man 2:** The cows are usually infected with it.

**Researcher:** Are all livestock infected with it?

**Man 3:** Yes.

*(People talking over each other)*

**Researcher:** What about Olomoroj?

**Man 2:** The sheep and the goats are infected with it.

*(People talking over each other)*

**Man 1:** Recently, there has been a disease that has been reported called Shamshami that the sheep are majorly infected with, and it affects their mouths, producing a lot of saliva and froth.

**Researcher:** Comparing these diseases to Oltikana, are all the livestock infected with it in an outbreak, or is it one animal that is infected at a time, as treatment of the disease and the death rate of the diseases?

**Man 3:** *Inaudible.*

**Researcher:** Comparing these diseases to Oltikana, how do they affect the diseases?

**Man 3:** *Inaudible.*

**Man 2:** They have said Oltikana cases have existed for many years. When there is an outbreak of Oltikana, the animals interact with each other and transmit the disease to each other. This is also similar to the diseases that sheep are usually infected with, which we typically encounter challenges with.

**Man 3:** *Inaudible.*

**Man 2:** With Olomoroj, if my cows are infected with it, then my neighbours' cows will also be infected with it.

**Researcher:** What about how you treat these infections compared to Oltikana?

**Man 3:** *Inaudible.*

**Man 2:** During an outbreak, we usually get the vaccine and administer it to the sheep to prevent the disease from spreading in this area. If there is an outbreak of Olomoroj, we will vaccinate the sheep to prevent them from contracting the disease.

**Researcher:** Comparing the other diseases to Oltikana, what is the death rate of the different diseases?

**Man 3:** *Inaudible.*

**Man 2:** In an outbreak of Olkipei, this is the disease that affects the lungs. So, during this outbreak, since goats are the livestock that are usually infected, many goats generally die more than Oltikana. So, Olkipei has a higher death rate than Oltikana.

**Man 4:** Explaining more of what they have said. Since you are giving more bearing to Oltikana than the other diseases, comparing the death rate of Oltikana to Olkipei, Olkipei affects the sheep and the goats. Still, goats are frequently infected with it in this area. So, Olkipei is a contagious disease, so if your goats are infected with it, they will infect the goats of other farmers. Also, when a goat is infected with Olkipei on a farm, all the goats will be infected with it on that farm. Then, Oltikana, where one cow may be infected but will not cause the transmission of the disease to other cows.

**Researcher:**  So, comparing Oltikana to the other diseases, are cases of the diseases frequent in this area?

**Man 3:** *Inaudible.*

*(People talking to each other)*

**Man 21** There are two seasons: the dry season and the rainy season. So, there are seasons when there are more cases of Oltikana.

**Researcher 2:** What seasons are there higher cases of Oltikana?

**Man 3:** *Inaudible.*

**Researcher:** The question I am asking is, comparing the other diseases to Oltikana, how are the livestock infected with it in this area? The frequency of infections of the different diseases to Oltikana, say, with Olkirobi, the cows are infected with it every month?

**Man 1:** We usually migrate our cows to other regions in the dry season. There was a time in the dry season when we took our cows to Mara, and there were many cases of Oltikana in Mara. So, when we returned with our cows, many were infected with Oltikana. So, during the dry season, they are infected with Oltikana because they interact with the other infected cows and drink water from the same area as the infected cows. Thus, there was an outbreak of Oltikana. I do not know if I have answered the question correctly.

**Researcher 2:** All answers are correct.

**Man 3:** *Inaudible.*

**Man 1:** When the cows migrate to other areas, such as Mau, where it is very cold. Since this is a new environment for the cows and the climate is cold, they will be infected with Oltikana.

**Man 2:** According to how I have understood the question, I think this is the time it will take for the livestock to be infected with the other diseases compared to Oltikana. So, you can predict if the livestock will be infected with Oltikana. In this area, you would know some areas where you would take the cows, such as Mara, where there are many cases of Oltikana, or when you take them to Mau because of the cold climate, then they will also be infected with Oltikana. But with the other diseases, you cannot be sure when there will be an outbreak because it is usually random.

**Researcher:** So, it is hard to predict when the livestock will be infected with the other diseases but with Oltikana you can predict that when you take them to certain areas, they will be infected with it?

**Man 2:** Yes.

**Man 4:** When the climate changes then the cows get infected with Oltikana.

**Researcher 2:** Not if they go to a specific area that they are infected with Oltikana?

**Man 4:** No. It is when the climate changes.

**Researcher:** What causes Oltikana?

**Man 3:** *Inaudible.*

**Man 1:** The ticks and also the water that they drink. This is because they, may drink water together with infected cows thus transmission of Oltikana to the healthy cows. Also, when the cows graze in a different area, the leaves and the grass that they eat may cause them to be infected with Oltikana.

**Researcher:** Any other cause?

**Man 1:** Interactions between the wild animals and the cows will also cause them to be infected with Oltikana.

**Researcher:** Which signs and symptoms will you observe in a cow that is infected with Oltikana?

**Man 1:** The cows will have a swollen udder.

**Researcher:** Which other signs and symptoms will you observe?

**Man 1:** The cows will also have their hair standing. The cows will also have a reduced appetite, and they will not eat grass. When they are affected by the disease, they will stop eating grass and drinking water.

**Researcher 2:** Will there be changes in the eyes of the cows that are infected with Oltikana?

**Man 1:** There are some that the eyes will be sunken, and they will be watery. There is also a type of Oltikana that will make the cows cough.

**Researcher 2:** So, some cows will have sunken eyes, and they will be watery?

**Man 1:** Yes.

**Man 4:** Some cows will produce a lot of mucus, and they will have weak eyesight.

**Researcher: What** is the frequency of the Oltikana infections in this area?

**Man 1:** When there is an outbreak, not many cows are usually infected with it. There are reports of a few cases of cows that are infected with it, and it may be one cow in each farm not many cows are infected at once.

**Researcher 2:** Let me take you back on the signs and symptoms of a cow that is infected with Oltikana. So, you mentioned that they have watery eyes, and they produce mucus?

**Wazee:** Yes.

**Researcher 2:** How does the dung of a cow infected with Oltikana look like?

**Man 1:** there are times that the dung reduces. It is usually hard and has blood stains. There are times that it also has some mucus and is sticky.

**Researcher:** Have there been changes in the infection rate of Oltikana in this area over the years?

**Man 1:** Previously there were many cases of Oltikana and it usually caused the death of the calves. So, you would find that in a homestead, twenty calves would have died of Oltikana. But recently, when the cows are infected with Oltikana, the different types of cattle usually die of the disease.

**Researcher 2:** Do all of you agree with what he has said, or do you have different opinions?

**Man 2:** It has changed because many of the fares in this area have been fenced so they will stay in the fares. The cows will not interact with other cows from different farms thus this has reduced the cases of Oltikana in this area.

**Researcher 2:** Is there any other method that has contributed to the reduction of the cases of Oltikana?

**Man 3:** *Inaudible.*

**Man 1:** Previously, we did not wash the cows with pesticides frequently. However, washing the cows with pesticides has reduced the cases of Oltikana in this area.

**Researcher 2:** The pesticides in the previous years were not easily available.

**Man 1:** Yes.

**Man 4:** It was not common to wash the cows using pesticides but currently where we wash them frequently.

**Researcher:** So, are the cases of Oltikana, increasing, or decreasing or are they constant?

**Man 1:** They are reducing.

**Researcher:** What is the reason behind this?

**Man 1:** The reason that the cases of Oltikana have reduced is that we do not migrate with our cows as we did in previous years.

**Researcher:** What measures have you put in place to prevent your cows from being infected with Oltikana or treating your cows when they are infected with Oltikana?

**Man 3:** *Inaudible.*

**Man 1:** Washing the cows frequently with pesticides is a measure to prevent the cows from contracting Oltikana. This is because we have mentioned that it is caused by ticks, so, killing the ticks using pesticides will prevent the spreading of the disease. Secondly, the use of Terramycin to treat the cows. When you have observed the signs and symptoms of the cows infected with Oltikana then you administer Adamycin.

**Researcher 2:** Is this administered before they contract Oltikana?

**Man 1:** No. This is administered after you have seen the signs and symptoms of Oltikana on your cows.

**Researcher:** Which other measures have you put in place to prevent your cows from contracting Oltikana?

**Man 3:** *Inaudible.*

**Man 1:** The other method is the use of vaccines on the livestock. As farmers, we usually call the veterinary doctors to vaccinate our cows. An example is the vaccine that is usually administered through the tail, where we call the veterinary doctors, and they vaccinate all our cows. This is also a measure of preventing the cows from being infected with Oltikana, through vaccination.

**Researcher 2:** You have mentioned the vaccine administered through the tail.

**Man 1:** Yes.

**Researcher:** Is there any other vaccine that you have heard of?

**Man 3:** *Inaudible.*

**Man 1:** The other method of preventing the spread of the disease is that if there is an outbreak of Foot and Mouth, then you vaccinate your cows to prevent them from contracting the disease. Also, when there is an outbreak of Olkipei, you call the veterinary doctors, and they will calculate the price of vaccinating the cows or the goats and you have to vaccinate all of them to prevent them from contracting it. This is also similar to Oltikana.

**Researcher:** Are there environmental factors that cause frequent infections of Oltikana?

**Man 3:** *Inaudible.*

*(People talking over each other)*

**Researcher 2:** Like the way you had mentioned in Mau, there are higher cases of Oltikana. So, what are changes in the environment that cause the high cases of Oltikana in that area than this area?

**Man 1:** In this area, there is a salty place where the cows from *inaudible*, Ewaso Nyiro and Narosura. So, a few years ago, in this area, the cows were infected with all the diseases when they came to eat the salt. When you go to the area where they drink water then you will find all the sheep and the cows drinking water in that water point. Thus, the environment will contribute to the cases of Oltikana because of the water that we all share in this area and the area where we take the cows to drink salt that we also share with farmers from this area.

**Researcher 2:** Does anybody have anything to add to that?

**Man 4:** One of the causes of Oltikana is the interaction of the wild animals with the cows. Currently, we have wildlife conservations and the people, are fencing this farm thus this has caused a reduction in the cases of Oltikana in this area. This is one of the factors that mitigate the spread of Oltikana in this area.

**Researcher:** Why are there higher cases of Oltikana in Mara than in this area?

**Man 4:** This is because of the wild animals. When they interact with each other. We have said that currently there are fewer cases of Oltikana in this area but in the previous years when we used to take our cows to Mau to graze in the dry season, the grass and the leaves and the water from Mau and different from the ones in this area. I do not know if this is because of the climate of that area that has caused the changes but when the cows graze in that region they must be infected with Oltikana. But currently, we do not migrate with the cows frequently.

**Man 1:** Some of the farmers from this area have gone to Mara, and they have slaughtered three cows some five cows because they were infected with Oltikana. So, we hardly go to that area to graze our cows even if it rains in that area.

**Researcher:** What do you do in rearing the cows to prevent them from contracting Oltikana?

**Man 3:** *Inaudible.*

**Man 1:** In the shed where the cows are reared, when a cow is infected with Oltikana, it cannot transmit the disease to the other cows. So, we do not quarantine the infected cow because they cannot transmit the disease.

*(People talking over each other)*

**Man 1:** He is asking if your cows are infected with Oltikana, does sweeping the shed manage the spreading of Oltikana?

**Man 2:** I am asking the old man because he knows more of the disease. But he has said it is not helpful.

**Man 3:** *Inaudible.*

**Man 1:** He is suggesting that we do not isolate the infected cows. When a cow is infected with Oltikana we do not separate the cows because they cannot transmit the disease among each other.

**Researcher:** Does the age, breed of the cows or the condition of the cows affect their susceptibility to being infected with Oltikana. Such as, Oltikana affects the calves more than the other cows or cows that have a complication?

**Man 3:** *Inaudible.*

**Man 1:** The calves are the most vulnerable to Oltikana and they are the ones that are frequently infected with it.

**Researcher 2:** Could you state the age gap of those calves?

**Man 1:** From birth to one year old. Also, there are certain breeds of the cows, the Grade breed and they include Friesian and the other dairy cows. They are also susceptible to Oltikana.

**Researcher:** Are there different types of Oltikana that you know of?

**Man 3:** *Inaudible.*

**Man 1:** There are two types of Oltikana.

**Man 3:** *Inaudible.*

**Man 1:** There are two types of Oltikana, here is one that causes swelling of the salivary glands and the other one results in the watery eyes of the cows.

**Researcher:** How is the treatment of the two types of Oltikana?

**Man 3:** *Inaudible.*

**Man 1:** They have the same treatment.

**Researcher:** Which of the two diseases causes death at a higher frequency?

**Man 3:** *Inaudible.*

*(People talking over each other)*

**Man 1:** The one that makes the cows cough and have water eyes causes the death of the cows at a higher frequency when they are infected with it.

**Researcher:** What is the first thing that you do when you have observed that your cows are infected with Oltikana?

**Man 3:** *Inaudible.*

**Man 1:** Administering the medicine.

**Researcher 2:** Is there a specific medicine that you administer to your cows at that time?

**Man 2:** We administer Terramycin.

**Researcher 2:** Is there any other medicine that you administer to your cows?

**Man 4:** There is Terramycin, which is 10%, and the other one is 5%.

**Researcher 2:** When you have observed that your cows are infected with Oltikana, which medicine do you usually administer first?

**Man 4:** Terramysin that is 10%

**Researcher:** What is the reason behind this?

**Man 1:** This is because it is more effective.

**Researcher 2:** Are there guidelines that you follow in administering the medicine?

*(People talking over each other)*

**Man 3:** *Inaudible.*

**Man 1:** Previously we used to administer one dosage of the medicine. Currently, we administer two dosages on both sides of the cow. When the dosage is not effective then we administer two more injections.

**Researcher 2:** Previously you used to administer one injection but currently you are administering two injections. What made you increase the number of dosages, is to the change in the medicine or is it because of the cows?

**All Men:** Because of the cows.

**Man 1:** Previously we had smaller cows but recently we have larger cows that then require more dosages of the medicine when they are sick. So, previously the cows could not handle the two doses but currently, the cows can handle the two dosages at once because they are larger, healthier and stronger.

**Man 3:** *Inaudible.*

**Man 1:** He has also said that previously when the cows were infected with Oltikana and they were dairy cows they usually marked these ears with some irons for easier identification.

**Researcher 2:** What do you do when you have administered the medicine and then it is not effective after many attempts what do you do?

**Man 1:** After that then you just wait for the death of your cows.

**Researcher 2:** I would now like each one of you to share these opinions. So, when the medicine is not effective, will you wait for the cows to die, or will you do something else before they die?

**Man 3:** *Inaudible.*

**Man 1:** We do not sell.

*(People talking over each other)*

**Man 2:** When the medicine is not effective. I will just leave the cows because there are times when the cows usually get cured afterwards.

**Researcher 2:** So, you will leave the cows at that time?

**Man 2:** Yes. There is a possibility that the cows will get cured afterwards.

**Researcher:** What else do you do when the medicine is not effective?

**Man 4:** I may sell the cows.

**Researcher:**  Who will buy the infected cows?

**Man 4:** When I take the infected cows to the market, I accept whatever money I am offered.

**Man 3:** *Inaudible*

**Man 1:** When the cows have been severely affected by the disease, we usually cut the neck of the cows to shed the blood then we sell them.

**Researcher 2:** You usually sell them to the slaughterhouses?

**Man 1:** Yes.

**Researcher:** Why do you have to cut the cows at the necks?

**Man 1:** To shed the blood.

**Researcher 2:** What will you do?

**Man 2:** I will do as they have said. I will either sell the cows or just let them die at home.

**Researcher 2:** Do you sell the infected cows in the slaughterhouses, or do you sell it to people individually?

*(People talking over each other)*

**Man 4:** They are saying what they usually did in the past. Selling of the infected cows has reduced because currently you cannot take the infected cows to the slaughterhouse and sell them to them. After all, they usually test the cows.

**Researcher:** So, what will you do when the medicine that you have administered to the cows is not effective?

**Man 4:** I will wait for the cows to either die by themselves or they may recover. You may have administered the medicine numerously, but the disease persists but, in the end, they get cured of the disease.

**Researcher:** Which signs and symptoms will you observe in the livestock that will make you treat the livestock?

**Man 2:** There are times that I may go to the shed to open for the cows or the goats and then I may notice that one of either is inactive. This will make me know that they require treatment.

**Man 1:** You may also take them to graze, and you will observe that they have no appetite, and they are not eating grass.

**Researcher 2:** So, you will observe that they are inactive in the morning when you open for them to graze and also when you take them to graze, and they are not eating grass, then you will have to look for the medicine to treat the livestock.

**Man 1:** Yes.

**Man 3:** *Inaudible.*

**Man 1:** In the morning the first thing that you do is to go to the shed of the cows and observe all the cows even when they are asleep. There are some cows when they are asleep you can observe that they have a rough hair coat, and their hairs stand. So, you will have to wake the cow to examine it further, then you will know the disease.

**Researcher 2:** What time do you call the veterinary doctor to treat the cows?

**Man 1:** After you have administered all the medicines and then the cow's condition is not getting better then you call the veterinary doctor to treat the cows.

**Researcher:** In this area is there any problem in accessing the veterinary doctors?

*(People talking over each other)*

**Man 1:** As a farmer, you are the one who is supposed to treat the livestock when they are sick. When you are not able to do so and then it becomes a challenge then you call the veterinary doctors to treat your livestock.

**Man 1:** It is hard to access them because it is expensive. They are also very far.

**Researcher 2:** Are these services expensive or bringing them to your farm expensive?

**Man 2:** They are both expensive. You will treat your cows at first and then when the cows do not get better you will call the veterinary doctor to come and examine your cows and treat them and you will cater for all this.

**Researcher:** What are your sources of information on the animal's health how you can treat them and the various medicines for the livestock?

*(People talking over each other)*

**Researcher 2:** Since the veterinary doctor is your last option, where do you get information on the diseases?

**Man 2:** The local media. Through the radios where they may tell us that there is an outbreak in this area and so we should protect our livestock. So, this information is easily accessible because we usually listen to the radio, and it is the easiest method that we access the information.

**Man 4:** He has said what I wanted to say. Also, one of the primary sources of information is through observation of the livestock. There are times that you will observe the behavioral changes of the animals and other factors. There are times that we can even see a change in the dung, and we will know the medicine that we should administer. So, we go to the veterinary doctor, and we tell them the changes in the livestock, and then they give us the medicine that we administer. There is also a new method that the veterinary doctor has treat many cows in an area. They usually go to the radio stations, and then they announce that there is an outbreak. So, they take this initiative, and then we call them to vaccinate our livestock so that they are not infected with the disease. So, this is the latest source of information.

**Man 1:** Previously, we also had a mechanism to announce that there are some areas with an outbreak. So, we usually had the boundaries and announced that the cows from this area should not drink water from the common water point. So, when a farmer's livestock were infected with a disease, we usually demarcated the land and then told them that these animals would not interact with the other ones to prevent transmission of the disease.

**Researcher:** I would also like to know where the old man gets his information about the diseases.

**Man 3:** *Inaudible.*

**Man 1:** He has said there are two methods of getting the information or reporting the cases. When your animals are infected with a disease, you will go to the veterinary doctor, who will give you the medicine you will be administered. When there is an outbreak of a disease in this area, you will report it to the authorities.

**Researcher 2:** So, there is no challenge in reporting the cases of certain diseases in this area?

**Man 1:** Yes.

**Researcher 2:** I have asked this because there are areas where there is a challenge in reporting the cases. They will hear of an outbreak of the disease from a neighbour, and if they do not tell them, they will know how the disease is spread. So, is there no challenge in reporting the cases in this area?

**Wazee:** Yes

**Man 1:** We usually report the cases to the local administration, that is, the chief, who will then spread the information to other areas.

**Researcher:** After observing the cows' sick, do you usually attend to them yourself, or do you call the veterinary doctor?

*(People talking over each other)*

**Man 1:** After observing that all of your cows have the same signs and symptoms, they may have contracted a similar disease. So, we usually report this to the veterinary doctors. But if one cow is infected with a disease and you can manage it, then you take the personal objective and do so.

**Researcher 2:** Do you use herbal medicines to treat the animals when they are sick?

**Man 3:** *Inaudible.*

**Man 1:** We use some herbal medicines to prevent the livestock from contracting diseases. We usually give some trees for the calves, and then we …

*(People talking over each other)*

**Man 1:** He said that when you observe that your livestock can transmit the disease to each other, you call the veterinary doctors to vaccinate your cows.

**Researcher 2:** So, there is no herbal medicine that you will administer as a vaccine to your livestock?

**Man 4:** Yes.

**Researcher 2:** But there was the one that he was mentioning?

**Man 4:** The one that they are mentioning is not that it was used to prevent the cows from being infected with diseases but was to prevent certain supernatural things from occurring.

**Man 1:** In the past, the old men would call the herbalists to give the herbal medicine to prevent the cows from being affected by some taboos. But currently, we do not do it.

**Researcher 2:** So, currently, you just administer the medicine?

**Man 1:** Yes.

**Researcher 2:** Have you heard of the vaccine that protects the cows against Oltikana?

**Man 1:** When the cows are infected with Oltikana, and you go to the veterinary doctor, they usually come with medicine to treat the cows, but we do not know.

**Researcher 2:** I would like to know if you have heard of the vaccine that protects the cows from contracting Oltikana.

**Man 1:** We have never heard of it.

**Researcher 2:** If you heard that there is a vaccine that protects the cows from contracting Oltikana, and it is in the agro vets, you usually buy other medicines. It is sold between nine hundred and one thousand two hundred shillings per cow. Will you purchase the vaccine? I want all your opinions.

**Man 3:** *Inaudible.*

**Man 1:** He said it would depend on the person's ability to buy the vaccine because it has different costs. Some will not buy.

**Researcher 2:** What about you? Will you buy it if it is sold at one thousand shillings per cow?

**Man 5:** *Inaudible.*

**Man 1:** He has said that he will not buy the vaccine because that price is high for him

**Man 3:** *Inaudible*

**Man 1:** This one has said that when there is an outbreak of the disease, he must vaccinate all his cows. He will sell a cow to cover the expense of vaccinating the cows.

**Man 4:** I will do as he has said. But I will consider one factor: if my cows are not infected with Oltikana, then I will not vaccinate my cows, but when one of my cows is infected with Oltikana, I will have to vaccinate my cows. At that time, I will sacrifice one cow and sell it to vaccinate all my cows. Some farmers' cows may be infected with Oltikana, but your cows may not be infected with Oltikana, or one of your cows may be infected with it, but it will not spread to your other cows. Thus, I will only vaccinate my cows after they are infected with Oltikana.

**Man 2:** It will be hard for me to vaccinate my cows unless I have heard that there is an area where there is an outbreak of Oltikana.

**Researcher 2:** So, you will not vaccinate all your cows?

**Man 2:** No, I cannot.

**Researcher 2:** Many of you have said you will not vaccinate your cows. What would you recommend as the price of the vaccine?

**Man 5:** *Inaudible*

**Man 1:** He has suggested that the vaccine be sold at the same price as the other vaccines. Because we usually pay twenty-five shillings per cow for the vaccine we administer in the cows' tails, we should also pay the same price against Oltikana. If we pay one thousand shillings per cow, yet you have one hundred cows, then you will spend one hundred thousand shillings, which is very expensive.

**Man 3:** *Inaudible.*

**Man 1:** He suggests that there is the financial stability of the people. According to some, the price of one thousand shillings may be expensive, so we should just vaccinate the cows at the same price as we vaccinate the goats and the cows. Or you should vaccinate the cows at your own will.

**Researcher 2:** What about you? What would be your recommended price for the vaccine?

**Man 2:** *Inaudible.*

**Man 1:** He said the vaccine price should be similar to the other vaccines. Because when you look at the financial ability of the farmers in this area, one thousand shillings per cow is costly.

**Researcher 2:** Which price can you suggest? Twenty-five shillings may be too low for the cost to drop from nine hundred shillings.

**Man 3:** *Inaudible.*

**Man 1:** He has suggested one hundred shillings per cow.

**Man 4:** This question is tricky because when we fix the price at a certain point, and the people cannot afford the vaccine, they will not purchase it, and the cows will continue dying of Oltikana.

**Researcher 2:** What price would you suggest then?

**Man 4:** Fifty shillings per cow.

**Man 1:** I would also suggest fifty shillings per cow. This is because, during the vaccinations, I may have ten cows, and another farmer may have two cows. So, with this, the veterinary doctor cannot come to vaccinate the ten cows because when we call them to vaccinate the cows, they usually range from fifty to one hundred cows.

**Researcher 2:** So, bringing your cows together to get vaccinated, that is the time the veterinary doctors come to administer the vaccine?

**Man 1:** Yes,

**Researcher 2:** So, you would recommend that they charge for the vaccine when they are together?

**Man 1:** Yes. This is because this is what happens.

**Researcher 2:** So, at that time you should bring your cows collectively so that they can be vaccinated?

**Man 1:** Yes. At that time, when you do not vaccinate your cows then you will have to wait till next time.

**Researcher 2:** What about you, what would you recommend the price?

**Man 3:** *Inaudible*

**Man 1:** He also says that it should be sold at five hundred shillings. I would also recommend that they sell the vaccine at different measurements. So if you have fewer cows, you will purchase the vaccine according to the number of cows that you have.

**Man 3:** *Inaudible.*

**Man 1:** He has recommended that you should manufacture the vaccine and then bring it to us.

**Researcher 2:** So, in the beginning I heard you mention that the ticks cause Oltikana. According to you can the ticks be eradicated from this area?

**Man 4:** They cannot be eradicated.

**Researcher 2:** Why is this the reason?

**Man 4:** I think it is because of the rain. But they cannot be eradicated entirely form this area.

**Man 1:** In the rainy season, the number of ticks increases so that is why he is saying that the rain causes the ticks.

**Man 3:** *Inaudible.*

**Man 1:** He has said that there are seasons when the number of ticks increase. Also, the wild animals will depend on the number of ticks because when there are many wild animals in this area at a certain time than, the number of ticks also increases.

**Researcher 2:** So, in short, the ticks cannot be eradicated from this area?

**Man 1:** Yes.

**Researcher 2:** Does anybody else have a different opinion?

**Man 5:** *Inaudible.*

**Man 1:** He has said that they cannot be eradicated.

**Researcher 2:** Since you had mentioned the sources of information, how would you want the information on the vaccine to be passed?

**Man 2:** In this area, local media, such as radios, will be the best way to pass information because many of us listen to them. We have smartphones, but not many people have access to them.

**Researcher 2:** So, the first source is the use of the local media?

**Man 2:** Yes.

**Researcher 2:** Is anybody else with a different opinion?

**Man 4:** Adding onto what he has said, the use of the local authorities, such as the chief and the village elders, and also going to the local primary schools, you will cover many people. You may have announced it on the radio, but not everybody might have heard of it. But when you use the local authorities and then call us in the primary schools to talk to us, the people will tell others who were not there about the vaccine, or they may observe that they are vaccinating their cows with the vaccine announced recently. Thus, this will be effective.

**Man 1:** The use of community outreach and creating awareness. You may have meetings in the village to pass the information on to the villagers. When we use the village elders, you may not access everybody, but with the community outreach in the meetings, you will access many people and pass the information effectively.

**Researcher 2:** What if you pass the information through the veterinary officers?

**Man 4:** With the veterinary officers, we have that mutual friendship at a personal level, and when they tell us about the vaccine, we may conclude that they are marketing what they have. So, we may not even buy the vaccine because many of us do not trust them. So, we may not go for the vaccine because there are times when they inform us of a better vaccine, and when we administer it to our livestock, we still have the challenges of the diseases they told us it would prevent. So, it will be tough for us to buy the vaccine. Though we may buy it, we will not fully trust them.

**Researcher 2:** If the local authorities pass the information, will you trust them?

**Man 4:** Yes.

**Researcher:** Do you have a different opinion on that?

**Man 2:** No.

**Researcher 2:** What challenges will the people in this area experience in accessing the vaccine if it is present, or what will make the people in this area not purchase it?

**Man 4:** So, we are assuming that the vaccine is available, so what are the challenges you will have to go through to access the vaccine?

**Researcher 2:** Yes. As you have said, you cannot trust veterinary doctors. So, such challenges that you will go through that will make you not get the vaccine or administer it to your cows.

**Man 1:** Vaccine availability may be a challenge.

**Man 5:** *Inaudible*

**Man 1:** He has said that the price of the vaccine. If the vaccine is not sold at the price the farmers can afford, it will not be accessible to the people in this area.

**Man 3:** *Inaudible.*

**Man 1:** If we do not get the information on the vaccine at the right time, then we will not be able to access it. The resources also will affect this. Because you cannot buy the vaccine when your cows are infected with Oltikana at a particular time, and you do not have the money. The cost of transportation to purchase the vaccine is also a challenge. The nearest place we can access the vaccine is Ewaso Nyiro, and the fare to get there is expensive.

**Man 2:** The other issue is that veterinary doctors should administer vaccines in case of an outbreak of a disease. This is because when the veterinary doctor wants to vaccinate the cows, yet there are no cases of the disease, many people are reluctant to get the vaccine for this livestock. But if the veterinary doctors brought and administered the vaccine during an outbreak, many people would take it.

**Researcher 2:** When all this has been resolved, will many people access the vaccine?

**Man 2:** Yes. But the price should be fair; this is the priority.

**Researcher 2:** So, should the fair price be fifty shillings per cow?

**Man 2:** We recommended the fair price to be fifty shillings because the difference from nine hundred shillings was massive. But we would suggest that the price drop to twenty-five shillings per cow if possible; we are used to this. With fifty shillings, we would have strained a lot.

**Researcher 2:** Do you have anything to add on?

**Man 3:** *Inaudible.*

**Man 1:** He has said that getting the inaudible is challenging*.* So, we should get the information on the vaccine at the required time so that we can even sell the goats, if need be, to get the money to pay for the vaccine.

**Researcher 2:** What challenges do you face as a farmer besides diseases?

**Man 5:** *Inaudible.*

**Man 1:** He has said that other than Oltikana, a disease called Oromilo usually affects us in this area. In my opinion, there is also a disease called rabies. In this area, there have been cases of rabies being reported, and they affect the dogs, which then bite the cows and some children. And we have been told that it caused the death of two people. You will find that the people may have died of rabies. So, this is also a disease that affects us in this area.

**Man 3:** *Inaudible.*

**Man 1:** He has said that a disease is caused by the migration of wildebeests that affects cows. It is called Engati.

**Researcher:** Where do you take the cows in the dry season?

**Man 1:** We take the cows anywhere as long as it has rained in that area.

**Researcher:** So, you take the cows in any region long after it rained in that place?

**Man 4:** Yes. For example, if we are told that it has rained in Kajiado, we will take the cows to Kajiado.

**Researcher 3:** What if it has rained in Mara? Will you take your cows to Mara?

**Man 4:** Yes.

**Researcher 3:** Even if there are cases of Oltikana?

**Man 4:** Yes,

**Man 1:** Thank you very much for coming.

**Researcher:** Thank you, too, for coming for the interview. We are done.

**END**
