# Supplementary material for: Beyond the jab: Unravelling the complexities of vaccine adoption for East Coast Fever in rural Kenya
Source: PLoS One. 2025 Jan 28;20(1):e0315906. doi: 10.1371/journal.pone.0315906 (PMC11774369; doi:10.1371/journal.pone.0315906)
Supplement: Attachment — Submitted filename: review ECF v2.pdf [file pone.0315906.s004.pdf]

Very well revised manuscript and much improved. Comments of reviewers were addressed well. Some minor issues remain before it can be accepted:

1. There is a confusion between herds of cattle and heads of cattle.

A herd is a collection/group of animals. If someone has several herds, it means they have several distinct groups of cattle.

Heads of cattle are individual animals. Owning 5 heads of cattle means 5 animals.

Table 4 says “livestock keepers with fewer herd of cattle” – I think you mean heads of cattle.

L 362 in the quote you say - if a person has “800 herds of cattle” – I think you mean “800 heads of cattle”, i.e. 800 animals.

2. There is a confusing use of abbreviations for the disease and the vaccine against the disease.

For example, in L 107 FMD refers to the disease, while in table 4 FMD refers to the vaccine against the disease. Please go through the whole document and make sure this is clarified everywhere.

In Table 4 write: “existence of other livestock vaccines for FMD, CBPP …”

ECF vaccine or ITM vaccine? The Infection Treatment Method is a vaccine against ECF. Correct throughout so it is clear please.

Minor issues:

L 58 only define ITM once!

L 107 define FMD

L 128 – repetition in sentence – replace second livestock with animals.

L133 as above

L 201 remove “that”

L 204 add type of consent (written)

L 335 do you mean smaller herds?

L 376 correct repetition.

L 384 add “lower risk **of EFC**” “to other livestock vaccines **for diseases** considered prevalent”

L386 add “purchase **it**”

L 396 replace “flowed” with “spread”

L 427 replace “they” with “there”

L 447 what do you mean by “wildlife conservations” do you mean conservation areas?

L 455-458 revise sentence – makes no sense

L 564-581 What did the remaining 11% do? 14% prefer Mau, 75% prefer Mara and 11% prefer????

L 623 insert vaccines such as “**for**” FMD, CBPP….
