# Supplementary material for: Beyond the jab: Unravelling the complexities of vaccine adoption for East Coast Fever in rural Kenya
Source: PLoS One. 2025 Jan 28;20(1):e0315906. doi: 10.1371/journal.pone.0315906 (PMC11774369; doi:10.1371/journal.pone.0315906)
Supplement: Attachment — Submitted filename: review ECF.docx [file pone.0315906.s002.docx]

This is a good in-depth study based on a remarkable amount of data on socio-ecological factors influencing the perception of ITM for ECF of farmers in Kenya.

**General comments:**

The manuscript can be shortened by avoiding repetition, particularly in the introduction. Going from the general situation to the context in Kenya and then the particular study sites would improve readability.

What is the availability of vaccines in Kenya and can you mention how vaccines and awareness strategies usually reach the farmers? Livestock field officers, agrovet and self-administration, government interventions??

As Plos One is a journal with a readership from many different disciplines, it would strengthen the manuscript if certain terms were introduced (such as SEM, exotic cattle, grand breed etc.) better.

Please add a paragraph on the study team and some more info on data collection such as length of interviews. You mention that deep understanding of context reduced bias, so please explain who collected the data.

In the results section if would help if there were some quantitative indicators (i.e. majority of farmers, minority, some, a few, 23%) as to how many interviewees mentioned particular issues and what characteristics they had such as “village chief”, “small herd owner” etc. to allow the reader to judge the importance of their contribution.

Consider a sentence with a short summary statement after each section, such as “Mara is better for grazing during droughts, but provides more interaction with wildlife”.

Table captions should be clear enough to be understood standing alone. Therefore, please do not use abbreviations in the captions and add site of study.

Table 2: To improve readability, please put a line between all columns and maybe rename the column “participants” ”type of participant”. Aligning all text to the left would also make the table clearer.

Table 3: please do not use 130%. N=130 which should stand for 100%. Recalculate the percentages in the table accordingly.

Table 4: No abbreviations in the caption please. Line 314 it should read “heads of cattle”.

Table 5: why is it in colour? Continuity of format across all tables. Ormilo is heartworm, not heartwater, please correct. In the section for unpredictable, please change received to perceived.

The discussion has to be changed, so that referenced studies are not just included as numbers where they are part of a sentence. For each study used as part of a sentence, study site and relevant finding has to be spelled out.

Do your findings suggest that higher positioned farmers (chiefs, elders, larger herd owners) have more knowledge, better knowledge? This cannot be easily assumed from reading the results section, but can be clarified in the results section as mentioned above and picked up in the discussion.

Add a sentence on how influential community members could be used to lead and develop campaigns.

**Specific comments:**

Intro:

Please clarify:

Deaths are part of losses – reword

Typically HH with limited resources are affected – why?

Exotic cattle?

Economic losses – who and why?

Reduce repetition by starting with overall explanation of the complex interactions in East Africa and going to the specific situation in your study site. Make sure the described situation is more clearly applied to Kenya and your study population.

l. 289, replace “a few” with a number if possible.

L 357 last year is not “a few years ago”!

L 385 explain grade breed

L 399 to have diarrhea

L429 in 4 IDIs

L 600 explain ref 6 to complete the sentence like in l 609

L 618 punctuation

L 660-664 any comparison with other studies? Any reason why?

683 ECF tends to impact only smaller herds – why? Do you mean “the impact of ECF is felt stronger by owners of smaller herds”?
