# Supplementary material for: Beyond the jab: Unravelling the complexities of vaccine adoption for East Coast Fever in rural Kenya
Source: PLoS One. 2025 Jan 28;20(1):e0315906. doi: 10.1371/journal.pone.0315906 (PMC11774369; doi:10.1371/journal.pone.0315906)
Supplement: Attachment — Submitted filename: RESPONSE TO REVIEWERS.docx [file pone.0315906.s003.docx]

Dear Editor and Reviewers,
Thank you for the comments and suggestions on our manuscript We have carefully considered each point raised and made revisions to improve the quality of the paper. Below, we provide detailed responses to each comment.

This table details the comments/feedback received, the responses/ revisions made in the proposal, and the page number(s) where these revisions can be located in the proposal.

| **Manuscript section** | **Comments** | **Response** | **Line number** |
| --- | --- | --- | --- |
| Introduction | 1. The manuscript can be shortened by avoiding repetition, particularly in the introduction. Going from the general situation to the context in Kenya and then the particular study sites would improve readability. 2. What is the availability of vaccines in Kenya, and can you mention how vaccines and awareness strategies usually reach the farmers? Livestock field officers, agrovet and self-administration, government interventions?? 3. Please clarify:   Deaths are part of losses – reword (reworded lines 46-47)  Typically, HH with limited resources are affected – why? Economic losses – who and why?   4. Reduce repetition by starting with overall explanation of the complex interactions in East Africa and going to the specific situation in your study site. Make sure the described situation is more clearly applied to Kenya and your study population. | The introduction part has been revised to address these comments. It highlights ECF and impacts on livestock farmers in Africa; the presence of the ECF vaccine; low adoption rates in Southern Africa, Eastern Africa and Kenya; distribution and awareness of vaccines in Kenya; study gap and objective.  This statement has been included to highlight the different stakeholders involved for livestock vaccines reach famers and various awareness strategies employed. It also highlights the unique challenges ITM presents in relation to distribution and awareness creation.  This statement has been improved  This statement has been improved to highlight that livestock dependent communities are the most affected due to loss of livelihoods, food security and economic stability.  The introduction section has been reworked to improve this | 58-137  107-114  61  63-64  58-137 |
| Methods  Tables  Discussion  General comments | 1. Please add a paragraph on the study team and some more info on data collection such as the length of interviews. You mention that a deep understanding of context reduced bias, so please explain who collected the data. 2. In the results section it would help if there were some quantitative indicators (i.e. majority of farmers, minority, some, a few, 23%) as to how many interviewees mentioned particular issues and what characteristics they had such as “village chief”, “small herd owner” etc. to allow the reader to judge the importance of their contribution.   Table 2: To improve readability, please put a line between all columns and maybe rename the column “participants” type of participant”. Aligning all text to the left would also make the table clearer.  Table 3: please do not use 130%. N=130 which should stand for 100%. Recalculate the percentages in the table accordingly.  Table 4: No abbreviations in the caption please. Line 314 it should read “heads of cattle”.  Table 5: why is it in colour? Continuity of format across all tables. Ormilo is heartworm, not heartwater, please correct. In the section for unpredictable, please change received to perceived.  Table captions should be clear enough to be understood standing alone. Therefore, please do not use abbreviations in the captions and add site of study.  The discussion has to be changed, so that referenced studies are not just included as numbers where they are part of a sentence. For each study used as part of a sentence, study site and relevant finding has to be spelled out.  Do your findings suggest that higher positioned farmers (chiefs, elders, larger herd owners) have more knowledge, better knowledge? This cannot be easily assumed from reading the results section but can be clarified in the results section as mentioned above and picked up in the discussion.  Add a sentence on how influential community members could be used to lead and develop campaigns.  As Plos One is a journal with a readership from many different disciplines, it would strengthen the manuscript if certain terms were introduced (such as SEM, exotic cattle, grand breed etc.) better (reference done)  L 357 last year is not “a few years ago  L 399 to have diarrhea  L 600 explain ref 6 to complete the sentence like in l 609  ECF tends to impact only smaller herds – why? Do you mean “the impact of ECF is felt stronger by owners of smaller herds”?  Any comparison with other studies? Any reason why  Title of the paper; Wordy but unclear and not representative of the contents of the article; needs revision  As this is a qualitative study, it would be useful to describe the characteristics of the livestock keepers. For example, what kinds of people were mixed farmers or pastoralists and how are they differentiated in terms of age, education status? This is especially so because the paper clearly distinguishes between the perspectives of these different categories of farmers. What is mixed farming vs agro pastoralism  In the introduction, the authors describe vaccination. It would be helpful to know if this disease is treatable and if the treatment is easily available? If treatment exists, is it cheaper than the vaccines for example?  The study sites were selected based on contextual and ecological considerations. What were they? We do not see in this paper what this consideration for context and ecology provided in terms of enriching the data. For example, did those living close to wildlife areas discuss more the ecological aspects of SEM than those located in other areas?  In the results section, there is barely any contribution by the key informants to the study.  Not clear what the differentiation among the production systems is supposed to achieve? In the discussion section, it does not come up and in the results section barely.  82-83 Repetition as already stated in paragraph above  124 Table 1; What is the difference between agro pastoral and mixed farmers?  134 Are the elders the same people described as community leaders?  138 Gendered duties not gender duties  163 KIIs You describe animal health care service providers (both county and private) then talk about animal health service providers in agro vets. How different is this latter category from “private practioners earlier mentioned?  Were IDI participants different from FGD participants? Were the FGDs conducted according to the method of livestock farming ie pastoralists, agro pastoralists and mixed farmers? It appears so in the results but not mentioned in the methodology  180 Transition or translation?  247-248 Who were the livestock keepers who owned mixed breeds of cattle and thus more likely to adopt ITM? Were they people of a certain education or economic status?249-250 See comment above 247-248 are describe if there is any correlation    259-260 The last part of this quote does not relate to the kind of exposure described in the two quotes. I see two different kinds of exposure: exposure to the vaccine and exposure to ECF and its impacts  354-360 This paragraph is unclear. Cases of ECF are reported to be few and to have occurred a few years ago but the quote is about ECF cases the year before the study? For clarity this could be compared with the occurrence of other diseases. For example, ECF might have occurred but maybe not as impactful as other livestock diseases?  385 what does “the grade breed of cows” mean? Exotic breeds?  454-455 The description is about the weather and not climate  470 the quotes above describe this. This part needs to be restructured for clarity and to avoid repetition  493-530 This section is unclear. I think it would be better to not narrow down to one issue at a time eg migrating to Mara vs Mau as this is a simplifying a complex issue. It seems that as exemplified by the majority in line 506-507 that the livestock keepers are describing the complexity of mitigating against the ECF disease and other diseases in a context of hunger and drought. The narrative here simplifies this issue unhelpfully.  534 Policy factors; how is the focus of vaccination campaigns and the information available a policy factor? These would be if in the introduction section it is clarified that the policy direction in the country is towards some livestock diseases and not others.  689-691 are policy issues synonymous with public awareness campaigns?  708 Limitations after conclusion  719 Conclusion; Indeed, these issues are multifaced and there is complex interplay. However, the way they are discussed in this paper does not bring this out. It appears each factor is independent of the rest. This could have been mitigated through:  Please ensure that your manuscript meets PLOS ONE's style requirements, including those for file naming.  Please state what role the funders took in the study.  Please include this amended Role of Funder statement in your cover letter  Please provide a complete Data Availability Statement in the submission form, ensuring you include all necessary access information or a reason for why you are unable to make your data freely accessible.  You may seek permission from the original copyright holder of Figure 1 to publish the content specifically under the CC BY 4.0 license | This has been incorporated to provide details about the study team and how the team was selected to reduce bias. Additionally, information on the duration of the interviews has been included.  Percentages have been used as quantitative indicators. In addition, characteristics of the interviewees have been highlighted  Lines have been incoporated to make the table clearer  This has been addressed  Abbreviations have been removed from the caption  This table has been revised and format is the same as other tables. The other comments have also been addressed  We have revised the table captions to remove abbreviations and added the site of study to ensure that the captions are clear and can be understood independently  The discussion section has been reworked to incorporate this comment  This has been clarified in the result and discussion section  We have added a sentence to show influential community members could play a role in adoption of ITM  We have addressed this by providing clear definitions and explanations for terms.  SEM  Grade breed cattle  We have addressed by revising the text to accurately reflect the timeframe mentioned, clarifying that the most recent cases of ECF were within the past year.  The statement has been improved by adding have  We have addressed this comment by expanding on the explanation regarding livestock keepers' perceptions of susceptibility to ECF. We have added that the perceived lower susceptibility reduced the perceived need for vaccination, as they believed their herds were resilient to ECF.  We have addressed this comment by clarifying that ECF typically affects only a few animals within a herd, as opposed to other livestock diseases that can impact many animals or entire herds at once  We have addressed this comment by incorporating a comparison with another study which examines how pastoralists in northern Kenya use different knowledge networks to manage animal diseases under highly variable conditions.  The title of the paper has been revised and reflects the contents of the article  We have added a description of the characteristics of pastoralists, agro-pastoralists, and mixed farmers, based on prior studies and our own observations.  We have revised the introduction to include information on the treatment options for ECF and the cost comparison with vaccination.  We have revised the manuscript to elaborate on the contextual and ecological considerations that guided the selection of the study sites and how these factors enriched the data. The study sites were selected to capture diverse agro-ecological zones and production systems—pastoralism, agro-pastoralism, and mixed farming—each offering different contextual and ecological dynamics.  In response to your comment, we have now added more quotes and insights from the key informants to better reflect their contributions to the study.  We have clarified this in the results and discussions  The introduction section has been re written  We have highlighted difference has been highlighted in terms of livestock management practices and livelihoods derived from the different practices.  Yes, the elders referred to are the same individuals described as community leaders. To maintain consistency throughout the study, we have adopted the uniform term 'village elders' to refer to them.  This has been rectified  We have used the general term 'animal health providers' throughout. However, in the Results section, we have specified the type of provider, distinguishing between agro vet shop owners and veterinary professionals who operate outside of agro vets, to provide greater clarity.  Participants for the IDIs and FGDs were different, but their selection was based on the specific livestock production systems. The methods were chosen to collect data tailored to the type of information, with FGDs or IDIs used depending on the appropriateness for the data required. This has been clarified …..  This has been rectified to translation  The livestock keepers who owned exotic breeds of cattle were from regions outside the study sites. These individuals were not part of the communities where the study was conducted, as the local cattle population primarily consisted of indigenous breeds and cross breeds. This statement has been added  We have revised the manuscript and removed the last part of the quote to align with the type of exposure being discussed.  We have addressed the comment by clarifying the timeline of ECF occurrences and providing a comparison with other livestock diseases. The revised paragraph now emphasizes that while ECF is still present, it is perceived as less of a concern compared to other more impactful diseases. Additionally, we have included relevant quotes to illustrate this distinction.  Grade breed in the context of this study also means imported cattle. We have clarified that exotic cattle mean imported cattle breeds  This has been revised.  This has been revised. Quotes have remained but the explanation has been removed  We have revised this section to reflect the complexity of the challenges livestock keepers face, particularly in managing ECF and other diseases within the broader context of hunger, drought, and environmental stress. We removed any oversimplification of specific issues and restructured the narrative to better capture the interconnected factors affecting disease transmission and cattle health, as suggested.  We have moved the results and discussion around information to the section on knowledge and awareness of the vaccine.  Policy issues and public awareness campaigns are distinct. We have removed the sub section of policy factors and moved the contents to individual factors  This has been rearranged  The updated discussion now provides a deeper analysis of how these variables interact, highlighting their implications and drawing more meaningful connections between the findings and existing research.  We have formatted the document as per PLOS ONE guidelines  We have included a statement that the funder had no role to play in design, data collection and analysis, decision to publish, or preparation of the manuscript  We have included this in the cover letter  Thank you for your feedback. We understand PLOS’ data policy and have ensured compliance by including selected transcripts as supporting documents.  We (the authors) created the map (Figure 1), and we hereby publish it under the Creative Commons Attribution License (CC BY 4.0). This license allows unrestricted use, distribution, and reproduction in any medium, provided the original author(s) and source are credited. | 349-378  477-1150  346  445  462  971  267, 346, 445, 462, 971  1156-1540  496-549  1163-1172  1264-1266  120-126  889  769  902  1151  1210-1212  1166  1315-1320  1  271-292  66-76  145-292  492, 542, 547,590,596,842  703, 713, 1004,1272,1452,1533  57-137  271-292  Whole document  300  Whole document  349-356  388  496-549  547-549  767-779  890  1000, 1018  1029-1034  998-1073  586-600  1686  1159-1549 |
