# Supplementary material for: Beyond the jab: Unravelling the complexities of vaccine adoption for East Coast Fever in rural Kenya
Source: PLoS One. 2025 Jan 28;20(1):e0315906. doi: 10.1371/journal.pone.0315906 (PMC11774369; doi:10.1371/journal.pone.0315906)
Supplement: Attachment — Submitted filename: Response to Reviewers v2.docx [file pone.0315906.s005.docx]

Dear Reviewers,
Thank you for the comments and suggestions on our manuscript We have carefully considered each point raised and made revisions to improve the quality of the paper. Below, we provide detailed responses to each comment.

This table details the comments/feedback received, the responses/ revisions made in the proposal, and the page number(s) where these revisions can be located in the proposal.

| **General Comments** | **Response** |
| --- | --- |
| There is a confusion between herds of cattle and heads of cattle.  Table 4 says “livestock keepers with fewer herd of cattle” – I think you mean heads of cattle.  L 362 in the quote you say - if a person has “800 herds of cattle” – I think you mean “800 heads of cattle”, i.e.  800 animals. | We have thoroughly reviewed the manuscript and made the following corrections  In Table 4, we have replaced "livestock keepers with fewer herd of cattle" with "livestock keepers with fewer heads of cattle."  In Line 362, within the quote, we have corrected "800 herds of cattle" to "800 heads of cattle," reflecting that it refers to 800 individual animals. |
| There is a confusing use of abbreviations for the disease and the vaccine against the disease.  For example, in L 107 FMD refers to the disease, while in table 4 FMD refers to the vaccine against the disease.  Please go through the whole document and make sure this is clarified everywhere.  In Table 4 write: “existence of other livestock vaccines for FMD, CBPP ...”  ECF vaccine or ITM vaccine? The Infection Treatment Method is a vaccine against ECF. Correct throughout so it  is clear please. | We have ensured that abbreviations for diseases and their vaccines are clearly distinguished throughout the manuscript.  When referring to the disease, we use the abbreviation alone (e.g., 'FMD' for Foot-and-Mouth Disease).  When referring to the vaccine, we specify 'FMD vaccine' to indicate that it is the vaccine against the disease*.*  In Table 4, we have revised the text to read: 'Existence of other livestock vaccines for FMD, CBPP...' This explicitly indicates that we are discussing vaccines for these diseases.  We have corrected all instances for clarity |
| L 58 only define ITM once! | We have revised the manuscript to define ITM (Infection and Treatment Method) only once at its first occurrence. |
| L 107 define FMD | We have added the definition of FMD (Foot-and-Mouth Disease) at its first mention in the manuscript. |
| L 128 – repetition in sentence – replace second livestock with animals. | We have replaced the second occurrence of 'livestock' with 'animals' to avoid repetition. |
| L133 as above | We have reviewed the sentence and replaced the repeated word to enhance clarity. |
| L 201 remove “that” | We have removed the word 'that' from the sentence as recommended. |
| L 204 add type of consent (written) | We have specified in the manuscript that written consent was obtained from all participants. |
| L 335 do you mean smaller herds? | We meant 'smaller herds'. We have updated the text to reflect this accurately. |
| L 376 correct repetition. | We have corrected the repetitive wording in the sentence. |
| L 384 add “lower risk of EFC” “to other livestock vaccines for diseases considered prevalent” | We have revised the sentence to include 'lower risk of ECF' and specified 'to other livestock vaccines for diseases considered prevalent' for clarity. |
| L386 add “purchase it” | We have added 'purchase it' to complete the sentence as suggested. |
| L 396 replace “flowed” with “spread” | We have replaced 'flowed' with 'spread' for better readability. |
| L 427 replace “they” with “there” | We have replaced 'they' with 'there' accordingly. |
| L 447 what do you mean by “wildlife conservations” do you mean conservation areas? | We have updated the term to 'wildlife conservation areas' in the manuscript. |
| L 455-458 revise sentence – makes no sense | We have revised the sentence for clarity |
| L 564-581 What did the remaining 11% do? 14% prefer Mau, 75% prefer Mara and 11% prefer???? | The remaining 11% chose not to migrate their cattle to either Mau or Mara but opted to purchase hay to feed their livestock locally. We have updated the manuscript to include this information for clarity. |
| L 623 insert vaccines such as “for” FMD, CBPP.... | We have inserted the word 'for' to read: 'vaccines such as for FMD, CBPP...' |
